# The effect of non-pharmacological interventions on cognitive function in cancer: an overview of systematic reviews

**DOI:** 10.1007/s00520-025-09212-3

**Published:** 2025-02-04

**Authors:** Darren Haywood, Ashley M. Henneghan, Alexandre Chan, Raymond J. Chan, Haryana M. Dhillon, Maryam B. Lustberg, Janette L. Vardy, Moira O’Connor, Norah Elvidge, Evan Dauer, Oscar Y. Franco-Rocha, Shradha Vasan, James Murray, Megan Crichton, Helen Wilding, Susan L. Rossell, Nicolas H. Hart

**Affiliations:** 1 Human Performance Research Centre, INSIGHT Research Institute, Faculty of Health, University of Technology Sydney (UTS), Driver Avenue, Moore Park, Sydney, NSW 2021 Australia; 2 Department of Mental Health, St. Vincent’s Hospital Melbourne, Fitzroy, VIC Australia; 3 Department of Psychiatry, Melbourne Medical School, Dentistry and Health Sciences, University of Melbourne, Parkville, VIC Australia; 4 School of Population Health, Faculty of Health Sciences, Curtin University, Bentley, WA Australia; 5 School of Nursing, University of Texas at Austin, Austin, TX USA; 6 Department of Oncology, Dell Medical School, The University of Texas at Austin, Austin, TX USA; 7 School of Pharmacy and Pharmaceutical Sciences, University of California, Irvine, CA USA; 8 Caring Futures Institute, College of Nursing and Health Sciences, Flinders University, Adelaide, SA Australia; 9 Faculty of Science, School of Psychology, Psycho-Oncology Cooperative Research Group, University of Sydney, Sydney, Australia; 10 Yale University School of Medicine, New Haven, CT USA; 11 Faculty of Medicine and Health, The University of Sydney, Sydney, Australia; 12 Cancer and Palliative Care Outcomes Centre, School of Nursing, Faculty of Health, Queensland University of Technology, Brisbane, Australia; 13 Centre for Mental Health and Brain Sciences, Swinburne University of Technology, Hawthorn, VIC Australia; 14 Library Service, St Vincent’s Hospital Melbourne, Fitzroy, VIC Australia; 15 Exercise Medicine Research Institute, School of Medical and Health Sciences, Edith Cowan University, Perth, WA Australia; 16 Cancer and Palliative Care Outcomes Centre, Faculty of Health, Queensland University of Technology (QUT), Brisbane, QLD Australia; 17 Institute for Health Research, University of Notre Dame Australia, Perth, WA Australia

**Keywords:** Cancer, Cognition, Cancer-related cognitive impairment, Non-pharmacological, Intervention, Exercise, Cognitive rehabilitation

## Abstract

**Purpose:**

A significant number of cancer survivors experience cancer-related cognitive impairment (CRCI), which can impact their ability to think, reason, make decisions, and perform daily actions. In recent years, non-pharmacological interventions for CRCI have gained significant attention. These interventions include exercise, cognitive behavioural therapy, cognitive training/remediation, dietary, mind–body, and multi-modal/complex interventions. This umbrella review provides a critical overview to inform guidelines and current practice, identify the most promising interventions, and uncover gaps in the research literature.

**Methods:**

This umbrella review of systematic reviews was pre-registered on Open Science Framework and PROSPERO. Six databases were searched. Systematic reviews (SR) assessing any non-pharmacological interventions to improve cognition in cancer (any type) were included. The overview followed gold-standard guidelines and recommendations. The results were narratively synthesised, and descriptive statistics and effect size ranges were calculated.

**Results:**

Sixty-four (*n* = 64) SRs were included. Results were synthesised into four non-pharmacological domains. Cognitive training/rehabilitation had the strongest evidence for efficacy. Physical activity/exercise showed promising efficacy; however, the variability of findings was considerable. Mind–body and psychological/behavioural therapy interventions were limited, but there was evidence for short-term effectiveness. Multi-modal/complex interventions showed potential for improving cognition in cancer but were poorly defined.

**Conclusions:**

Overall, non-pharmacological interventions demonstrated efficacy for improving cognition in cancer. There were limited intervention characteristics within domains which were consistently related to efficacy. Three key recommendations are provided for future research: (1) adopt harmonisation and reporting guidelines; (2) develop definitional guidelines of cognitive domains for CRCI research; and (3) assess intervention and participant characteristics associated with positive versus null/negative findings.

**Supplementary Information:**

The online version contains supplementary material available at 10.1007/s00520-025-09212-3.

## Introduction

Up to 75% of cancer survivors (i.e. any individual living with, or beyond, a cancer diagnosis), report experiencing cognitive impairments that can impact their ability to think clearly, make decisions, and perform daily activities [1, 2]. This is commonly referred to as cancer-related cognitive impairment (CRCI) [1, 2]. CRCI is related to a range of notable negative outcomes for cancer survivors [1], and can significantly impact daily activities, occupational functioning, mental health, and social functioning [3–7].

CRCI can present prior to the commencement of cancer therapies, suggesting the cancer itself may directly contribute toward CRCI [2, 8]. Neuroimaging has shown reduced white matter volume in breast cancer survivors prior to treatment [9], and some have suggested peripheral inflammatory cytokines, commonly elevated as a result of cancer, may stimulate inflammation in the brain and impact cognitive functioning [2]. Furthermore, cancer treatments have long been understood to contribute toward CRCI. Chemotherapy, radiation therapy, targeted therapies, haematopoietic stem cell transplantation, endocrine therapy, and surgery have all been found to be related to CRCI [2, 10, 11]. Cancer therapies may be causal in the development and maintenance of CRCI through pathways such as neurotoxicity, inflammation, brain-derived neurotrophic factor changes, estrogen and androgen depletion, and immune system dysregulation [2, 12, 13]. Psychosocial challenges have also been proposed to be a bidirectional causal factor in the development and maintenance of CRCI [2, 3]. Cognitive impairment is a key domain of mental health challenges, including depression and anxiety, which are particularly common in cancer survivorship [3, 14, 15].

Beyond cancer and cancer treatment risk factors for CRCI, modifiable biological and psychosocial correlates of CRCI have also been identified in the literature (i.e. factors that can be targeted and manipulated through interventions) [16]. To reduce the severity of CRCI and minimise its impact, pharmacological and non-pharmacological interventions have been trialled in animal models and with human cancer survivors. Pharmacological interventions have resulted in mixed outcomes with CNS stimulants (e.g. methylphenidate), medications used for memory impairments (e.g. donepezil), and bone marrow supporting substances (e.g. erythropoietin) showing some promise, but highly variable efficacy in improving cognition in cancer [17]. Mixed outcomes of pharmacological interventions for CRCI may largely result from the uncertainty regarding the mechanisms of CRCI, and its variable efficacy has provided the impetus for strengthening a focus on non-pharmacological interventions, which have gained significant attention in recent years [17]. These include exercise, cognitive behavioural therapy, cognitive training and remediation, dietary, mindfulness, and mind–body interventions, and multi-modal/complex interventions [1, 2, 18]. Non-pharmacological interventions for CRCI are built on the premise of cognitive malleability and claimed to function across a variety of direct (e.g. neuroplasticity, cognitive compensation) and indirect (e.g. improved sleep, reduced inflammation, reductions in psychosocial distress) mechanisms [1, 2, 19–24].

There have been many systematic reviews (SRs) published examining the effectiveness of non-pharmacological interventions to improve cognition in cancer survivors of differing clinical and demographic characteristics. A broad understanding of the overall effectiveness of different groups of non-pharmacological interventions is essential to inform practice and future research. To provide an overall assessment of the effectiveness of non-pharmacological interventions to improve cognition in cancer survivors, an overview and synthesis of these SRs is required. This umbrella review aims to provide a critical overview to inform guidelines and current practice, highlight the most promising non-pharmacological interventions for use and further research, and identify gaps in the literature for future research. Therefore, the objective of this umbrella review is to provide a comprehensive overview of SRs examining the effectiveness of non-pharmacological interventions in improving cognition in cancer populations.

## Methods

This study was an umbrella review of SRs. This review type was chosen due to the large number of published SRs and meta-analyses on non-pharmacological interventions for cognition in cancer. The review was conducted in accordance with Smith et al.’s [25] guidelines for conducting umbrella reviews, Cochrane guidelines for SRs [26], and Preferred Reporting Items for Systematic reviews and Meta-Analyses (PRISMA) statement recommendations [27]. A prospective protocol was published on Open Science Framework [28] and registered with the International Prospective Register of SRs (PROSPERO ID: CRD42024524653).

### Information sources

Publications were identified through searches of six databases, all run on 14 March 2024: Ovid MEDLINE(R) ALL 1946 to March 12, 2024; Embase 1974 to 2024 March 12 (Ovid); Ovid Emcare 1995 to 2024 Week 10; APA PsycInfo 1806 to March Week 2 2024 (Ovid); CINAHL (EBSCOhost) and Cochrane Library (Wiley). Searches were not restricted by date.

### Search strategy

Search strategies were developed by a medical librarian (HW) in consultation with DH, a topic expert. Potential search terms were initially identified through an extensive exploration of subject headings and synonyms related to each concept. A set of 20 relevant publications identified through scoping searches (Supplementary Materials, Part 1) were mined for additional terms using Yale MeSH Analyzer [28] and used to validate the final search strategy.

Final search strategies combined the general concepts of Cancer AND (Cognition OR Cognitive Dysfunction) AND (Systematic Reviews OR Meta-Analyses) using a combination of subject headings and text words. In accordance with inclusion and exclusion criteria, excluded publication types were removed where possible in the search process, but no language or date limits were applied.

An initial search was developed for Ovid Medline and then adapted for other databases adjusting subject headings and syntax as appropriate (see Supplementary Materials, Part 1 for the full search strategies). Search syntax used in the Ovid databases was adapted for CINAHL (EBSCOhost) and Cochrane (Wiley) using the Polyglot Search Translator [29].

### Eligibility criteria

The eligibility criteria followed the PICOS (Populations, Interventions, Comparators, Outcomes, Study Design) framework [30]. **P:** humans or animals of any age, sex, or gender who have had a cancer diagnosis, at any stage of the cancer continuum, with any type of cancer, while receiving or having completed any type of cancer treatment. **I:** Non-pharmacological interventions to improve cognition. **C:** Any within or between-group comparison (i.e. to a control group, pre-post, alternative intervention, waitlist, etc.). **O:** Cognitive functioning (subjective and/or objective). **S:** Systematic Review. To facilitate the synthesis of the wider body of literature, it was not required that all participants within the primary studies be formally classified as having CRCI.

Types of studies included were SRs defined using the PRISMA-P statement definition of a SR [31]: (A) had an explicit aim; (B) used a systematic search strategy and selection of studies; and (C) systematically synthesised data using narrative synthesis and/or meta-analysis. Unpublished work, abstracts, letters, thesis, and conference proceedings were not included.

The inclusion and exclusion criteria are presented in Table 1.

**Table 1:** Inclusion and exclusion criteria for systematic reviews

Inclusion criteria	Exclusion criteria
1. Systematic reviews that examined the effectiveness of any non-pharmacological interventions in improving cognition in cancer populations	1. Systematic reviews that only included studies with pharmacological interventions (including supplements available over the counter) aimed at improving cognition
2. Human or animal systematic reviews	2. Systematic reviews that did not report on subjective or objective measures of cognition
3. Included population had been diagnosed with any type of cancer (current or previous), any stage of cancer encompassing the entire cancer continuum	3. Non-systematic reviews
4. All ages, sexes, and genders of participants included	4. Full-text not in the English language
5. Systematic reviews that included subjective and/or objective measures of cognition	
6. Systematic reviews that included any comparisons to establish intervention effectiveness (i.e. pre-post, control group, etc.)	
7. Quality of life (QoL) and other wellbeing systematic reviews in which at least 50% of included primary studies had a cognitive outcome measure	
8. Multiple population reviews where cancer was separately analysed/synthesised and at least 50% of included primary cancer studies have a cognitive outcome measure	

The inclusion and exclusion criteria were informed by previous SRs [e.g. 31] and developed to ensure inclusion of a wide range of SRs, such as those with cognition as one outcome domain among many, or SRs encompassing both pharmacological and non-pharmacological interventions.

### Study selection and data extraction

Study selection and data extraction followed the Overview of Reviews in Cochrane Handbook for Systematic Reviews of Interventions guidelines [26] and were reported as per PRISMA 2020 statement [27]. Search results were exported to EndNote bibliographic management software [32] and duplicates were removed by HW. In accordance with the inclusion and exclusion criteria, records were screened on publication type by HW within EndNote, and the following publication types were excluded: book or book chapter, case report, comment, conference proceeding, consensus development conference, editorial, letter, practice guideline, thesis, and trial registry record. All remaining records were added into Covidence systematic review software [33] for title and abstract screening.

Where SRs included a subset of primary studies or findings not relevant to the objective of this review (i.e. pharmacological interventions, outcomes other than cognition), only data from the primary studies that aligned with our inclusion/exclusion criteria were extracted.

Each record was independently screened on title and abstract in Covidence by two independent reviewers (DH, ED, OFR, and SV) with conflicts resolved by a third reviewer (AMH). Full-text records were assessed for eligibility by two independent reviewers (DH, ED, OFR, and SV) with any conflicts resolved by a third investigator (NHH).

Data were extracted from each included review by two independent investigators (ED, OFR, SV, JM, and MC), with inconsistencies solved by a third investigator (DH). Data extracted from the included reviews were: (1) review characteristics; publication year, title, authors, journal, review type, and country; (2) intervention characteristics; interventions included, length of intervention(s), human or animal, demographic characteristics, cancer types, cancer treatments, cancer stage(s), and comparison type(s); (3) measures; cognitive measures; and, (4) effectiveness; effective on cognition.

### Risk of bias (quality) assessment

Two investigators (from ED, OFR, SV, JM, and MC) independently conducted a SR quality assessment using the Assessment of Multiple SRs (AMSTAR) 2 Checklist [34], a 16-question tool that evaluates each item as ‘yes’ or ‘no’ and yields a final overall rating of ‘high’, ‘moderate’, ‘low’, or ‘critically low’ quality. Study quality disagreements were managed by consensus between authors and a third investigator (DH). The AMSTAR 2 Checklist has been widely used for overviews of systematic reviews, including for a multitude of overviews of systematic reviews in cancer [35, 36].

### Data synthesis

In line with best practice guidelines [37], data synthesis was performed using a narrative synthesis approach. Descriptive statistics and effect size ranges for meta-analyses were calculated. Standardised Mean Difference (SMD) was used as the measure of effect. No additional re-analysis of outcome data was conducted beyond overall review characteristics. The synthesis was conducted both across intervention types and within intervention types.

All relevant primary studies that were included are reported in the Supplementary Material, Part 2, including an indication of their occurrence in more than one review. The degree of overlap of relevant primary studies across the included SRs was calculated via the Corrected Covered Area (CCA) using the following formula; $CCA= \frac{N-r}{rc-r}$. *N* is the total number of primary studies, including any instances of multiple-counting from overlapping studies; *r* is the number of primary studies without multiple-counting; and *c* is the total number of SRs included in this review. The final CCA value is expressed as a percentage of overlap [38].

## Results

### Characteristics of systematic reviews

**Fig. 1:**
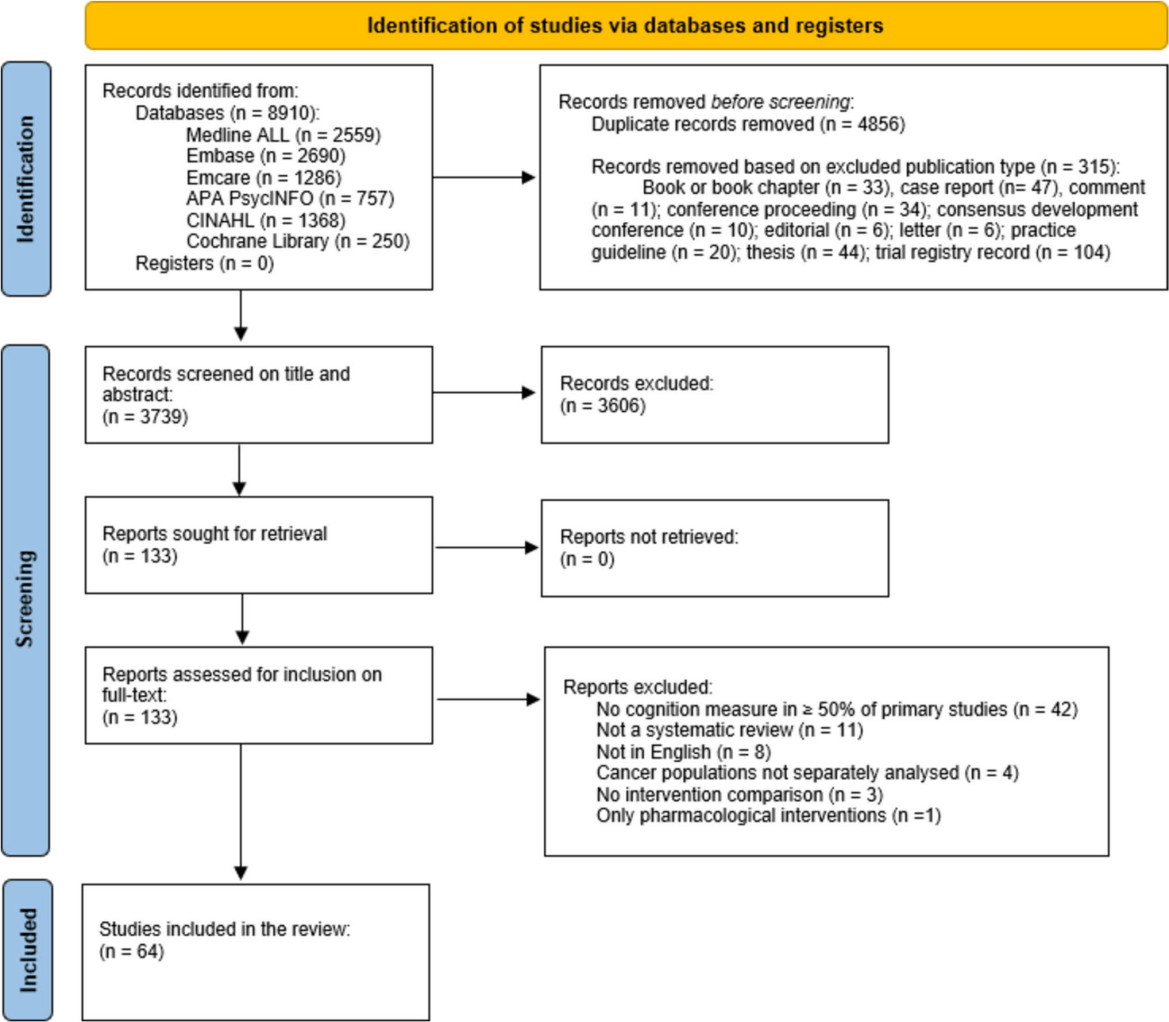
PRISMA flow chart

Overall, 8910 records were identified through database searches and 5171 records excluded before screening. After screening, 64 SRs were included in the narrative synthesis (see Fig. 1). The primary reasons for exclusion of full-texts were no cognition measure in ≥ 50% of primary studies (61%), not a SR (16%), and not in English (12%). The 64 SRs captured 318 individual primary studies relevant to our objective, totalling 30,847 participants (see Supplementary Material, Part 2 for primary studies). The number of relevant primary studies in the SRs ranged from 2 to 75. A total of 146 relevant primary studies were included in at least two of the SRs. The CCA determined an overall of 3% of the relevant primary studies, indicating a ‘slight overlap’ [39].

The characteristics and quality assessment of the 64 included SRs are summarised in Table 2.

**Table 2:** Characteristics of included SRs

Citation	Year	Number of relevant studies and type	Number of relevant participants	Available relevant participants characteristics	Cognition measure(s) type	Pre-intervention cognitive deficits	AMSTART quality rating
Akbari, P.S. et al. [40]	2023	RCT (16), longitudinal non-experimental (4), case-series (1), cross-sectional non-experimental (1)	1402	**Cancer types**: breast (12), breast and colorectal (2), leukemia (2), lung (1), ovarian (1), mixed (4) **Gender**: female 59%	Mixed	Not provided	Moderate
Alwi, S. M. S. et al. [41]	2021	RCT (6), quasi RCT (1), Single-arm trial (3)	589	**Cancer types**: breast (10) **Age**: mean 53.1(4.2), mean range 44.4–59 **Gender**: female 100%	Objective	Not provided	Low
Baydoun, M. et al. [42]	2020	RCT (6), single-arm trial (2), non-randomized comparative trial (1), case-series (1)	925	**Cancer types**: breast (8), mixed (2); stage 0–III (3), II–III (2), II (1), I–III (1), not provided (3) **Age**: mean 51(3.6) **Gender**: female (8 studies, 80%)	Mixed	Not provided	Low
Bergo, E. et al. [43]	2016	RCT (4), observational study (1), clinical trial (1), retrospective study (1)	314	**Cancer type**: brain (7); high/low grade gliomas, glioblastoma, brain metastases, meningioma, astrocytoma	Mixed	Not provided	Critically low
Bernal, J.D.K. et al. [44]	2023	RCT (16), non-randomized study intervention (6)	1277	**Cancer types**: lymphoblastic leukemia (4), CNS (4), mixed (14) **Age**: mean age at study starts 13.89 (5.29), mean age at diagnosis 8.19 (3.99) **Gender**: female 47.2%, Male 52.8%	Mixed	Not provided	Moderate
Binarelli, G. et al. [1]	2023	RCT (36), single-arm trial (9)	5223	**Cancer type**: breast (34), prostate (2), not reported (9)	Mixed	Not provided	Low
Binarelli, G. et al. [45]	2021	RCT (14), single-arm trial (6)	1564	**Cancer type:** breast (6), mixed (9), brain (4), prostate (1)	Mixed	Not provided	Moderate
Brunet, J. et al. Brunet and Sharma [46]	2023	RCT (53), quasi randomised trial (3) non-randomized trial (5), single group pre/post (7), longitudinal studies (4), case series/report (3)	7895	**Cancer types**: breast (33), mixed (20), haematological (7), brain (5), lung (4), prostate (4), rectal (1), skin (1) **Age:** mean 50.5 (15.2), median 52.7 **Gender:** female 72%, (54.7)	Mixed	Not provided	Low
Campbell, K.L. et al. [47]	2020	RCT (32)	3133	**Cancer types:** breast (14), hematological (7), prostate (2), mixed (6)	Mixed	Not provided	High
Caponnetto, P. et al. [48]	2024	RCT (5)	299	**Cancer types**: brain (5)	Mixed	Not provided	Low
Castellino, S. M. [49]	2014	RCT (1), single-arm trial (9)	278	**Cancer types:** mixed (5), unclear (2), brain (3) **Age**: range 5–22	Mixed	Yes	Critically low
Chan, R.J. et al. [50]	2015	RCT (7)	513	**Cancer types:** breast (7) **Age:** Mean 56.3 (8.18)	Mixed	Not provided	High
Cheng, A.S.K. et al. [51]	2022	RCT (27)	Not Provided	**Cancer types:** not provided (primary non-CNS tumour was part of the eligibility criteria)	Mixed	Not provided	Moderate
Cheng, X. et al. [52]	2018	RCT (7)	1110	**Cancer types:** esophageal (2), breast (2), endometrial (1), leukemia (1), colorectal (1)	Subjective	Not provided	Low
Cifu, G. et al. [53]	2018	RCT (5), single-arm trial (1)	638	**Cancer types:** breast (6); stage 0–III (2), I–III (2), not provided (2) **Age:** mean 53.1	Mixed	Not provided	Low
Drijver, A.J. et al. [54]	2022	RCT (4), quasi-experimental (1)	736	Not provided	Mixed	Not provided	Low
Dun, L. et al. [55]	2020	RCT (5)	Not Provided	**Cancer types:** colorectal (5) **Age:** mean 65.3 (13.98), range 18–69.3	Subjective	Not provided	Low
Egset, K.S. et al. [56]	2024	RCT (11), single-arm trial (6)	718	**Cancer types:** mixed (9), brain (7), leukemia (1) **Age:** mean 12.2; mean range 10.9–32.5 (overall range 6–39)	Mixed	Not provided	Moderate
Fernandes, H.A. et al. [57]	2019	RCT (13), quasi-experimental (2), single-arm trial (4)	1135	**Cancer types**: mixed (6), breast (11), prostate (1), not provided (1): cancer stages 0–III (2), I–II (4), I–III (3), not provided (10) **Age**: mean 56.62 (9.5), mean range 44.4–84.8, **Gender**: female (11), unclear (8)	Mixed	Not provided	Moderate
Floyd, R. et al. [58]	2021	RCT (10)	483	**Cancer types:** breast (10); stage I–III (10) **Age:** mean 52.56 (4.9), mean range 44.35–57.21 **Gender:** female 100%	Mixed	Not provided	Moderate
Fukushima, T. et al. [59]	2021	RCT (15)	1704	**Cancer types:** mixed (2), haematological (3), breast (8), prostate (1), esophagogastric (1) **Age:** mean age 53.5 (7.4), range 42.1–69.8) **Gender:** female (9), male (1), mixed (5; female ranged between 18.6% and 73%)	Mixed	Not provided	Critically low
He, F. et al. [60]	2022	RCT (9)	620	**Cancer types**: leukemia (5), brain (1), not reported (3) **Age**: 6–18 year-old paediatric cancer survivors	Mixed	Not provided	Low
He, K. et al. [61]	2023	RCT (4)	247	**Cancer types:** breast (4); stage I–III (2), not provided (2) **Gender:** female 100%	Mixed	Not provided	Low
Hiensch, A. E. et al. [62]	2023	RCT (16)	1987	**Cancer types**: breast (*n* = 1,053), male genitourinary (*n* = 364), haematological (*n* = 265), gastrointestinal (*n* = 123), gynaecological (*n* = 53), other (*n* = 20) **Age**: intervention: mean 54.7 (12.8); control: mean 55.3 (12.2) **Gender**: male 42% (*n* = 713)	Subjective	Not provided	Low
Hines, S. et al. [63]	2014	RCT (5), Single arm trial (1)	812	**Age:** mean range 49.19–60.42 years **Gender**: majority female (numbers not provided)	Mixed	Not provided	Low
Jesus, O.J. et al. [64]	2023	RCT (6)	285	**Cancer types:** breast (6); stage I–II (1), I–III (4), unclear (1) **Age:** mean 54.32 (5.51) **Gender:** female 100%	Mixed	Not provided	Low
Kasteler, R. et al. [65]	2023	RCT (4), quasi-experimental (6), single-arm trial (2)	499	**Cancer types:** brain (12) **Age:** at diagnosis, mean 7.04 (1.41), range 5.7–9.55; at enrolment mean 13.42 (4.01), range 9.45–23.85	Objective	Not provided	Low
Kim, Y. et al. [66]	2019	RCT (6), quasi-experimental (1)	714	**Cancer types**: breast (5) and mixed (2) **Gender**: female 98% (*n* = 702)	Mixed	Not provided	Low
Kirkman, M.A. et al. [67]	2022	RCT (3)	78	**Cancer types:** brain (3) **Age:** mean 52.83 (4.54), median range 48–53.5 **Gender:** mixed (2); between 57 and 60% male, not provided (1)	Mixed	No	Moderate
Kirkman, M.A. et al. [68]	2023	RCT (10), quasi-experimental (11)	1127	**Cancer types:** brain (21) **Age:** mean/median ages range 50–60 **Gender:** where it was reported, there was gender balance in most studies	Objective	Yes (9), not provided (12)	Low
Langenbahn, D.M. et al. [69]	2013	RCT (3), case-series (4), single-arm trial (4)	415	**Cancer types**: brain (11)	Objective	Not provided	Critically low
Liu, Y. et al. [70]	2023	RCT/Interventional (12)	818	**Cancer types:** breast (12) **Age:** all above 18 years **Gender:** female 100%	Mixed	Yes	Moderate
Mackenzie, L. et al. [19]	2022	RCT (45)	4727	**Cancer types:** breast (31), various (3), select group of cancer including breast cancer (11) **Age:** mean 54.6 (6.5), median 53.9	Mixed	Yes	Moderate
Mikkelsen, M.K. et al. [71]	2020	RCT (2)	378	**Cancer types:** breast and prostate cancer (1) mixed (1) **Age:** mean 75.5 (5.3), range 73–77 **Gender:** 59% female	Objective	Not provided	Moderate
Morean, D.F. et al. [72]	2015	RCT (3), longitudinal (4), prospective pre post (1)	332	**Cancer types:** breast (8) **Gender:** female 100%	Objective	Not provided	Low
Myers, J.S. et al. [73]	2018	RCTs (10), quasi-experimental trials (9), observational studies (1), case series (1)	2110	**Cancer types:** breast (12), mixed (6), breast and prostate (1), prostate (1), not included (1)	Mixed	Not provided	Low
Myers, J.S. et al. [74]	2015	RCT (9), Nonrandomized longitudinal (1), Case Series (1), Prospective (2), Semi-experimental intervention (1), Randomized double blind placebo control studies (1)	1125	**Cancer type**: brain (1), breast (10), mixed (4); stage not included (10), I–II (1), II–III (1), II (1), I–III (2) **Age**: no specifics, paediatric to adult	Mixed	Not provided	Critically low
Oh, P.J. et al. [75]	2016	RCT (14)	977	**Cancer types**: mixed (3), breast (7), glioma (1), brain (2), hematologic (1); stage not included (7), early stage (1), I–II (3), II–III (1), I–III (2) **Age**: mean 53.1, range 40 to 65 years (12), not included (2)	Objective	Not provided	Moderate
Oldacres, L. et al. [76]	2023	RCT (22), non RCT (4)	2333	**Cancer types:** breast (18), gastrointestinal (2), colorectal (1), lung (2), mixed (3) **Age:** all above 18 years	Mixed	Yes	Low
Park J.H. et al. [77]	2023	RCT (23)	1393	**Cancer types:** breast (23); stages 0–II (1), 0–III (4), 0–IV (2), I–II (1), I–III (11), II–III (1), not provided (3) **Age:** mean range 42.7–63.3 **Gender:** female 100%	Mixed	Not provided	Low
Persoon, S. et al. [78]	2013	RCT (5)	294	**Cancer types:** haematological (4), not provided (1) **Age:** range 39.1–48.8 **Gender:** female 36.6%	Subjective	Not provided	Low
Pfeiffer, S.M. et al. [79]	2018	RCT (5), pre-post (6)	272	**Cancer types**: brain (4), CNS (1), mixed (6) **Age**: mean 10.5, range 5–21	Mixed	Not provided	Low
Ren, X. et al. [80]	2022	RCT (12)	936	**Cancer types:** breast, stage I–III (12) **Age:** mean range: 46.7–67.8	Mixed	Not provided	Low
Sciancalepore, F. et al. [81]	2022	RCT (7), Open-label (2)	152	**Cancer types**: brain (5), mixed (4) **Age**: mean age 11.1–13.3 **Gender**: 32% female	Mixed	Not provided	Low
Sharma, B. et al. [82]	2021	Quasi RCT (2)	41	**Cancer types:** brain (2) **Age:** mean 11.84 (2.93) **Gender:** female 48%	Mixed	Not provided	Low
Simone, A. et al. [83]	2023	RCT (3), Prospective Cohort (1)	184	**Cancer types:** prostate (1), lung (1), mixed (2) **Age:** mean 69.83 (4.16) **Gender:** male only (1), male and female (3)	Mixed	Yes	Low
Singh, N. et al. [84]	2022	RCT (10)	2210	**Cancer types:** breast (6), breast and colorectal (1), brain (1), mixed (2)	Mixed	Yes	Critically low
Treanor, C.J. et al. [85]	2016	RCT (5)	235	**Cancer types:** breast (5) **Age:** mean range: 49.43 – 56 **Gender:** female 100%	Mixed	Yes (1), not provided (4)	Moderate
Van Lonkhuizen, P. J. C. et al. [86]	2019	RCT (5), single arm pre-post (4), 2-arm pre-post (2), 3-arm pre-post (1)	410	**Cancer types:** brain (12) **Age:** mean range: 39 – 60 **Gender:** female 49%	Mixed	Yes (6), no (6)	Critically low
Vance, D.E. et al. [87]	2017	RCT (10), prospective cohort (5)	961	**Cancer types:** breast (15) **Age:** mean range: 51–75	Mixed	Not provided	Low
Vannorsdall, T.D. et al. [88]	2021	RCT (11)	1067	**Cancer types:** breast (11) **Age:** mean 52.1 **Gender:** female 100%	Subjective	Not provided	Low
Von Ah, D. et al. [89]	2020	RCT(18), partially, quasi or non-randomized controlled trials (3), prospective cohort (5), retrospective study (1)	1543	**Cancer types**: brain (4), mixed (8), breast (12), gliomas (2), prostate (1) **Gender**: predominantly female (*n* = not reported)	Mixed	Yes	Low
Von Ah, D. et al. [90]	2014	RCT (12), Pre post (4), unclear (10)	Not Provided	Not provided	Mixed	Not provided	Critically low
Vuori, O. et al. [91]	2023	RCT (2)	121	**Cancer types**: brain (1), mixed (1) **Age**: mean 45.7–56.9	Mixed	Yes	Low
Wang, X. et al. [92]	2015	Not provided	536	**Cancer type:** lung (3)	Subjective	Not provided	Low
Wolfe, K.R. et al. [93]	2012	RCT (1), pre post (2)	182	**Cancer types:** brain/CNS involvement (3) **Age**: paediatric, age not provided	Objective	Not provided	Low
Yan, X. et al. [94]	2023	RCT (9)	666	**Cancer types**: breast (8), mixed but > 80% breast (1) **Age**: 50–60 years **Gender**: female (8), not provided (1)	Mixed	Yes	Low
Yang, H.Y. et al. [95]	2023	RCT (5)	242	**Cancer types:** breast cancer (3), breast and colorectal (1), lung cancer (1); stage I–IV (1), III–IV (1), I–III (3) **Age:** range 27–74	Mixed	Not provided	Low
Zeng, Y. et al. [96]	2016	RCT (7), cohort pre-post (3)	433	**Cancer types:** breast cancer (5), mixed (5) **Gender:** female (*n* = 234), female and male (*n* = 199)	Mixed	Yes (2), not provided (8)	Low
Zeng, Y. et al. [97]	2020	RCT (29)	2080	**Cancer types:** breast (18), breast and colorectal (1), mixed (8), gastrointestinal (1), prostate (1) **Age:** all above 18 years **Gender:** not reported (1089), female (211), male (60)	Mixed	Yes (1), not reported (28)	Low
Zhang, Y. et al. [98]	2017	RCT (4)	290	**Age:** all above 18 years	Mixed	Not provided	Moderate
Zhao, G. et al. [99]	2021	RCT (5)	510	**Cancer type**: gastrointestinal (5)	Subjective	No	Low
Zhao, K. et al. [100]	2020	Non-randomised studies of interventions (5)	262	**Cancer type:** glioma (5), stage I–IV (2), IV (3) **Gender:** female 43%	Objective	Not provided	Moderate
Zimmer, P. et al. [101]	2016	Animals: RCT (5) Humans: RCT (6), non-RCT (1), pre-post (3)	Animals: 226 Humans: 1307	**Cancer types:** animals: not provided; humans: breast (8), mixed (2)	Animals, objective; humans, mixed	Not provided	Low

In total, 44% of SRs included only randomised control trials (RCTs; *n* = 28), with two of the 64 reviews (3.3%) not including RCTs, resulting in relevant RCTs being included in 97% of SRs (*n* = 62). Most SRs included multiple cancer types (*n* = 36; 56%), with the most common cancers being breast (*n* = 39 reviews; 61%), followed by brain (*n* = 21; 33%), prostate (*n* = 10; 16%), colorectal (*n* = 6; 9%), and lung (*n* = 5; 8%). Haematological cancers were included in 10 of the 64 reviews (16%). Fourteen SRs reported the inclusion of studies whereby participants had pre-intervention cognitive impairment (22%), with 9 only including primary studies that had participants who showed pre-intervention cognitive impairment (14.06%).

The number of participants included within each SR ranged from 41 to 7895. Of the SRs that reported mean participant age ranges across their primary studies (*n* = 22; 34.4%), mean ages ranged from 5 to 75 years. Of the reviews that reported sex or gender (*n* = 29; 45.3%), most included multiple sexes or genders (*n* = 21; 32.8%) with only females being included in 11 of the 64 reviews (17.3%). Most reviews included both subjective and objective cognitive assessments (*n* = 48; 75%), with 9 of 64 reviews only including objective cognitive assessments (14.1%), and seven only including subjective cognitive assessments (11%). The specific cognitive measures included within each review can be found in the Supplementary Materials, Part 3.

### Quality appraisal of systematic reviews

The AMSTART quality ratings found the majority of reviews were of ‘low quality’ (*n* = 39; 60.94%), followed by ‘moderate quality’ (*n* = 15; 23.4%), ‘critically low quality’ (*n* = 8; 12.5%), and ‘high quality’ (*n* = 2; 3.1%). The mean AMSTAR quality rating across all reviews was 1.17 (SD = 0.68) reflecting an overall ‘low-to-moderate’ quality. A variety of quality appraisal tools were used in the included reviews, most commonly the AMSTAR and the Risk of Bias in Systematic Reviews (ROBIS) tool.

### Intervention characteristics and effectiveness

The characteristics of interventions and outcomes from the SRs are summarised in Table 3 across intervention domains. Most SRs included mixed intervention types (*n* = 24; 37.5%), followed by Physical activity/exercise (*n* = 15; 23.4%), cognitive training/rehabilitation (*n* = 15; 23.4%), mind–body and psychological/behavioural interventions (*n* = 5; 7.8%), and multi-modal/complex interventions (*n* = 4; 64.5%). See Supplementary Material Part 3 for additional details of the included reviews (i.e. countries, intervention descriptions, cognitive measures.

**Table 3:** SRs characteristics of interventions and outcomes

Citation	Year	Intervention type(s)	Comparator(s)	Assessment time points	Intervention cognitive outcome summary
*Cognitive training/rehabilitation*					
Alwi, S. M. S et al. [41]	2021	Cognitive training (4), compensatory strategies (5), cognitive and compensatory strategies (1)	Waitlist (6), control group (1), no comparison (3)	Multiple variations at two, three or four assessment time points	The majority of studies demonstrated improvements in verbal learning, working memory, processing speed, cognitive flexibility, verbal memory, inhibition of interference, attention, executive function and processing speed over various time points with small to large effects. Two studies demonstrated ineffective improvement of attention, memory, and cognition
Bergo, E. et al. [43]	2016	Neuropsychological training online (1), virtual reality (1), computer exercises guided by a neuropsychologist (1), holistic mnemonic training (1), cognitive training and rehabilitation (3)	No comparison (3), virtual reality (1), computerized treatment for different cognitive functions (1), usual care (2)	Multiple variations at two, or three assessment time points	Statistically significant improvements in auditory performances and digit span scores, improvements in neuropsychological assessments, attention, verbal memory, subjective cognition, compensation techniques, the Hopkins Verbal Learning Test, visuospatial memory, episodic memory, and phonetic fluency
Caponnetto, P. et al. [48]	2024	Virtual reality (2), computerized cognitive training (3)	Not provided	Four time points (1), not reported (4)	Computerised cognitive training showed small and moderate improvement in executive function
Castellino, S. M. et al. [49]	2014	Cognitive remediation (3), computerized cognitive program (3), group skills therapy (1), social skills (2), neuropsychological intervention (1)	Control group (1), pre-post (9)	Not provided	Statistically significant improvement in focused attention, academic achievement, attention and memory performance. One study reported no improvement in arithmetic computation
Fernandes, H.A. et al. [57]	2019	Strategy training (10), computer-based training (4), strategy and computer-based training (5)	Waitlist (10), active control (2), usual care (3), pre-post (4)	Multiple variations at two, four, or six assessment time points	Interventions affected objective cognitive measures such as memory, executive functions, processing speeds, global cognition. Clinical significance ranged from small (e.g. Cohen’s *d* ≤ 0.2) to large (*d* ≥ 0.8) Self-reported cognitive functions demonstrated improvement in subjective cognition; 78% of studies with self-reported data reported an intervention effect on one or more of these measures
He, F. et al. [60]	2022	Cognitive training (6), cognitive rehabilitation (1), neurofeedback (1), mathematics intervention (1)	Not Provided	Not provided	Improvements in working memory, attention, and executive functioning. Results for memory were statistically significant at postintervention assessment (*Z* = 2.24, *P* = 0.03), but not at 3/6‐month follow‐up (*Z* = 1.70, *P* = 0.09 and *Z* = 1.45, *P* = 0.15, respectively). Results for attention: significant statistical differences at postintervention and 3/6‐month follow‐up assessment; neurocognitive intervention reported SMD 0.81 (95% CI, 0.23–1.40; *Z* = 2.72, *P* = 0.007). Executive functioning post-intervention reported an SMD of 1.06 (95% CI, 0.34–1.77; *Z* = 2.90, *P* = 0.004) with a WMD of 1.76 (95% CI, 1.53–2.00) at 3/6‐month follow‐up
Kim, Y. et al. [66]	2019	Computerized cognitive training (6), computerized cognitive rehabilitation (1)	Waitlist (4), unclear comparator (3)	Multiple variations at two, or three assessment time points	Significant positive results in objective memory, verbal learning, executive function, letter fluency, and processing speed. Memory, and speed of processing domains showed significant increases in follow-up surveys, indicating long-term effects. Significant positive effects on subjective cognitive function. All studies showed significant improvements immediately after the program. Three studies found significant improvements in follow-up studies in subjective cognitive impairment, subjective cognitive ability, and memory
Langenbahn, D.M. et al. [69]	2013	Cognitive rehabilitation (11)	Waitlist (2), no treatment (1), other (8)	Not provided	Improvements in simple and vigilance attention, academic achievement, and parental ratings of attention. However, no gains on a test of arithmetic achievement, no significant between-group differences in neuropsychological functioning, for case studies/series, cognitive strategy interventions improved verbal working memory, attention, and arithmetic skills
Pfeiffer, S.M. et al. [79]	2018	Cognitive rehabilitation (5), cognitive training (6)	Pre-intervention data (6), wait list control (1), usual care (1), not provided (3)	Pre and post (7) and 3–6-month follow-up (4)	Statistically significant improvement reported in attention (4/7 studies), memory (4/7), executive function (1/1), and processing speed (1/1). Other studies found either no improvement or improvement without statistical analysis
Sciancalepore, F. et al. [81]	2022	Computer-based cognitive training (9)	Pre-intervention data (2), waitlist control (4), active control (3)	Pre and post (4) and 3–11 month follow-up (4) or 5-year follow-up (1)	Statistically significant improvement reported in memory (5/6 studies—1/6 not significant), executive functioning (2/3—1/3 not significant), processing speed (1/1), attention (3/3), patient-reported learning problems (1/1)
Von Ah, D. et al. [89]	2020	Cognitive rehabilitation (11), cognitive training (14), combined cognitive and compensatory training (1), combined cognitive training and cognitive rehabilitation (1)	Pre-intervention data (2), waitlist control (2), unspecified control (1), health training (1), not provided (2)	Pre and post intervention	Improvements in independence, productivity, memory, self-reported cognitive functions, subjective cognitive functions, attention, verbal fluency, processing speed, letter fluency, cognitive flexibility, executive function, and global cognition. Other studies reported no effect of intervention in memory, executive function, processing speed, psychomotor speed, attention, verbal fluency, subjective, cognitive flexibility, and global cognition
Vuori, O. et al. [91]	2023	Psychoeducation and strategy training (2)	Waitlist control (2)	Pre and post intervention	Statistically significant improvement in memory reported in one study. No statistically significant improvements reported in Wechsler Adult Intelligence Scale digit span, verbal fluency, cognitive functioning, executive functions, or patient reported fatigue and mood
Wolfe, K.R. et al. [93]	2012	Cognitive training (2), computerised cognitive training (1)	Waitlist control (1), pre-intervention data (2)	Pre and post intervention	Academics: improvement with intervention compared to control, p not provided (*n* = 1/2 studies), no effect of intervention (*n* = 1/2 studies); Attention: improvement with intervention, p not provided (*n* = 2/3 studies), no stat sig effect of intervention (*n* = 1/3 studies); Memory: improvement with intervention, p not provided (*n* = 1/3 studies), no effect of intervention (*n* = 2/3 studies)
Yan, X. et al. [94]	2023	Cognitive training (9)	Waitlist control (6), standard care (3)	Pre and post (5) and 2–6-month follow-up (4)	Subjective cognitive function: SMD 0.30, 95% CI: 0.08–0.51, *I*^2^ = 33%, *P* = 0.006, *n* = 5 studies, *n* = 6 interventions, *n* = 369 participants Objective cognitive function: attention: SMD 0.09, 95% CI: − 0.11–0.30; processing speed: SMD 0.28, 95% CI: 0.02–0.54; Verbal memory: SMD 0.32, 95% CI: 0.05–0.58; working memory: SMD 0.39, 95% CI: 0.17–0.61; episodic memory: SMD 0.40, 95% CI: 0.11–0.69; short-term memory: SMD 0.13, 95% CI: − 0.11–0.38 executive function: SMD − 0.15, 95% CI: − 0.30–0.20
Zeng, Y. et al. [96]	2016	EEG Neurofeedback (1), Cognitive rehabilitation (3), Psychoeducation (1), Cogntive behavioural training (2), Online home-based cognitive training (1), Memory intervention (2)	Waitlist (7), Control (2), Health training (1)	Pre and post intervention	Objective: Improvements in cognitive function (*P*-value < 0.05), neuropsychological status (WMD 5.66, 95% CI = 2.97–8.35), immediate memory (WMD 7.58 95% CI = 0.07–15.09), delayed memory (WMD 10.85 95% CI = 4.19–17.51), verbal learning function (SMD 0.50 95% CI = 0.19–0.81). No significant effects measured by digit symbol, digit span, and TMT cognitive tests. Subjective: Overall effect of cognitive rehabilitation (CR) − 0.19 (95% CI = − 2.98 to 2.61). The WMDs for the three subscales of PCI, PCA, and IPCIQL were − 0.76 (95% CI = − 18.90 to 17.38), 0.28 (95% CI = − 4.29 to 4.85), and − 1.50 (95% CI = − 4.59 to 1.60), respectively. There was no statistically significant difference (*Z* score = 0.13, *P* = 0.90). CT interventions had positive effects on improving the subjective cognitive function of cancer survivors in the follow-up evaluation (SMD 0.54 95% CI = 0.08–1.00; *Z* score = 2.29, *P* = 0.02)
*Physical activity/exercise*					
Akbari, P.S. et al. [40]	2023	Aerobic and resistance (13), aerobic (2), resistance (2), tracked physical activity (2), yoga (3)	No comparison (8), usual care (10), unclear comparator (2), lower intensity PA (1), muscle relaxation (1)	Ranged between 8 weeks and 2 years	Statistically significant small effect in social cognition (5 studies, SMD 0.23 (95% CI: 0.04, 0.42), *P* = 0.020, *Z* = 2.34) and objective attention (mean differences: − 0.29, 95% CI: − 0.48, − 0.09; 16.3, 95% CI: 1.4, 31.3). Improvements found in social cognition (calculated mean difference: 8.63, 95% CI: 1.11, 16.14), executive function and psychomotor speed (mean change: − 10.2, 95% CI: − 16.3, − 4.2). Three non-RCTs did not find within- or between-group differences in social cognition
Baydoun, M. et al. [42]	2020	Hatha yoga (4), individualized yoga sessions (1), Iyengar yoga (2), hatha and restorative yoga (1), unspecified yoga (2)	Standard care (2), supportive therapy (1), waitlist (2), exercise (1), CBT/self-hypnosis (1), no comparison (3)	Multiple variations at two, three or five assessment time points	High quality studies found positive effects of yoga on cognition, one only at the 3-month time point and two post-intervention (Cohen’s *d* = − 0.24 and 0.48, respectively). This was supported by non-statistically significant findings, as a general trend towards cognitive improvements were reported (Cohen’s *d* ranged between 0.38 and 0.74). Two other studies of fair and poor quality failed to show significant effects of cognitive function
Bernal, J.D.K. et al. [44]	2023	Coordinative exercise (1), mixed exercise (16), strengthening exercise (1), aerobic exercise (4)	No intervention group in ten (45%) studies and usual care in 12 (55%) studies	Not provided	Improvements in standardised cognitive performance measures (245 participants from 5 RCTs; Hedges’ *g* 0.40 [95% CI 0·07–0·73], *P* = 0.027; *I*^2^ = 18%) and patient-reported measures of cognitive function (890 participants from 13 RCTs; Hedges’ *g* 0·26 [95% CI 0·09–0·43], *P* = 0.0070; *I*^2^ = 40%). Studies that involved participants with a longer time since cancer diagnosis, a longer time since end of cancer treatment, more female participants, and lower levels of supervision were significantly associated with smaller improvements in cognitive function
Brunet, J. et al. [46]	2023	Aerobic endurance training (26), resistance/strength training (3), combined aerobic/endurance and resistance/strength training (24), yoga (7), qigong/tai chi (5), exergaming (2), aquatic-based (1), unspecified PA (3)^+^	Usual care (38), active comparison (10), waitlist control (8), add-on comparison (3), usual care and a healthy control group (2), single group pre-post design (5), case series design (2), single-group proof-of-concept sub-studies from a pilot RCT (2), case report (1). *	Not provided	Positive associations reported in reduced memory difficulty, mental fatigue and subjective and objective general cognitive functioning, response accuracy, conceptualization, mental flexibility, programming, sensitivity to interference, inhibitory control, environmental autonomy, subjective vocabulary, visual-spatial construction and increases in neural activity in several brain regions. The effects were inconclusive in 50 (71.4%) articles, and negative effects were reported for subjective cognitive abilities and general cognitive functioning
Campbell, K.L. et al. [47]	2020	Aerobic and resistance training (12), aerobic only (7), resistance only (3), mind–body exercise (7)	Not provided	Not provided	Statistically significant improvement in the exercise group compared with a control or comparison group. 2/9 trials that used objective neuropsychological testing reported statistically significant improvements in cognitive function using the Digit Span Forwards test and Auditory Consonant Trigram (Cohen’s *d* effect sizes 0.89 and 0.41, respectively)
Dun, L. et al. [55]	2020	Moderate intensity exercise (7), high intensity exercise (3)*	Individualized exercise program (1), supervised exercise (1), usual care (7), no exercise (1)*	Not provided	The results showed that the SMD was 0.51 (95% CI, − 0.33, 1.34), with no significant difference in cognitive effects between the intervention and control groups (*P* = 0.233)
Fukushima, T. et al. [59]	2021	Resistance exercise (5), aerobic exercise (3), resistance and aerobic exercise (7)	Not provided	Not provided	A meta-analysis from 15 RCTs revealed no significant differences in cognitive QOL between the groups (SMD = 0.11, 95% CI − 0.04 to 0.27, *P* = 0.16, *I*^2^ = 53%). A subgroup analysis of exercise types demonstrated no significant difference between the 3 subgroups (*P* = 0.92, *I*^2^ = 0%). In aerobic exercise (SMD = 0.07, 95% CI − 0.22 to 0.36), resistance exercise (SMD = 0.11, 95% CI − 0.29 to 0.52), and mixed exercise program (SMD = 0.14,95% CI − 0.09 to 0.38). RCTs in aerobic exercise and resistance exercise showed no interventional effects at high or moderate intensity
Hiensch, A. E. et al. [62]	2023	Aerobic exercise (*n* = 186), resistance exercise (*n* = 112), aerobic + resistance exercise (*n* = 740), resistance exercise + impact training (*n* = 77)	Usual care (*n* = 485), waitlist (*n* = 214), attention control group (*n* = 173)	Not provided	Small statistically significant effect on self-reported cognitive functioning (*β* = − 0.09, 95% CI − 0.16; − 0.02). Low baseline anxiety led to larger effects (*β* = − 0.10, 95% CI − 0.19; − 0.02) compared with participants with higher baseline anxiety (*β* = 0.07, 95% CI − 0.12; 0.26). In post-treatment exercise intervention studies (*n* = 745), a minimal, significant exercise effect on self-reported cognitive functioning in patients with cognitive problems at baseline is reported (*n* = 611, 82%) (*β* = − 0.19, 95% CI − 0.31; − 0.06)
Jesus, O.J. et al. [64]	2023	Moderate/ high intensity exercise (6)	Not provided	Not provided	In the intervention group, processing speed was the most influenced cognitive domain. Regarding the subjective cognitive function, there was no statistically significant difference between the groups. Significant effects on working memory were also reported, and three RCTs using objective tests observed moderate effects on working memory and episodic memory, or meaningful improvements in processing speed and spatial working memory
Mikkelsen, M.K. et al. [71]	2020	Individualised phone PA advice and booklet with exercises (1) Speed-feedback therapy on bicycle ergometers (1)	Not provided	Baseline pre and post week 4 (1), Pre, 1 year and follow-up at post 2 years (1)	The participants in the intervention group experienced a mean change from 15.00 (SE: 0.26) to 16.61 (SE: 0.22), whereas the control group showed a mean change from 14.50 (SE 0.30) to 14.95 (SE 0.36). These differences were significant *P*-value = 0.003 (1). One study did not find any significant differences between the groups concerning cognitive abilities as measured by verbal fluency
Myers, J.S. et al. [73]	2018	Aerobic exercise (3), resistance training (3), combination of aerobic and resistance (6), aerobic exercise and psychoeducation (1), yoga (5), tai chi (1), qigong (1), not reported (1)	Control (8), not included (7), other multiple groups (not controls) (3), relaxation control (2), waitlist (1)	Multiple variations at two or three assessment time points	Mixed results on improvements in subjective cognitive function from various combinations of exercise types and mindfulness. A mix of RCT (11) and non-RCT (15) studies reported improvements to cognitive function (7/11 RCT and 14/15 non-RCT). Others reported either mixed effects or no significant effect on cognitive outcomes
Persoon, S. et al. [78]	2013	Aerobic + strength (2), aerobic + ADLs (1), strength training (1), aerobic training (1)	Stretching (1), usual care (1), usual care + physical activity advice (2), relaxation (1)	Pre and post intervention	Cognitive functioning: sig effect favouring intervention: SMD: 0.36, 95% CI: 0.13–0.59;
Ren, X. et al. [80]	2022	Aerobic (6), yoga (2), qigong (1), aerobic + resistance (2), resistance (1)	Usual care (1), unspecified control (11)	Pre and post intervention	Self-reported cognition: significant improvement favouring intervention (MD: 10.12, 95% CI: 5.49–14.76); self-reported cognitive fatigue: significant improvement favouring intervention (MD: − 5.41, 95%: CI − 10.31 to − 0.51); processing speed: no significant difference between groups (MD: − 2.77, 95% CI: − 8.13–2.58); executive function: sig improvement favouring intervention (MD: − 13.63, 95% CI: − 21.86 to − 5.39); verbal memory: no sig difference between groups (MD: 0.58, 95% CI: − 2.34–3.50). Subgroup analysis suggested aerobic exercise could improve cognitive function, but body-mind exercise, resistance exercise, and combined exercise did not significantly improve cognitive function
Sharma, B. et al. [82]	2021	Aerobic and muscle building exercises and games (1), video game aerobic and balance exercises (1)	Wait list control (2)	Pre and post intervention	Attention: 1/1 study found no sig effect with intervention. Speed of processing: 1/2 studies found sig improvement with intervention; 1/2 studies found no sig effect with intervention. Verbal learning/fluency: 1/1 study found no sig effect with intervention
Yang, H.Y. et al. [95]	2023	Walking (5)	Usual care (3), Seated rest (2)	Pre and post intervention	Improvements in processing speeds (*P* = 0.03, *d* = 0.25) (*P* = 0.04, *d* = − 0.24) and MVPA intervention (*P* = 0.03, *d* = 0.65). Spatial working memory in a walking intervention (*P* = 0.03, *d* = − 0.12). Subjectively, 1/3 studies reported better cognitive function with between-group differences at borderline significance (*P* = 0.05, *d* = 0.94)
*Mind–body and psychological/behavioural*					
Cifu, G. et al. [53]	2018	Mindfulness based stress reduction (3), mindfulness-based stress reduction + other (3)	Usual care (3), waitlist (1), fatigue education and support (1), pre-post (1)	Post-intervention (6, 8, or 11 weeks) in all studies, and 2, 3, or 6-months post in five of the studies	Significant cognitive improvements post intervention and at 2 (η = 0.20) (ηp2 = 0.35), 3 (R2 = 0.234), and 6 (Cohen’s *d* = 0.55 month follow-up. 2 studies reported no significant effects on cognitive cluster scores (one study reported *P* = 0.052)
Myers, J.S. et al. [74]	2015	Mindfulness-based intervention (8), Mindfulness-based music therapy (1), Others (5)	Waitlist control (5), not included (5), supportive counselling (1), usual care (4)	Pre and post (9), Pre, post treatment, and follow-up (5), Pre and follow-up (1)	Meditation: Improvements for verbal memory, short-term memory, subjective cognitive function and processing speed. MBMT: Attention was improved at T2 (*P* = 0.022) as was mood (*P* < 0.001), decrease in fatigue (*P* < 0.001). Tai chi: Improvements in immediate and delayed memory, verbal fluency, attention, and executive functioning (*P* < 0.05) as well as self-reported verbal (*P* = 0.01) and visual memory (*P* < 0.05). Qigong: Self-reported cognitive improvements on both the EORTC items and FACT-COG (*P* = 0.05). Hypnotherapy: Improvements on EORTC cognitive items (OR = 5.18, reliability interval 1.07–23.02). Neurofeedback: Improvements on all self-reported cognitive measures (*P* < 0.001)
Hines, S. et al. [63]	2014	Cognitive behavioural therapy (6)	Usual care (1), unclear (5)	Not provided	Statistically significant effects on cognition reported in the short term. One study demonstrated significance at 20-week follow-up (*P* = 0.004) but was not sustained at 32-week follow-up. Another showed improvement between baseline and the completion of the intervention (*P* < 0.001), but no improvement at 6-month follow-up (*P* = 0.09)
Zhang, Y. et al. [98]	2017	Meditation (2), qigong (1), yoga (1)	Unspecified control (4)	Pre and post (4)	Subjective cognitive function: Any intervention (meditative/relaxation-based): MD: 5.29 (favouring intervention), 95% CI: 2.97–7.61; qigong or yoga: MD: 7.50, 95% CI: − 3.95–18.94. Objective cognitive function: Meditation: MD: 0.18, 95% CI: − 0.11–0.48
*Multi-modal/complex*					
Zhao, G. et al. [99]	2021	Multimodal Health Education/ Training (5)	Usual care (5)	Pre and post intervention (5)	Cognitive function score: MD: 6.65, 95% CI: 5.59–7.71
Zhao, K. et al. [100]	2020	Multimodal Rehabilitation (5)	Not Provided	Pre and post intervention (5)	Cognitive functional independence measure: SMD: 0.35, 95% CI: 0.19–0.50, *I*^2^ = 0%, *P* < 0.05 (exact p not provided)
Wang, X. et al. [92]	2015	Comprehensive nursing care (3)	Not Provided	Pre and post intervention	Cognitive function: sig effect with intervention (SMD = 0.64, 95%CI: 0.44–0.83)
Cheng, X. et al. [52]	2018	Nurse led interventions (7)	Usual care (7)	Multiple variations at two, three, or four assessment time points	The combined results implied that the nurse-led management strategy had no impact on the cognitive function of the participants (SMD = 0.07, 95% CI = − 0.28 to 0.42; *P* = 0.700)
*Mixed*					
Binarelli, G. et al. [1]	2023	Cognitive training (20), physical activity (11), mind–body (11), and multi-modal (4)	Waitlist (15), control group (19), no comparison (5), usual care (6), music listening (1)	Multiple variations from post-intervention to 24 months	Cognitive interventions: Improvements in subjective and objective CRCI i.e. episodic memory, working memory, executive function, attention, neural connectivity, and processing speed. Physical activity: High levels associated with fewer cognitive complaints and improved subjective cognitive function. Mind–body: Effects of meditation and mindfulness on cognition are inconclusive. Meditative-movement therapies decreased cognitive complaints. Multimodal interventions: One study combining physical activity and cognitive interventions decreased cognitive complaints, another showed no benefits on cognitive health
Binarelli, G. et al. [45]	2021	Cognitive simulation (16), computerized physical activity (4)	Waitlist (10), control group (7), no comparison (2)	Multiple variations at two or three assessment time points	Computerized cognitive simulation: Improvement in objective cognitive domains (especially memory, attention, processing speed, and executive functions), cognitive complaints, mental fatigue, planning and task monitoring, learning problems. Computerised physical activity: Improvement in attention, information processing speed, verbal memory, and executive functions. One study reported significant improvement in cognitive complaints. In one study, beneficial effects were maintained at the 6-month follow-up
Chan, R.J. et al. [50]	2015	Cognitive behavioural therapy (4), mindfulness (1), physical activity (2)	Control (1), psychoeducation (1), waitlist (3), usual care (2)	Not provided	Improvements reported in memory, subjective cognition, executive function in various studies. Other studies in the review reported no significant improvements in cognitive function or memory dysfunction
Cheng, A.S.K. et al. [51]	2022	Virtual reality (1), cognitive training/rehab (14), meditation (4), psychoeducation and cognitive exercises (1), CBT (3), yoga (1), psychoeducation (1), mixed (1), psychological nursing intervention (1)	Waitlist (9), supportive therapy (2), music listening (1), normative sample (1), combinations of cognitive rehab and aerobic exercise (1), not reported/unclear (13),	Not provided	The result of global inconsistency levels in subjective and objective cognition had no statistical significance (Chi square = 1.43, *P* = 0.69 and Chi square = 2.42, *P* = 0.49). The ranking probability of interventions for subjective and objective cognition is as follows: Cognitive training (CT) to manage objective cognition of memory and verbal fluency; cognitive rehabilitation (CR) for managing objective cognition of executive function; and supportive care for improving information processing speed
Drijver, A.J. et al. [54]	2022	Self-care + self-hypnosis (1), yoga (1), acupuncture vs. CBT (1), CBT (1) progressive music relaxation (1)	Waitlist (1), control (2), CBT (1), behavioural placebo treatment (1)	Not Provided	A small near-significant improvement reported in attentional function compared to the control group (*d* = 0.56, *P* = 0.07). A different study comparing CBT-I versus acupuncture found significant improvements on verbal learning (Cohen’s *d* = 0.25–0.50); the effects were sustained at 8 and 20 weeks (Cohen’s *d* = 0.54–0.84). Self-care + self-hypnosis (Cohen’s *d* = − 0.43, *P* = 0.02) and yoga (*P* < 0.05) decreased self-reported cognitive problems. Post-intervention (progressive muscle relaxation vs. autogenic training vs. control) scores of self-reported cognitive problems decreased compared to baseline scores for all groups (*F* = 14.1, *P* < 0.001)
Egset, K.S. et al. [56]	2024	Cognitive rehabilitation/training (12), neurofeedback (1), physical activity (2), active video-gaming (1), problem-solving therapy (1)	Waitlist (7), placebo (4), no comparison (6)	3 months (5), 6–12 months (7), no follow-up (5)	Mixed interventions reported improvements in attention, visual and verbal memory, executive performance from baseline to post-intervention and (in one study) up to 6-month follow-up. Some included studies did not find improvements in attention, memory, cognitive rehabilitation
Floyd, R. et al. [58]	2021	Cognitive training (6), exercise (2), acupuncture (1), Tibetan sound meditation (1)	Not Provided	Not Provided	Cognitive training: improvements in subjective cognitive function (*d* = 0.52, *P* = 0.02), subjective memory (*d* = 0.65, 95% CI 0.11, 1.19, *P* = 0.021), objective immediate memory (*d* = 0.82, 95% CI 0.28, 1.35, *P* = 0.004), delayed memory (*d* = 0.72, 95% CI 0.18, 1.26, *P* = 0.010), processing speed (*d* = 0.67, 95% CI 1.21, 0.14, *P* = 0.016), attention (*P* = 0.026), immediate (p ≤ 0.001) and delayed recall (0.008), memory (p ≤ 0.001), and executive function (*P* = 0.001). Computerised cognitive training: improved subjective (*d* = 0.19–0.38, *P* < 0.05) and objective cognition, particularly WCST (*d* = 0.74, *P* = 0.008), letter fluency (*d* = 0.39, *P* = 0.003), and symbol-search (*d* = 1.0, *P* = 0.009). Another online program reported no significant results on cognition. Aerobic exercise: improved subjective cognition and memory (*P* < 0.05). Other: Acupuncture study found improvements in subjective cognition and memory (objective); Tibetan sound meditation study improved memory (objective)
He, K. et al. [61]	2023	In-person sessions + tele-rehabilitation (1), neurofeedback (1), physical activity (2)	Usual care (3), placebo (1)	Not provided	Cognitive function was assessed in 4 studies. The results were statistically significant (pooled SMD = 0.863, 95% CI = 0.266– 1.460, *P* = 0.05)
Kasteler, R. et al. [65]	2023	Physical activity (3), online cognitive training (5), behavioural interventions (4)	No training group (3), placebo feedback (1), telephone psychological consultation (1), generic cognitive training (1), usual care (1), pre-post (5)	Multiple variations at two, three, or four assessment time points	Exercise interventions: Improvements in response accuracy, reaction time, short-term memory tasks, brain changes associated with improved metric measures and reaction time. Online cognitive training: Improvements in verbal and visual working memory tasks, academic achievement, working memory (not reported in longer term), and visual processing. Online neurofeedback found no effect on attention, processing speed, memory, executive functioning, visuomotor integration, and intelligence. Behavioural interventions: Reportedly helped slow down activation and improved motor and processing skills, however, they did not improve cognitive tests, executive, or intellectual functioning. Traditional CBT sessions reduced attention problems
Kirkman, M.A. et al. [67]	2022	Cognitive rehabilitation (1), diet (1), goal management training (1)	Standard of care/diet (2), waitlist (1)	Multiple variations at three or four assessment time points	Mixed results with some data presenting improvement to cognitive function with no statistical analysis, other data reported a non-significant increase in attention scores (12% in intervention group, *P* = 0.187 and 23.5% in the control group, *P* = 0.103), increasing until 1 month follow-up. Goal management training reported a significant intervention effect on the executive composite at four months follow-up, and moderate gains in processing speed. Both the Goal Management Training and Brain Health Program groups reported fewer cognitive concerns immediately post-training (*P* = 0.049) and at 4-month follow-up (*P* < 0.001)
Kirkman, M.A. et al. [68]	2023	Cognitive rehabilitation (11), cognitive training (6), physical activity (1), hyperbaric oxygen therapy (2), ketogenic diet and intermittent fasting (1)	Usual care (3), Waitlist (3), delayed treatment (1), active control (4), standard diet (1), healthy controls (2), stroke patients (2), not provided (5)	Multiple variations at two, or three assessment time points	Cognitive training: Significant increases on Digit Span (ηp2 = 0.35, *P* = 0.01) and Symbol Span (ηp2 = 0.25, *P* = 0.04), verbal learning (*P* = 0.04), word recall (F(1,22) = 109.75, *P* < 0.001), semantic clustering (F(1,22) = 87.89, *P* < 0.001), and executive composite (time-by-group interaction: F(2,16) = 3.760, *P* = 0.046, ƞp2 = 0.320). Cognitive rehabilitation: Significant improvements in global cognition (*P* < 0.05), attention (*P* = 0.004) and verbal memory (*P* = 0.009; effect sizes (d) ranged from 0.23 to 0.55). Some studies found no significant difference in the group over time. Physical activity: Improvements in attention, executive function, information processing speed and verbal memory. Hyperbaric oxygen therapy: Visual-sensorimotor integrity and sustained attention were significantly improved in one study. Diet: Attention scores increased significantly, yet not significantly different by diet types. Virtual reality: Improvement in concentration, verbal memory, spatial memory, and visual-motor coordination
Liu, Y. et al. [70]	2023	Walking (1), cognitive training (1) acupuncture therapy (2), memory and attention adaptation training (2), CALM (1), yoga and mindfulness (5)	Placebo, usual care, no intervention, waitlist control, supportive therapy or other nonpharmacologic intervention	Follow-up between 1 to 3 months, 5 studies had no follow-up	There were no significant differences in subjective outcomes when compared to the control group. However, for objective outcomes, both qigong (SMD, 1.27; 95% CI, 0.13–2.41) and exercise (SMD, 1.13; 95% CI, 0.10–2.16) showed significant improvements compared to psychotherapy. Similarly, qigong (SMD, 1.69; 95% CI, 0.10–3.28) and exercise (SMD, 1.55; 95% CI, 0.03–3.06) were also significantly more effective than music therapy. No statistically significant differences were found when compared to other nonpharmacologic interventions
Mackenzie, L. et al. [19]	2022	Cognitive training (15), cognitive behavioural therapy (4), physical activity (15), supportive therapies (11)	Usual care (22), waitlist (12), education/support controls (6) no contact (1), physical training (1), Sham Qigong (1), relaxation group (2), usual care + dietary guidelines (1)	Multiple variations with assessment time points from 4 weeks to 9 months	Cognitive training interventions: Standardized mean difference of 0.407 (95% CI: 0.283–0.531), *P* = 0.001. CBT: Mixed effect on cognitive performance; standardized mean difference of 0.297 (95% CI: 0.137–0.456), *P* = 0.001. Physical activity interventions: Standardized mean difference of 0.272 (95% CI: 0.199–0.346), *P* = 0.001, favouring the interventions overall. Supportive therapies: Standardized mean difference of 0.274 (95% CI: 0.161–0.387), *P* = 0.001, favouring the interventions overall
Morean, D.F. et al. [72]	2015	Physical strength-training (1), restorative therapies (2), cognitive therapies (5)	Not provided	Baseline and post treatment (8), follow-up between 1- and −6-month post-treatments (7)	Improvements in attention (*P* = 0.01) and (*P* = 0.019), verbal memory (*P* = 0.048); using cognitive memory and attention adaptation training *P* < 0.001 to *P* < 0.05 for processing speed and verbal memory; computerized EF training for processing speed (*P* = 0.009)
Oh, P.J. et al. [75]	2016	Cognitive training/psychological (11), behavioural (3)	Control (3), Wait list (6), Standard care (2), Usual care waitlist (1), Usual care (2)	One follow-up assessment after the intervention (6), Not clear/ reported (8)	Significant treatment effects on memory; the weighted average effect size (n = 8) was 0.21 (95% confidence interval [CI] [0.04, 0.38], *P* = 0.02). A significant small effect on subjective cognitive functioning (*d* = 0.41, 95% CI [0.2, 0.61], *P* < 0.001). No effects were noted on cognitive performance as measured by tests of attention, executive functioning, and verbal ability. For subgroup analysis, a significant effect on subjective cognitive function (n = 4) was observed (*d* = 0.35, 95% CI [0.13, 0.58], *P* = 0.002). No significant effect of behavioural interventions on cognitive functioning was observed (*P* = 0.05)
Oldacres, L. et al. [76]	2023	Psychoeducational/psychosocial (7), cognitive behavioural therapy/compensatory strategies (6), integrative/complementary (4), brain‐training (5), exercise (4)	Usual care (4), Control (11), Waitlist (6), Supportive therapy (1) Not included (3), Multisensory stimulation group (1), Minimal Acupuncture (1)	Pre and post (26)	Psychoeducational and psychosocial: significant improvement in executive function, language, memory, and subjective measures. Cognitive behavioural therapy/compensatory strategies: significant improvement in attention & concentration, language, memory, and subjective measures. Integrative/complementary: sig improvement in attention & concentration, executive function, language, memory, subjective measures. Brain Training: significant improvement in attention & concentration, executive function, language, memory, subjective measures. Exercise: Significant improvement in attention & concentration, executive function, memory, subjective measures
Park J.H. et al. [77]	2023	Cognitive rehabilitation (8), CBT (6), Exercise (7)	Waitlist control (10), usual care (11), survivorship support (1), supportive therapy (1)	Pre and post	Attention: Significant effect favouring intervention: *g* = 0.83; 95% CI: 0.14 to 1.52, Verbal memory (immediate): Sig effect favouring intervention: g = 0.33; 95% CI: 0.18 to 0.49, Verbal memory (delayed): No significant difference between groups: g = 0.08; 95% CI: 0.11 to 0.26, Executive function: Significant effect favouring intervention: g = 0.25; 95% CI: 0.13 to 0.37, Processing speed: Significant effect favouring intervention: g = 0.44; 95% CI: 0.14 to 0.73, Language fluency: Significant effect favouring intervention: g = 0.23; 95% CI: 0.01 to 0.47. Subjective cognitive function: Significant effect favouring intervention: *g* = 0.68; 95% CI: 0.40 to 0.96
Simone, A. et al. [83]	2023	Aerobic exercise (2), computer training program Brain HQ (1), group memory training (1)	Pre-intervention data (1), usual care (2), education only (1)	Pre and post (1) and 1- to 14-month follow-up (3)	Global cognition: no significant improvement with intervention (*n* = 1/1 study); Perceptual motor: no significant improvement with intervention (*n* = 1/1 study); Memory: significant improvement with intervention (*n* = 2/2 studies); Attention: significant improvement with intervention (*n* = 1/1 study); Executive function: significant improvement with intervention (*n* = 1/2 studies), no sig improvement with intervention (*n* = 1/2 studies)
Singh, N. et al. [84]	2022	Exercise (6), exercise, combined with other (2), cognitive training (2)	Waitlist control (1) usual care (1), active control (2), education (3), unspecified (3)	Pre and post	Improvements in cognitive problems, cognitive function, processing speed, memory, executive function, self-efficacy, attention
Treanor, C.J. et al. [85]	2016	Cognitive training (3), exercise (1), mindfulness-based (1)	Delayed exercise (1), waitlist control (4)	Pre and post (2) and 1-month follow-up (1) or 2-month follow-up (2)	Computerised cognitive training: Statistically significant improvements in processing speed, executive functions, cognitive flexibility, language, memory, self-reported global cognitive function, planning and/or organising, task monitoring. Compensatory strategy training: Statistically significant improvements in memory and self-reported cognitive function, no significant difference in processing speed. Meditation: No significant difference in processing speed or memory. Physical activity: Statistically significant improvements in processing speed, executive functions, verbal fluency
Van Lonkhuizen, P. J. C. et al. [86]	2019	Exercise (2), cognitive strategy training (5), cognitive re-training (2), combined cognitive strategy and re-training (3)	Pre-intervention data (5), waitlist control (2), active control (4), unspecified control (1)	Pre and post (5) and < 6 months follow-up (2) or > 6 months to 1 year follow-up (5)	Cognitive strategy training: Statistically significant improvements in memory, executive function, processing speed. Cognitive re-training: Statistically significant improvements in attention, memory, verbal fluency, and visual-motor coordination. Combined strategy and re-training: Statistically significant improvements in subjective cognitive function, attention, memory, mental fatigue. Exercise (*n* = 2 studies): Improvements in MMSE score and verbal learning
Vance, D.E. et al. [87]	2017	Cognitive training (2), compensatory strategies with cognitive training interventions (5), exercise (6), mindfulness-based (1), other (1)	Pre-intervention data (5), waitlist control (7), usual care (3)	Pre and post (3) and 1- to 6-month follow-up (12)	Cognitive training: Statistically significant improvements in memory, attention, speed of processing, strategy formation, set shifting, verbal fluency, timed visual search and matching, self-reported executive function. Compensatory strategies with cognitive training: Statistically significant improvements in self-reported cognitive function, speed of processing, self-reported executive function, memory. One study reported no effect in self-reported cognitive function. Exercise: Statistically significant improvements in self-reported cognitive function, memory, verbal fluency, executive function, and confusion. Meditation: Statistically significant improvements in memory, no significant effect on cognitive deficits or processing speed. Biofeedback: Statistically significant improvements in subjective cognitive functioning
Vannorsdall, T.D. et al. [88]	2021	Exercise (7), psychotherapy (2), alternative therapies (2)	Usual care (5), waitlist (1), muscle relaxation (1), dim red light (1), education (1), sham (1)	Pre and post (10) and 6-week follow-up (1)	Cognition: Significant effect favouring intervention: Hedges’ *g* = − 0.38; 95% CI − 0.65 to − 0.11; *P* < 0.01
Von Ah, D. et al. [90]	2014	Cognitive training (16), biofeedback (1), exercise (4), mindfulness-based (2), cognitive rehabilitation (1), natural restorative environment (2)	Not provided	Not provided	Cognitive training: Statistically significant improvements in cognitive function (1/7 study reported no effect). Cognitive behavioural training: Statistically significant improvements in objective and subjective cognitive function (5/9 reported no effect on objective cognitive function). Biofeedback: Statistically significant improvements in subjective cognitive function. Exercise: no established effectiveness. Tibetan sound meditation: No significant effect of memory, speed of processing, subjective cognitive function. Mindfulness based stress reduction: Improvement in confusion (no *P*-value provided). Natural restorative environment interventions: Improvement in attention (no *P*-value provided). Qigong and physical and psychosocial rehabilitation: Statistically significant improvements in subjective cognitive function
Zeng, Y. et al. [97]	2020	Cognitive training/rehabilitation (11), Aerobic exercise (5), CBT (3), tai chi/qigong (3), yoga/meditation/ mindfulness (4), cognitive training + exercise (1), acupuncture (1), supportive therapy (1)	Usual care (2), waitlist (23), supportive therapy (2), active control (1), Sham Qigong (1)	Pre and post (29)	The overall effects favoured the four non-pharmacological interventions of cognitive training, cognitive rehabilitation, exercise, and meditation versus usual care/wait-list control. Only 4 interventions reported statistically significant improvements. Relative effect size of meditation/mindfulness-based stress reduction, cognitive training, cognitive rehabilitation, and exercise intervention were 10.26 (1.53, 19.00), 5.02 (1.41, 8.63), 4.88 (0.65, 9.11), and 3.82 (0.52, 7.13), respectively
Zimmer, P. et al. [101]	2016	Animals: running wheel (5); humans: physical activity (5), yoga/meditation (3), qigong (1), tai chi (1)	Animals: unspecified control (5) Humans: Unspecified control (7), pre intervention data (3)	Pre and post (15)	Animals: Improvements in memory, non-matching to sample task, novel object recognition, Morris water maze, Barnes maze and open-field test (no effect reported for open-field test in 1/2 studies). Humans: Improvements in self-reported cognitive function, memory, frontal assessment battery, attention, concentration. No effect reported in self-reported cognitive function for 1/4 studies, attention in 1/2 studies, memory in 2/4 studies or executive function in 1/1 study

*Data for all studies included in the review are displayed, as relevant studies only could not be parsed separately. + Data only reported for the *n* = 71 experimental studies, therefore data from the other 4 included studies are not reported in this table. *SMD*, standardised mean difference; *WMD*, weighted mean difference; *CT*, cognitive training; *RCT*, randomised control trial; *QoL*, quality of life; SE, standard error; *MVPA*, multivariate pattern analysis; *EORTC*, European Organisation for Research and Treatment of Cancer; *CBT*, cognitive behavioural therapy

#### Cognitive training/rehabilitation

In total, 15 (23%) SRs primarily assessed cognitive training/rehabilitation interventions, with a further 21 (33%) including some cognitive training/rehabilitation interventions. All SRs primarily assessing cognitive training/rehabilitation included RCTs (*n* = 15) with 27% of these only including RCTs. Interventions reported included computerised cognitive training (13%), multi-component cognitive rehabilitation (13%), strategy training (7%), and a combination of these interventions subjective measures, such as the Functional Assessment of Cognitive Therapy-Cognitive Function (FACT-COG) and the Patient Reported Outcomes Measurement Information System Cognitive Function (PROMIS-COG), and objective measures such as the Wisconsin Card Sorting Task, Trail Making Test, and the Auditory Verbal Learning Test (see Supplementary Materials, Part 3 for a full list of measures in each SR).

Interventions typically resulted in improvements in cognition in cancer survivors. Included meta-analyses primarily assessing cognitive training/rehabilitation found efficacy in improving subjective cognition (n = 2; SMD range = 0.30–0.52), and objective cognition (Memory (n = 3); SMD range = 0.18–0.42, median = 0.40. Attention (*n* = 2) SMD range = 0.09–0.81. Executive Functioning; SMD range = 0.15–0.81), with primarily medium effects. Overall, 87% of the SRs primarily assessing cognitive training/rehabilitation reported general efficacy (> 50% primary studies showing efficacy) in improving > 50% of measured domains of subjective and/or objective cognitive functioning. For example, Von Ah et al. [89] found cognitive training and rehabilitation resulted in pre- to post-intervention improvements in memory, self-reported cognitive functions, attention, verbal fluency, processing speed, letter fluency, cognitive flexibility, executive function, and global cognition for adult cancer survivors of various tumour types. Yan et al. [94] found cognitive training significantly improved processing speed, verbal memory, working memory, episodic memory, and overall subjective cognition in mid-to-older adults across various cancer types and stages. However, there was some variability in results with SRs finding some cognitive training/rehabilitation interventions to be ineffective in improving particular domains of cognition in cancer survivors. All (*n* = 34) SRs that assessed cognitive training/rehabilitation interventions found some interventions to be ineffective for one or more cognitive processes. For example, while Von Ah et al. [89] typically found improvements in cognition, other studies reported no improvements in memory, subjective cognitive function, executive function, processing speed, psychomotor speed, attention, verbal fluency, subjective cognitive function, cognitive flexibility, daily function, or global cognition. Similarly, Yan et al. [94], while finding general effectiveness in their meta-analysis, found no significant improvements from cognitive training on the domains of attention, short-term memory, or executive functioning.

While many of the reviews discussed or attempted to identify differentiating interventional, methodological, or participant characteristics explaining the variability in effectiveness observed, no clear and consistent components, factors, or characteristics could explain intervention effectiveness variation across the SRs. Overall, cognitive training/rehabilitation interventions were found to be effective for improving cognition in cancer survivors across various tumour types, stages, and participant ages, however, there was variability in effectiveness across a range of cognitive processes with no clear differentiating components, factors, or characteristics.

#### Physical activity/exercise

Overall, 15 (23%) SRs primarily assessed physical activity/exercise interventions, with a further 20 (31.3%) including physical activity/exercise interventions. Fifteen SRs included some RCTs on physical activity/exercise, and ten focused exclusively on RCTs. The majority of SRs (*n* = 12; 80%) included a mix of aerobic, resistance, and combined aerobic/resistance interventions. A further three SRs (20%) included only aerobic interventions (e.g. walking, cycling, jogging). Resistance training included weighted exercise programs (see Supplementary Materials, Part 3 for additional details of interventions). Cognitive measures included both subjective measures, such as the European Organisation for Research and Treatment of Cancer-Cognitive Functioning Scale (EORTC-CFS), PROMIS-COG, and the Cognitive Failures Questionnaire, and objective measures such as CogState, Montreal Cognitive Assessment (MOCA), and Trail Making Test (see Supplementary Materials, Part 3 for a full list of measures in each SR).

The interventions typically resulted in improvements in cognition in cancer survivors, although mixed results were common. Included meta-analyses primarily assessing physical activity/exercise interventions found slight efficacy in improving subjective cognition (*n* = 7, SMD range = 0.01–0.51, median = 0.18); objective cognition (memory (*n* = 1, SMD = 0.03); executive functioning (*n* = 1, SMD = 0.19); processing speed (*n* = 1, SMD = 0.08 and overall (*N* = 1; paediatric/adolescence, SMD = 0.40) with primarily small to medium effects. Overall, 60% of the SRs primarily assessing physical activity/exercise interventions found general efficacy (> 50% primary studies showing efficacy) in improving > 50% of measured domains of subjective and/or objective cognitive functioning. For example, Bernal et al. [44] found a small-to-medium effect of an exercise intervention on childhood cancer survivors (various types) in objective (5 RCTs) and subjective (13 RCTs) cognitive functioning. Furthermore, Hiensch et al. [62] found resistance, aerobic, and mixed exercise interventions to have a small statistically significant effect on self-reported cognitive functioning in mid-to-older adult survivors of various cancers. There was notable variability in results with some SRs finding some physical activity/exercise interventions to be ineffective in improving cognition in cancer survivors across general cognition or within specific cognitive domains. For example, Brunet et al. [46] found that, across a wide range of physical activity and exercise interventions, the effects were inconclusive in 71% (*n* = 50) of the included primary studies, and null effects were reported for subjective cognition.

Across the SRs, there was no clear pattern between physical activity and exercise intervention types and dose, participant characteristics, and efficacy of the interventions on particular or general subjective or objective cognitive performance. However, Bernal et al. [44] found studies that involved participants with a longer time since cancer diagnosis, or end of cancer treatment, more female participants, and lower levels of supervision were significantly associated with smaller improvements in cognitive function. Additionally, primary study quality may be related to efficacy. For example, Baydoun et al.’s [42] SR on the effects of yoga on cognition, found studies rated as ‘high quality’ reported positive effects while those rated as lower quality found null effects.

Overall, the slight majority of physical activity/exercise interventions were found to be effective in improving cognition in cancer survivors across cancer types and stages, and across physical activity/exercise types. However, there was a significant variability in the findings and the majority of positive effects were small to medium.

#### Mind–body and psychological/behavioural

Four (6%) SRs primarily assessed mind–body and psychological/behavioural therapy interventions, with a further 18 (28%) including some mind–body and psychological/behavioural therapy interventions. Four SRs primarily assessing mind–body and psychological/behavioural therapy interventions included non-RCTs and 25% (*n* = 1) included exclusively RCTs. These SRs included the interventions of mindfulness/stress reduction-based interventions (*n* = 2; 50%), and cognitive behavioural therapy (*n* = 1; 25%) (see Supplementary Materials, Part 3 for additional detail of interventions). Cognitive measures included both subjective measures, such as the Multiple Ability Self-Report Questionnaire, and the EORTC-CFS, and objective measures such as the California Learning Verbal Learning Test, and Stroop Task (see Supplementary Materials, Part 3 for a full list of measures).

The interventions typically resulted in improvements in cognition in cancer survivors. The included meta-analysis primarily mind–body and psychological/behavioural therapy found efficacy in improving subjective cognition (*n* = 1; SMD = 0.26), and objective cognition (Overall (*n* = 1); SMD = 0.11) with a small to medium effect. Overall, 80% of the SRs specifically assessing mind–body and psychological/behavioural therapy found general efficacy (> 50% primary studies showing efficacy) in improving > 50% of measured domains of subjective and/or objective cognitive functioning in cancer survivors. For example, Cifu et al. [53] found mindfulness-based interventions were typically effective at improving objectively and subjectively measured cognition in mid-to-older age adults with breast cancer from pre-intervention to the final 6-month follow-up measures. Furthermore, Hines et al. [63] in their SR on the effectiveness of CBT in improving cognition in cancer, found CBT to be typically effective for improving objective and subjectively measured cognition in cancer survivors who have had chemotherapy in the short-term post-intervention assessments. However, there was some variability in results, with all these SRs reporting some null findings. For example, while Hines et al. [63] found general efficacy of CBT intervention for improving short-term objective and subjective cognition, the majority of included primary studies did not demonstrate longer-lasting effects, finding no significant positive impacts of the interventions from 6 to 32 weeks. Furthermore, Zhang et al. [98] in their SR assessing mindfulness/relaxation-based interventions in adult cancer survivors found significant effects on subjective, but not objective measures of cognition.

Overall, mind–body and psychological/behavioural therapy interventions were found to be largely effective in improving cognition in cancer survivors. However, there was significant variability in the improvement of subjective or objective cognition across the SRs with no clear methodological or participant characteristics to explain this variability. Furthermore, the efficacy of mind–body and psychological/behavioural therapy interventions in improving cognition is primarily in the short-to-midterm (i.e. up to 6 months), with questionable efficacy longer-term.

#### Multi-modal/complex interventions

Four (6.3%) SRs assessed multi-modal/complex interventions. Two SRs only included RCTs (*n* = 2; 50%). Multi-modal/complex interventions included model of care approaches (*n* = 3; 75%), and health education (*n* = 1; 25%) (see Supplementary Materials, Part 3 for additional detail of interventions). Cognitive measures included both subjective measures, such as EORTC-CFS, and objective measures such as the Cognitive Functional Independence Measure) (see Supplementary Materials, Part 3 for a full list of measures in each SR).

Overall, the interventions typically resulted in improvements in cognition in cancer survivors. Most (75%) of the SRs assessing multi-modal/complex interventions found general efficacy (> 50% primary studies showing efficacy) in improving > 50% of measured domains of subjective and/or objective cognitive functioning in cancer survivors. For example, Zhao et al. [99] in their SR of health education RCTs in gastrointestinal cancer survivors found health interventions were effective in improving subjective cognition (no objective assessments were included) with a modest effect. Similarly, Wang et al. [92] in their SR of comprehensive nursing care interventions for lung cancer survivors found the interventions improved self-reported cognition with a medium effect. However, given the broad inclusion criteria of these multi-modal/complex intervention SRs, the specific efficacious mechanisms of effect are unknown. This also leads to challenges in understanding the factors that may result in an efficacious model of care intervention versus an intervention with null effects. For example, Cheng et al. [51] in their SR assessing multiple types of nurse-led interventions across cancer types found no effect of nurse-led interventions on subjective cognition. However, the characteristics of the interventions which may have led to a null effect are unclear.

Most (75%) multi-modal/complex interventions were found to be effective in improving cognition in cancer survivors. However, given the broad inclusion criteria of these SRs, it is challenging to understand the mechanisms of effect.

## Discussion

The aim of this research was to provide a comprehensive overview of findings from systematic reviews examining the effectiveness of non-pharmacological interventions in improving cognition in cancer populations. This review is timely, given the inclusion of cognition as a key rehabilitation domain within the WHO Package for Rehabilitation from Cancer [102], and the increased recognition of CRCI as a condition that has significant and potentially long-term impacts on the lives of cancer survivors across multiple domains [4]. Our findings show support for the efficacy of non-pharmacological interventions for improving cognition in cancer survivors. These findings provide support for the exciting potential for these non-invasive, non-substance-based approaches, which have minimal risk for negative side effects, to be integrated into supportive care practice.

The majority of the literature focused on cognitive training/rehabilitation and physical activity/exercise interventions for the improvement of cognition in cancer. There were relatively fewer reviews focused on mind–body, psychological/behavioural interventions, and multi-modal/complex interventions. Cognitive training/rehabilitation interventions demonstrate the strongest support for improving cognition in cancer across SRs. Cognitive training and rehabilitation interventions have been the primary focus of non-pharmacological interventions in cancer [94] and have the most extensive supporting literature across intervention types. However, drawing strong conclusions regarding the efficacy of particular mechanisms of cognitive training/rehabilitation for sub-groups of cancer survivors remains a challenge [86]. This is due to the variability in how cognitive domains are defined, the approaches used, and comparators selected, as well as small sample sizes which impact sub-group analyses and analyses using more sophisticated statistical approaches [86, 103]. This heterogeneity means, while this review can support the general efficacy of cognitive training/rehabilitation for improving cognition in cancer, nuances regarding approaches, dose, frequency, cognitive processes, and survivor clinical and personal characteristics cannot be determined. Recent findings indicate that strategy training and combination cognitive rehabilitation are promising, and speed of processing may be the most challenging cognitive process to improve via these approaches [104]. However, nuance beyond these trends cannot be currently established. This suggests that cognitive training/remediation interventions in cancer should adopt harmonisation guidelines such as the International Cognition and Cancer Task Force recommendations to harmonise studies of cognitive function in patients with cancer [105]. Furthermore, the inconsistency in definitions of cognitive domains suggests definition guidelines should be developed for CRCI research that explicitly define each cognitive domain.

Physical activity/exercise also shows promise for improving cognition in cancer survivors. We found general support for physical activity/exercise in improving cognition in cancer with some variability. Similar to cognitive training/rehabilitation, there was no clear pattern between physical activity/exercise intervention (e.g. resistance training, aerobic, combination, type, dose, frequency) or participant characteristics that differentiated efficacious and non-efficacious findings. There was also no clear intervention or participant characteristics that could differentiate between effectiveness on any particular cognitive domain. The challenges of understanding the heterogeneity of physical activity/exercise effects on particular health and well-being outcomes are widely acknowledged, beyond cancer [106]. In recent years, there has been a movement to gain a greater understanding of physical activity/exercise response heterogeneity across populations using physiological, molecular, and health history data [107]. Given the notable physical activity/exercise response variation across different outcomes, more fine-grained, individual-level assessments of the effects of exercise/physical activity on cognition in cancer may be usefully explored or examined.

Few SRs primarily assessed mind–body and psychological/behavioural therapy interventions. However, this overview found general efficacy for mind–body and psychological/behavioural therapy interventions in improving cognition, which has gained recognition as a potential key nonpharmacological intervention for CRCI [108], although, the primary literature is still lacking. This overview suggests that non-going (i.e. 12 weeks) mind–body and psychological/behavioural therapy interventions may primarily be effective for the short-term post-intervention (i.e. up to 6 months). This is perhaps unsurprising as mind–body and psychological/behavioural therapy interventions are ideally applied ongoing and/ or long-term, beyond a 12-week intervention period (i.e. the longest follow-up found in this review). For example, cognitive behavioural therapy often demonstrates improved efficacy on psychological outcomes over time as practice becomes embedded in routine functioning [109]. Future research should assess the longitudinal impacts of ongoing mind–body and psychological/behavioural therapy interventions. Furthermore, due to the lack of available literature, the specific effects of mind–body and psychological/behavioural therapy intervention type, dose, and frequency on particular cognitive domains remain unclear.

A small body of literature has assessed the broad efficacy of multi-modal/complex interventions on the improvement of cognition in cancer. These interventions were typically multi-component holistic care approaches aimed at improving cancer survivor outcomes and quality of life. While the results of this overview suggest these interventions may be generally effective, the SRs and interventions were broad and largely lacked definition and intervention clarity and specificity. Multicomponent nurse-led, and multidisciplinary rehabilitation interventions were the focus of the reviews, with the two nurse-led intervention SRs finding conflicting results. The lack of definition and intervention clarity and specificity reduces conclusions that can be drawn from the multi-modal/complex interventions. As shared, holistic, and nurse-led multi-modal/complex interventions have shown efficiency in improving cancer survivor outcomes generally [35, 110, 111], additional research in multi-modal/complex interventions to improve cognition in cancer survivors with a greater degree of specificity is required. Finally, there was a significant lack of reviews including dietary interventions for cognition in cancer. Given the positive impact dietary interventions may have on cognition in other populations [112], the research community should further explore the potential for dietary interventions for cognition in cancer.

### Limitations

There are three key limitations of this umbrella review: (1) as this review focused on developing a broad picture of the effectiveness of non-pharmacological intervention to improve cognition in cancer, it was beyond our scope to provide an in-depth assessment of intervention proposed mechanisms of action or components, nor differentiate in detail between different clinical and demographic characteristics (i.e. adolescents versus adults; non-CNS versus CNS cancers). This limits the ability to understand the nuances of an intervention's characteristics and rationale, as well as potential differential effects. (2) The overall quality of included SRs was low-to-moderate. While this is not a methodological limitation of our review, the results should be interpreted with caution. (3) Only two mixed intervention SRs provided any findings relating to diet and cognition in cancer. Thus, we were unable to narratively synthesise and provide overall findings on dietary interventions with confidence. Future research should examine dietary interventions for cognition in cancer.

### Recommendations

Considering the overall findings of this overview, we provide below key recommendations for future research in non-pharmacological interventions in cancer survivors.

1. Reporting guidelines for cognition in cancer, such as the International Cognition and Cancer Task Force recommendations should be adopted to harmonise studies of cognitive function in patients with cancer [105].
2. Standardised definitions for cognitive domains in CRCI research should be developed to increase consistency.
3. Future trials, in addition to trial effects, should investigate** i**f intervention and participant characteristics are associated with positive vs. null/negative findings for overall cognition and specific cognitive domains.

## Conclusion

This umbrella review highlights non-pharmacological interventions promise in improving CRCI. Cognitive training/rehabilitation currently has the greatest support for efficacy of any non-pharmacological approach. Physical activity/exercise showed promising efficacy; however, variability was significant. The literature on mind–body and psychological/behavioural therapy interventions was limited, but there is support for short-term effectiveness to improve cognition in cancer. Multi-modal/complex interventions are promising but typically poorly defined. Future research should focus on methodological, reporting, and definition consistency, and explore the relationship between interventional and participant factors and efficacy.

## Supplementary Information

Below is the link to the electronic supplementary material.

Supplementary file1 (PDF 536 KB)

## Data Availability

Data may be made available upon request of the corresponding author.
