# Supplementary material for: The effect of non-pharmacological interventions on cognitive function in cancer: an overview of systematic reviews
Source: Support Care Cancer. 2025 Feb 4;33(2):151. doi: 10.1007/s00520-025-09212-3 (PMC11794363; doi:10.1007/s00520-025-09212-3)
Supplement: Supplementary file 1 — Supplementary file1 (PDF 536 KB) [file 520_2025_9212_MOESM1_ESM.pdf]

THE EFFECT OF NON-PHARMACOLOGICAL INTERVENTIONS ON COGNITIVE FUNCTION IN CANCER: AN OVERVIEW OF SYSTEMATIC REVIEWS

Supplementary Materials

PART 1

“Gold Set” Of Articles for Text Mining

1. Baydoun, M., Oberoi, D., Flynn, M., Moran, C., McLennan, A., Piedalue, K. L., & Carlson, L. E. (2020). Effects of Yoga-Based Interventions on Cancer-Associated Cognitive Decline: a Systematic Review. *Curr Oncol Rep, 22*(10), 100. doi:10.1007/s11912-020-00960-5
2. Binarelli, G., Joly, F., Tron, L., Lefevre Arbogast, S., & Lange, M. (2021). Management of Cancer-Related Cognitive Impairment: A Systematic Review of Computerized Cognitive Stimulation and Computerized Physical Activity. *Cancers, 13*(20), 14. doi:https://dx.doi.org/10.3390/cancers13205161
3. Egset, K. S., Røkke, M. E., Reinfjell, T., Stubberud, J. E., & Weider, S. (2024). Cognitive and behavioural rehabilitation interventions for survivors of childhood cancer with neurocognitive sequelae: A systematic review. *Neuropsychol Rehabil*, 1-28. doi:10.1080/09602011.2024.2314880
4. Farahani, M. A., Soleimanpour, S., Mayo, S. J., Myers, J. S., Panesar, P., & Ameri, F. (2022). The effect of mind-body exercise on cognitive function in cancer survivors: A systematic review. *Canadian Oncology Nursing Journal, 32*(1), 38-48. doi:https://dx.doi.org/10.5737/236880763213848
5. Fernandes, H. A., Richard, N. M., & Edelstein, K. (2019). Cognitive rehabilitation for cancer-related cognitive dysfunction: a systematic review. *Support Care Cancer, 27*(9), 3253-3279. doi:10.1007/s00520-019-04866-2
6. Floyd, R., Dyer, A. H., & Kennelly, S. P. (2021). Non-pharmacological interventions for cognitive impairment in women with breast cancer post-chemotherapy: A systematic review. *J Geriatr Oncol, 12*(2), 173-181. doi:10.1016/j.jgo.2020.05.012
7. Hines, S., Ramis, M. A., Pike, S., & Chang, A. M. (2014). The effectiveness of psychosocial interventions for cognitive dysfunction in cancer patients who have received chemotherapy: a systematic review. *Worldviews Evid Based Nurs, 11*(3), 187-193. doi:10.1111/wvn.12042
8. Kim, Y., & Kang, S. J. (2019). Computerized programs for cancer survivors with cognitive problems: a systematic review. *J Cancer Surviv, 13*(6), 911-920. doi:10.1007/s11764-019-00807-4
9. Kirkman, M. A., Day, J., Gehring, K., Zienius, K., Grosshans, D., Taphoorn, M., . . . Brown, P. D. (2022). Interventions for preventing and ameliorating cognitive deficits in adults treated with cranial irradiation. *Cochrane Database Syst Rev, 11*(11), Cd011335. doi:10.1002/14651858.CD011335.pub3
10. Kirkman, M. A., Ekert, J. O., Hunn, B. H. M., Thomas, M. S. C., & Tolmie, A. K. (2023). A systematic review of cognitive interventions for adult patients with brain tumours. *Cancer Med, 12*(10), 11191-11210. doi:10.1002/cam4.5760
11. Kuil, L. E., Varkevisser, T., Huisman, M. H., Jansen, M., Bunt, J., Compter, A., . . . Partanen, M. (2024). Artificial and natural interventions for chemotherapy- and / or radiotherapy-induced cognitive impairment: A systematic review of animal studies. *Neurosci Biobehav Rev, 157*, 105514. doi:10.1016/j.neubiorev.2023.105514
12. Liu, Y., Liu, J. E., Chen, S., Zhao, F., Chen, L., & Li, R. (2023). Effectiveness of Nonpharmacologic Interventions for Chemotherapy-Related Cognitive Impairment in Breast Cancer Patients: A Systematic Review and Network Meta-analysis. *Cancer Nurs, 46*(5), E305-E319. doi:10.1097/NCC.0000000000001152
13. Mackenzie, L., & Marshall, K. (2022). Effective non-pharmacological interventions for cancer related cognitive impairment in adults (excluding central nervous system or head and neck cancer): systematic review and meta-analysis. *Eur J Phys Rehabil Med, 58*(2), 258-270. doi:10.23736/S1973-9087.21.06898-2

14. Morean, D. F., O'Dwyer, L., & Cherney, L. R. (2015). Therapies for Cognitive Deficits Associated With Chemotherapy for Breast Cancer: A Systematic Review of Objective Outcomes. *Arch Phys Med Rehabil, 96*(10), 1880-1897. doi:10.1016/j.apmr.2015.05.012
15. Oldacres, L., Hegarty, J., O'Regan, P., Murphy-Coakley, N. M., & Saab, M. M. (2023). Interventions promoting cognitive function in patients experiencing cancer related cognitive impairment: A systematic review. *Psychooncology, 32*(2), 214-228. doi:10.1002/pon.6073
16. Park, J.-H., Jung, S. J., Lee, L. J., Rhu, J., & Bae, S. H. (2023). Impact of nonpharmacological interventions on cognitive impairment in women with breast cancer: A systematic review and meta-analysis. *Asia-Pacific Journal of Oncology Nursing, 10*(4), 100212. doi:https://doi.org/10.1016/j.apjon.2023.100212
17. Yan, X., Wei, S., & Liu, Q. (2023). Effect of cognitive training on patients with breast cancer reporting cognitive changes: a systematic review and meta-analysis. *BMJ Open, 13*(1), e058088. doi:https://dx.doi.org/10.1136/bmjopen-2021-058088
18. Yang, H. Y., Chou, Y. J., & Shun, S. C. (2023). The Effect of Walking Intervention on Cognitive Function Among Patients With Non-Central Nervous System Cancer: A Systematic Review. *Cancer Nurs, 46*(5), 375-385. doi:10.1097/ncc.0000000000001106
19. Zeng, Y., Dong, J., Huang, M., Zhang, J. E., Zhang, X., Xie, M., & Wefel, J. S. (2020). Nonpharmacological interventions for cancer-related cognitive impairment in adult cancer patients: A network meta-analysis. *Int J Nurs Stud, 104*, 103514. doi:10.1016/j.ijnurstu.2019.103514
20. Zimmer, P., Baumann, F. T., Oberste, M., Wright, P., Garthe, A., Schenk, A., . . . Wolf, F. (2016). Effects of Exercise Interventions and Physical Activity Behavior on Cancer Related Cognitive Impairments: A Systematic Review. *BioMed Research International, 2016*, 1820954. doi:https://dx.doi.org/10.1155/2016/1820954

Full Search Strategies

Ovid MEDLINE(R) ALL 1946 to March 12, 2024

1. exp Neoplasms/ or Cancer Survivors/ or Cancer Care Facilities/ or Oncology Service, Hospital/ or exp Medical Oncology/ or Oncology Nursing/ or Cancer Pain/
2. (cancer* or carcinoma* or glioblastoma* or leuk?emia* or lymphoma* or malignan* or neoplasm* or tumo?r or tumo?rs or oncolog*).ti,ab,kf.
3. 1 or 2
4. cognition/ or awareness/ or cognitive reserve/ or comprehension/ or metacognition/ or processing speed/
5. cognition disorders/ or cognitive dysfunction/ or chemotherapy-related cognitive impairment/ or postoperative cognitive complications/
6. attention/ or executive function/
7. memory/ or memory, episodic/ or memory, long-term/ or memory, short-term/ or mental recall/
8. Mental Processes/ or Neurobehavioral Manifestations/ or exp Neuropsychological Tests/ or Problem Solving/
9. thinking/ or concept formation/ or judgment/
10. (chemobrain or chemo-brain or chemofog or chemo-fog or cognit* or concept formation or executive function* or metacognit* or memory or mental process*).ti,ab,kf.
11. (neurobehavioral manifest* or neurocognit* or neuropsychological test* or processing speed).ti,ab,kf.
12. 4 or 5 or 6 or 7 or 8 or 9 or 10 or 11
13. meta-analysis/ or "systematic review"/ or Systematic Reviews as Topic/
14. ((system* or literature) adj2 (review* or search)).ti,ab,kf.

15. (cochrane review or meta-analysis or meta-synthesis or narrative review* or quantitative review* or systematic overview* or prisma or medline or pubmed or embase).ti,ab,kf.
16. (meta analysis or "systematic review").pt.
17. 13 or 14 or 15 or 16
18. 3 and 12 and 17
19. (case reports or comment or editorial or letter or news).pt.
20. 18 not 19

Embase 1974 to 2024 March 12 (Ovid)

1. exp *neoplasm/ or *cancer survivor/ or *cancer patient/ or exp *cancer survival/ or exp *cancer therapy/ or *cancer center/ or exp *oncology/ or *oncology ward/
2. (cancer* or carcinoma* or glioblastoma* or leuk?emia* or lymphoma* or malignan* or neoplasm* or tumo?r or tumo?rs or oncolog*).ti,ab,kf.
3. 3., 1 or 2
4. *chemotherapy-related cognitive impairment/ or *cognitive defect/ or *mild cognitive impairment/ or *postoperative cognitive dysfunction/
5. *cognition/ or *attention/ or *cognitive reserve/ or *executive function/ or *memory/ or *metacognition/ or *processing speed/ or *thinking/ or exp *neuropsychological assessment/
6. (chemobrain or chemo-brain or chemofog or chemo-fog or cognit* or concept formation or executive function* or metacognit* or memory or mental process*).ti,ab,kf.
7. (neurobehavioral manifest* or neurocognit* or neuropsychological test* or processing speed).ti,ab,kf.
8. 4 or 5 or 6 or 7
9. "systematic review"/ or "systematic review (topic)"/ or meta analysis/ or "meta analysis (topic)"/
10. ((system* or literature) adj2 (review* or search)).ti,ab,kf.
11. (cochrane review or meta-analysis or meta-synthesis or narrative review* or quantitative review* or systematic overview* or prisma or medline or pubmed or embase).ti,ab,kf.
12. 9 or 10 or 11
13. 3 and 8 and 12
14. (book or chapter or conference or conference abstract or conference paper or "conference review" or editorial or letter or note).pt.
15. case report.ti.
16. 14 or 15
17. 13 not 16

Ovid Emcare 1995 to 2024 Week 10

1. exp *neoplasm/ or *cancer survivor/ or *cancer patient/ or exp *cancer survival/ or exp *cancer therapy/ or *cancer center/ or exp *oncology/ or *oncology ward/
2. (cancer* or carcinoma* or glioblastoma* or leuk?emia* or lymphoma* or malignan* or neoplasm* or tumo?r or tumo?rs or oncolog*).ti,ab,kf.
3. 1 or 2
4. *chemotherapy-related cognitive impairment/ or *cognitive defect/ or *mild cognitive impairment/ or *postoperative cognitive dysfunction/
5. *cognition/ or *attention/ or *cognitive reserve/ or *executive function/ or *memory/ or *metacognition/ or *processing speed/ or *thinking/ or exp neuropsychological test/
6. (chemobrain or chemo-brain or chemofog or chemo-fog or cognit* or concept formation or executive function* or metacognit* or memory or mental process*).ti,ab,kf.

7. (neurobehavioral manifest* or neurocognit* or neuropsychological test* or processing speed).ti,ab,kf.
8. 4 or 5 or 6 or 7
9. "systematic review"/ or "systematic review (topic)"/ or meta analysis/ or "meta analysis (topic)"/
10. ((system* or literature) adj2 (review* or search)).ti,ab,kf.
11. (cochrane review or meta-analysis or meta-synthesis or narrative review* or quantitative review* or systematic overview* or prisma or medline or pubmed or embase).ti,ab,kf.
12. 9 or 10 or 11
13. 3 and 8 and 12
14. (book or chapter or conference or conference abstract or conference paper or "conference review" or editorial or letter or note).pt.
15. case report.ti.
16. 14 or 15
17. 13 not 16

APA PsycInfo 1806 to March Week 2 2024 (Ovid)

1. exp neoplasms/ or oncology/
2. (cancer* or carcinoma* or glioblastoma* or leuk?emia* or lymphoma* or malignan* or neoplasm* or tumo?r or tumo?rs or oncolog*).ti,ab.
3. 1 or 2
4. cognitive processes/ or exp awareness/ or exp cognition/ or exp cognitive ability/ or cognitive processing speed/ or cognitive reserve/ or exp cognitive strategies/ or concentration/ or exp concept formation/ or exp executive function/ or exp judgment/ or metacognition/ or exp problem solving/ or exp thinking/ or exp cognitive assessment/
5. cognitive impairment/ or mild cognitive impairment/ or neurocognitive disorders/ or memory/
6. (chemobrain or chemo-brain or chemofog or chemo-fog or cognit* or concept formation or executive function* or metacognit* or memory or mental process*).ti,ab.
7. (neurobehavioral manifest* or neurocognit* or neuropsychological test* or processing speed).ti,ab.
8. 4 or 5 or 6 or 7
9. "systematic review"/ or "literature review"/ or meta analysis/
10. ((system* or literature) adj2 (review* or search)).ti,ab.
11. (cochrane review or meta-analysis or meta-synthesis or narrative review* or quantitative review* or systematic overview* or prisma or medline or pubmed or embase).ti,ab.
12. 9 or 10 or 11
13. 3 and 8 and 12

CINAHL (EBSCOhost)

S1. (MH "Cancer Survivors") OR (MH "Cancer Patients") OR (MH "Cancer Fatigue") OR (MH "Cancer Pain") OR (MH "Chemotherapy, Cancer+") OR (MH "Rehabilitation, Cancer")
S2. (MH "Neoplasms+") OR (MH "Oncology+") OR (MH "Oncology Nursing+")
S3. cancer* OR carcinoma* OR glioblastoma* OR leuk#emia* OR lymphoma* OR malignan* OR neoplasm* OR tumo#r OR tumo#rs OR oncolog*
S4. S1 OR S2 OR S3
S5. (MH "Cognition Disorders+") OR (MH "Chemotherapy-Related Cognitive Impairment") OR (MH "Mild Cognitive Impairment")
S6. (MH "Cognition") OR (MH "Processing Speed") OR (MH "Executive Function") OR (MH "Mental Processes") OR (MH "Thinking")

S7. (MH "Problem Solving") OR (MH "Memory") OR (MH "Neuropsychological Tests+")
S8. chemobrain OR chemo-brain OR chemofog OR chemo-fog OR cognit* OR "concept formation" OR "executive function*" OR metacognit* OR memory OR "mental process*"
S9. "neurobehavioral manifest*" OR neurocognit* OR "neuropsychological test*" OR "processing speed"
S10. S5 OR S6 OR S7 OR S8 OR S9
S11. (MH "Literature Review+") OR (MH "Systematic Review") OR (MH "Scoping Review") OR (MH "Meta Analysis")
S12. (system* OR literature) N2 (review* OR search)
S13. "cochrane review" OR meta-analysis OR meta-synthesis OR "narrative review*" OR "quantitative review*" OR "systematic overview*" OR prisma OR medline OR pubmed OR embase
S14. S11 OR S12 OR S13
S15. S4 AND S10 AND S14

Cochrane Library (Wiley)

#1. [mh Neoplasms] OR [mh ^"Cancer Survivors"] OR [mh ^"Cancer Care Facilities"] OR [mh ^"Oncology Service, Hospital"] OR [mh "Medical Oncology"] OR [mh ^"Oncology Nursing"] OR [mh ^"Cancer Pain"]
#2. cancer*:ti,ab OR carcinoma*:ti,ab OR glioblastoma*:ti,ab OR leuk?emia*:ti,ab OR lymphoma*:ti,ab OR malignan*:ti,ab OR neoplasm*:ti,ab OR tumo?r:ti,ab OR tumo?rs:ti,ab OR oncolog*:ti,ab
#3. #1 OR #2
#4. [mh ^cognition] OR [mh ^awareness] OR [mh ^"cognitive reserve"] OR [mh ^comprehension] OR [mh ^metacognition] OR [mh ^"processing speed"]
#5. [mh ^"cognition disorders"] OR [mh ^"cognitive dysfunction"] OR [mh ^"chemotherapy-related cognitive impairment"] OR [mh ^"postoperative cognitive complications"]
#6. [mh ^attention] OR [mh ^"executive function"]
#7. [mh ^memory] OR [mh ^"memory, episodic"] OR [mh ^"memory, long-term"] OR [mh ^"memory, short-term"] OR [mh ^"mental recall"]
#8. [mh ^"Mental Processes"] OR [mh ^"Neurobehavioral Manifestations"] OR [mh "Neuropsychological Tests"] OR [mh ^"Problem Solving"]
#9. [mh ^thinking] OR [mh ^"concept formation"] OR [mh ^judgment]
#10. chemobrain:ti,ab OR chemo-brain:ti,ab OR chemofog:ti,ab OR chemo-fog:ti,ab OR cognit*:ti,ab OR "concept formation":ti,ab OR ("executive" NEXT function*):ti,ab OR metacognit*:ti,ab OR memory:ti,ab OR ("mental" NEXT process*):ti,ab
#11. ("neurobehavioral" NEXT manifest*):ti,ab OR neurocognit*:ti,ab OR ("neuropsychological" NEXT test*):ti,ab OR "processing speed":ti,ab
#12. #4 OR #5 OR #6 OR #7 OR #8 OR #9 OR #10 OR #11
#13. (system*:ti,ab OR literature:ti,ab) NEAR/2 (review*:ti,ab OR search:ti,ab)
#14. "cochrane review":ti,ab OR meta-analysis:ti,ab OR meta-synthesis:ti,ab OR ("narrative" NEXT review*):ti,ab OR ("quantitative" NEXT review*):ti,ab OR ("systematic" NEXT overview*):ti,ab OR prisma:ti,ab OR medline:ti,ab OR pubmed:ti,ab OR embase:ti,ab
#15. MeSH descriptor: [Systematic Review] explode all trees
#16. #13 OR #14 OR #15
#17. #3 AND #12 AND #16

PART 2

Supplementary Table 1. ***Relevant Primary Studies from the SRs***

Primary Study	Number of Occurrences in SRs
Barakat LP, Hetzke JD, Foley B, Carey ME, Gyato K, Phillips PC. Evaluation of a social skills training group intervention with children treated for brain tumors: a pilot study. J Pediatr Psychol 2003;28:299- 307.	2
C. Zucchella, A. Capone, V. Codella et al., “Cognitive rehabilitation for early post-surgery inpatients affected by primary brain tumor: a randomized, controlled trial,” Journal of Neuro- Oncology, vol. 114, no. 1, pp. 93–100, 2013.	2
Cešeiko R, Eglītis J, Srebnijs A, et al. The impact of maximal strength training on quality of life among women with breast cancer undergoing treatment. Exp Oncol. 2019;41:166–72.	2
Cox E, Bells S, Timmons BW, Laughlin S, Bouffet E, de Medeiros C, Beera K, Harasym D, Mabbott DJ. A controlled clinical crossover trial of exercise training to improve cognition and neural communication in pediatric brain tumor survivors. Clin Neurophysiol. 2020;131:1533–47. https://doi.org/10.1016/j. clinph.2020.03.027	2
Dolbeault S, Cayrou S, BrE` dart A, et al. The effectiveness of a psycho-educational group after early-stage breast cancer treat- ment: results of a randomized French study. Psychooncology 2009;18:647–56.	2
Ehlers DK, Rogers LQ, Courneya KS, Robbs RS, McAuley E. Effects of BEAT Cancer randomized physical activity trial on subjective memory impairments in breast cancer survivors. Psycho Oncol. 2018;27:687–690. https://doi.org/10.1002/ pon.4438.	2
Fitzpatrick TR, Edgar L, Holcroft C. Assessing the relationship between physical fitness activities, cognitive health, and qual- ity of life among older cancer survivors. J Psychosoc Oncol. 2012;30:556–72. https://doi.org/10.1080/07347332.2012.703768	2
Freeman LW, White R, Ratcliff CG, et al. A randomized trial comparing live and telemedicine deliveries of an imagery-based behavioral intervention for breast cancer survivors: reducing symptoms and barriers to care. Psycho Oncol. 2015;24: 910–918. https://doi.org/10.1002/pon.3656.	2
Goedendorp MM, Peters MEWJ, Gielissen MFM, Witjes JA, Leer JW, Verhagen CAHHVM, et al. Is increasing physical activity necessary to diminish fatigue during cancer treatment? comparing cognitive behavior therapy and a brief nursing intervention with usual care in a multicenter randomized controlled trial. Oncolo- gist. 2010;15:1122–32.	2
Gokal K, Wallis D, Ahmed S, Boiangiu I, Kancherla K, Munir F. Effects of a self-managed home-based walking interven- tion on psychosocial health outcomes for breast cancer patients receiving chemotherapy: a randomised controlled trial. Sup- port Care Cancer. 2016;24:1139–66. https://doi.org/10.1007/ s00520-015-2884-5	2
Han EY, Chun MH, Kim BR, Kim HJ. Functional improvement after 4-week rehabilitation therapy and effects of attention defi- cit in brain tumor patients: comparison with subacute stroke patients. Ann Rehabil Med. 2015;39:560-569. doi:10.5535/ arm.2015.39.4.560	2
Henke CC, Cabri J, Fricke L, et al. Strength and endurance training in the treatment of lung cancer patients in stages IIIA/IIIB/IV. Support Care Cancer. 2014;22(1):95–101. https://doi.org/10.1007/s00520-013- 1925-1. Medline:23995813	2
Hocking, M.C.; Paltin, I.; Quast, L.F.; Barakat, L.P. Acceptability and Feasibility in a Pilot Randomized Clinical Trial of Computerized Working Memory Training and Parental Problem-Solving Training with Pediatric Brain Tumor Survivors. J. Pediatr. Psychol. 2019, 44, 669–678.	2
Jacobs W, Schagen SB, Thijssen M, Das E. Preventing adverse information effects on health outcomes: a self-affirmation intervention reduced information-induced cognitive decline in gastrointestinal cancer patients. Soc Sci Med. 2019;226:47-55. 10.1016/j.socscimed.2019.02.013	2
Jong MC, Boers I, Schouten van der Velden AP, Meij SV, Goker E, Timmer-Bonte A, van Wietmarschen HA (2018) A randomized study of yoga for fatigue and quality of life in women with breast cancer undergoing (neo) adjuvant chemotherapy. J Altern Complement Med 24(9–10):942–953	2
Kerns KA, Thomson J. Implementation of a compensatory memory sys- tem in a school age child with severe memory impairment. Pediatr Rehabil. 1998;2(2):77–87.	2
Kesler, S. R., Lacayo, N. J., & Jo, B. (2011). A pilot study of an online cognitive rehabilitation program for executive function skills in children with cancer-related brain injury. Brain Injury, 25(1), 101–112. doi:10.3109/02699052.2010.536194	2
Knobf MT, Thompson A, Fennie K, Erdos D. The effect of a community-based exercise intervention on symtoms and quality of life. Cancer Nurs. 2013;37:E43–E50.	2
Leach HJ, Danyluk JM, Nishimura KC, Culos-Reed N. Benefits of 24 versus 12 weeks of exercise and wellness programming for women undergoing treatment for breast cancer. Support Care Cancer. 2016;24(11):4597-4606. 10.1007/s00520-016-3302-3	2
Leach HJ, Danyluk JM, Nishimura KC, Culos-Reed SN. Evalu- ation of a community-based exercise program for breast cancer patients undergoing treatment. Cancer Nurs. 2015;38:417–25. https://doi.org/10.1097/NCC.0000000000000217	2
Matthews EE, et al. Cognitive behavioral therapy for insomnia outcomes in women after primary breast cancer treatment: a raddomized, controlled trial. Oncol Nurs Forum. 2014;41(3):241–53.	2
May AM, Korstjens I, van Weert E, van den Borne B, Hoekstra-Weebers JE, van der Schans CP, et al. Long-term effects on cancer survivors’ quality of life of physical training versus physical training combined with cognitive-behavioral therapy: results from a randomized trial. Support Care Cancer 2009;17:653–63	2
Mendoza, L.K.; Ashford, J.M.; Willard, V.W.; Clark, K.N.; Martin-Elbahesh, K.; Hardy, K.K.; Merchant, T.E.; Jeha, S.; Wang, F.; Zhang, H.; et al. Social Functioning of Childhood Cancer Survivors after Computerized Cognitive Training: A Randomized Controlled Trial. Children 2019, 6, E105	2
Meneses K, Benz R, Bail JR, et al. Speed of processing training in middle-aged and older breast cancer survivors (SOAR): results of a randomized controlled pilot. Breast Cancer Res Treat 2018;168:259–67.	2
Miotto EC, Balardin JB, Vieira G, et al. Right inferior frontal gyrus activation is associated with memory improvement in pa- tients with left frontal low-grade glioma resection. PLoS One. 2014;9:e105987. doi:10.1371/journal.pone.0105987	2
Moore IM, Hockenberry MJ, Anhalt C, McCarthy K, Krull KR. Mathematics intervention for prevention of neurocognitive deficits in childhood leukemia. Pediatr Blood Cancer 2012;59:278-84.	2
Myers JS, Cook-Wiens G, Baynes R, et al. Emerging from the haze: a multicenter, controlled pilot study of a multidimensional, psychoe- ducation-based cognitive rehabilitation intervention for breast can- cer survivors delivered with telehealth conferencing. Arch Phys Med Rehabil. 2020;101(6):948–959.	2

Palmer, S. L., Leigh, L., Ellison, S. C., Onar-Thomas, A., Wu, S., Qaddoumi, I., … Gajjar, A. (2014). Feasibility and efficacy of a computer-based intervention aimed at preventing reading decoding deficits among children undergoing active treatment for medulloblastoma: Results of a randomized trial. Journal of Pediatric Psychology, 39(4), 450–458. doi:10.1093/jpepsy/jst095	2
Peterson RK, Longo C, Cunningham T, Janzen L, Guger S, Monteiro L, et al. Impact of home-based cognitive or academic intervention on working memory and mathematics outcomes in pediatric brain tumor survivors: the keys to succeed pilot randomized controlled clinical trial. Child Neuropsychol (2022) 28(8):1116–40. doi: 10.1080/09297049.2022.2061933	2
Rahmani S, Talepasand S, Ghanbary-Motlagh A. Comparison of effectiveness of the metacognition treatment and the mindfulness-based stress reduction treatment on global and specific life quality of women with breast cancer. Iran J Cancer Prev. 2014;7(4):184–96.	2
Rahmani S, Talepasand S. The effect of group mindfulness - based stress reduction program and conscious yoga on the fatigue severity and global and specific life quality in women with breast cancer. Med J Islam Repub Iran. 2015;29:175.	2
Rottmann N, Dalton SO, Bidstrup PE, Würtzen H, Høybye MT, Ross L, et al. No improvement in distress and quality of life following psychosocial cancer rehabilitation. A randomised trial. Psychooncology 2012;21:505–14.	2
Sabel M, Sjölund A, Broeren J, et al. Active video gaming improves body coordination in survivors of childhood brain tumours. Disabil Rehabil 2016; 38: 2073–84.	2
Salerno EA, Culakova E, Kleckner AS, et al. Physical activity patterns and relationships with cognitive function in patients with breast cancer before, during, and after chemotherapy in a prospective, nationwide study. J clin oncol. 2021;39(29):3283–3292.	2
Schneider CM, Hsieh CC, Sprod L, Carter SD, Hayward R. Effects of supervised exercise training on cardiopulmonary function and fatigue in breast cancer survivors during and after treatment. Cancer. 2007;110:918–825.	2
Siciliano RE, Thigpen JC, Desjardins L, Cook JL, Steele EH, Gruhn MA, et al. Working memory training in pediatric brain tumor survivors after recent diagnosis: challenges and initial effects. Appl Neuropsychol Child (2022) 11(3):412–21. doi: 10.1080/21622965.2021.1875226	2
Szulc-Lerch KU, Timmons BW, Bouffet E, et al. Repairing the brain with physical exercise: cortical thickness and brain volume increases in long-term pediatric brain tumor survivors in response to a structured exercise intervention. Neuroimage Clin. 2018;18:972–985.	2
Van Der Linden SD, Sitskoorn MM, Rutten GJM, Gehring K. Feasibility of the evidence-based cognitive telerehabilitation program Remind for patients with primary brain tumors. J Neurooncol. 2018;137(3):523-532. doi:10.1007/s11060-017-2738-8	2
Van Vulpen JK, Velthuis MJ, Steins Bisschop CN, et al. Effects of an exercise program in colon cancer patients undergoing chemotherapy. Med Sci Sports Exerc 2015;48:767e775.	2
van Waart H, Stuiver MM, van Harten WH, et al. Effect of low-inten- sity physical activity and moderate-to high-intensity physical exercise during adjuvant chemotherapy on physical fitness, fatigue, and che- motherapy completion rates: results of the PACES randomized clinical trial. J Clin Oncol. 2015;33:1918–27.	2
van Weert E, May AM, Korstjens I, et al. Cancer-related fatigue and rehabilitation: a randomized controlled multicenter trial comparing physical training combined with cognitive-behavioral therapy with physical training only and with no intervention. Physical Therapy. 2010;90:1413–1425.	2
van't Hooft I, Norberg AL. SMART cognitive training combined with a parental coaching programme for three children treated for medulloblas- toma. NeuroRehabilitation. 2010;26(2):105–113.	2
Voss M, Wenger KJ, Mettenheim von N, et al. Short-term fast- ing in glioma patients: analysis of diet diaries and metabolic parameters of the ERGO2 trial. Eur J Nutr. 2022;61:477-487. doi:10.1007/s00394-021-02666-1	2
Wade SL, Narad ME, Moscato EL, LeBlond EI, King JA, Raj SP, et al. A survivor's journey: preliminary efficacy of an online problem-solving therapy for survivors of pediatric brain tumor. Pediatr Blood Cancer (2020) 67(2):e28043. doi: 10.1002/pbc.28043	2
Wei X, Yuan R, Yang J, et al. Effects of Baduanjin exercise on cognitive function and cancer-related symptoms in women with breast cancer receiving chemotherapy: a randomised controlled trial. Support Care Cancer. 2022;30(7):6079-6091. 10.21203/rs.3.rs-1044265/v1	2
Wyant S. Feasibility and acceptability of a computerized working memory training in breast Cancer survivors (doctoral dissertation); 2017.	2
Adamsen L, Quist M, Andersen C, et al. Effect of a multimodal high intensity exercise intervention in cancer patients undergoing chemotherapy: Randomised controlled trial. BMJ. 2009;339:b3410. https://doi.org/10.1136/bmj.b3410. Medline:19826172	3
Bade BC, Gan G, Li F, et al. Randomized trial of physical activity on quality of life and lung cancer biomarkers in patients with advanced stage lung cancer: a pilot study. BMC Cancer. 2021;21(1):352.	3
Bryant AL, Deal AM, Battaglini CL, et al. The effects of exercise on patient-reported outcomes and performance-based physical function in adults with acute leukemia undergoing induction therapy: Exercise and Quality of Life in Acute Leukemia (EQUAL). Integr Cancer Ther. 2018;17(2):263–70. https://doi. org/10.1177/1534735417699881. Medline:28627275	3
Buffart LM, Newton RU, Chinapaw MJ, et al. The effect, moderators, and mediators of resistance and aerobic exercise on health-related quality of life in older long-term survivors of prostate cancer. Cancer. 2015;121(16):2821–2830.	3
Carayol M, Ninot G, Senesse P, Bleuse JP, Gourgou S, Sancho-Garnier H, et al. Short- and long-term impact of adapted physical activity and diet counseling during adjuvant breast cancer therapy: the "APAD1" randomized controlled trial. BMC Cancer 2019;19:737.	3
Carlson-Green B, Puig J, Bendel A. Feasibility and efficacy of an extended trial of home-based working memory training for pediatric brain tumor survivors: a pilot study. Neurooncol Pract (2017) 4(2):111–20. doi: 10.1093/nop/npw015	3
Cimprich B, Ronis DL. An environmental intervention to restore attention in women with newly diagnosed breast cancer. Cancer Nurs 284–92.	3
Cimprich B. Development of an intervention to restore attention in cancer patients. Cancer Nurs 1993;16:83-92.	3
Dimeo FC, Thomas F, Raabe-Menssen C, Propper F, Mathias M. Effect of aerobic exercise and relaxation training on fatigue and physical performance of cancer patients after surgery. A ran- domised controlled trial. Support Care Cancer. 2004;12:774–9. https://doi.org/10.1007/s00520-004-0676-4	3
Gehring K, Stuiver MM, Visser E, et al. A pilot randomized con- trolled trial of exercise to improve cognitive performance in pa- tients with stable glioma: a proof of concept. Neuro-Oncology. 2020;22:103-115. doi:10.1093/neuonc/noz178	3
Henneghan AM, Becker H, Harrison ML, et al. A randomized control trial of meditation compared to music listening to improve cognitive function for breast cancer survivors: feasibility and acceptability. Complement Ther Clin Pract. 2020;41.	3
Kasatkin V, Deviaterikova A, Shurupova M, Karelin A. The feasibility and efficacy of short-term visual-motor training in pediatric posterior fossa tumor survivors. Eur J Phys Rehabil Med (2022) 58(1):51–9. doi: 10.23736/S1973-9087.21.06854-4	3
Komatsu H, Yagasaki K, Yamauchi H, et al. A self-directed home yoga programme for women with breast cancer during chemotherapy: A feasibility study: yoga for patients undergoing chemotherapy. Int J Nurs Pract. 2016;22(3):258–66. https://doi.org/10.1111/ijn.12419. Medline:26643264	3

Korstjens I, May AM, van Weert E, Mesters I, Tan F, Ros WJG, et al. Quality of life after self-management cancer rehabilitation: a randomized controlled trial comparing physical and cognitive- behavioral training versus physical training. Psychosom Med. 2008;70:422–9.	3
McDougall Jr. GJ. Memory improvement program for elderly cancer survivors. Ger- iatr Nurs. 2001;22:185–190.	3
Pasyar N, Barshan Tashnizi N, Mansouri P, Tahmasebi S. Effect of yoga exercise on the quality of life and upper extremity vol- ume among women with breast cancer related lymphedema: a pilot study. Eur J Oncol Nurs. 2019;42:103–9. https://doi. org/10.1016/j.ejon.2019.08.008	3
Poppelreuter M, Weis J, Mumm A, Orth HB, Bartsch HH. Rehabilitation of therapy-related cognitive deficits in patients after hemato- poietic stem cell transplantation. Bone Marrow Transplant 2008;41: 79-90.	3
Rogers LQ, Hopkins-Price P, Vicari S, et al. A randomized trial to increase physical activity in breast cancer survivors. Med Sci Sports Exerc. 2009;41:935–946.	3
Shari NI, Zainal NZ, Ng CG. Effects of brief acceptance and commitment therapy (ACT) on subjective cognitive impairment in breast cancer patients undergoing chemotherapy. J Psychosoc Oncol. 2020;39(6):1-20. 10.1080/07347332.2020.1856283	3
Van der Gucht K, Ahmadoun S, Melis M, et al. Effects of a mind- fulness-based intervention on cancer-related cognitive impairment: results of a randomized controlled functional magnetic resonance imaging pilot study. Cancer. 2020;126(18):4246–4255.	3
van der Linden SD, Rutten GJM, Dirven L, et al. eHealth cognitive rehabilitation for brain tumor patients: results of a randomized controlled trial. J Neurooncol. 2021;154(3):315-326. doi:10.1007/s1106 0-021-03828-1	3
Alvarez J, Meyer F, Granoff DL, Lundy A. The effect of EEG biofeedback on reducing postcancer cognitive impairment. Integr Cancer Ther. 2013; 12(6):475Y487	4
Backman M, Wengström Y, Johansson B, et al. A randomized pilot study with daily walking during adjuvant chemotherapy for patients with breast and colorectal cancer. Acta Oncol. 2014;53(4):510–520.	4
Benzing V, Spitzhüttl J, Siegwart V, et al. Effects of cognitive training and exergaming in pediatric cancer survivors—a randomized clinical trial. Med Sci Sports Exerc 2020; 52: 2293–302.	4
Cantarero-Villanueva I, Fernandez-Lao C, Cuesta-Vargas AI, Del Moral-Avila R, Fernandez-de-Las-Penas C, Arroyo-Morales M (2013) The effectiveness of a deep water aquatic exercise program in cancer-related fatigue in breast cancer survivors: a randomized controlled trial. Arch Phys Med Rehabil 94(2):221–230	4
Conklin HM, Ogg RJ, Ashford JM, Scoggins MA, Zou P, Clark KN, et al. Computerized cognitive training for amelioration of cognitive late effects among childhood cancer survivors: A randomized controlled trial. J Clin Oncol 2015;33:3894-902.	4
de Ruiter MA, Oosterlaan J, Schouten-van Meeteren AY, Maurice-Stam H, van Vuurden DG, Gidding C, et al. Neurofeedback ineffective in paediatric brain tumour survivors: results of a double-blind randomised placebo-controlled trial. Eur J Cancer (2016) 64:62–73. doi: 10.1016/j.ejca.2016.04.020	4
Duval A, Davis CG, Khoo EL, et al. Mindfulness-based stress reduction and cognitive function among breast cancer survivors: a randomised control trial. Cancer. 2022;128(13):2520-2528. 10.1002/cncr.34209	4
Ferguson RJ, et al. A randomized trial of videoconference-delivered cognitive behavioral therapy for survivors of breast cancer with self-reported cognitive dysfunction. Cancer 2016;122(11):1782–91.	4
Galiano-Castillo N, Cantarero-Villanueva I, Fernandez-Lao C, Ariza-Garcia A, Diaz-Rodriguez L, Del-Moral-Avila R, Arroyo- Morales M. Telehealth system: a randomized controlled trial eval- uating the impact of an internet-based exercise intervention on quality of life, pain, muscle strength, and fatigue in breast cancer survivors. Cancer. 2016;122:3166–74. https://doi.org/10.1002/ cncr.30172	4
Hacker ED, Larson J, Kujath A, Peace D, Rondelli D, Gaston L. Strength training following hematopoietic stem cell transplan- tation. Cancer Nurs. 2011;34:238–49. https://doi.org/10.1097/ NCC.0b013e3181fb3686	4
Hardy KK, Willard VW, Bonner MJ. Computerized cognitive train- ing in survivors of childhood cancer: a pilot study. J Pediatr Oncol Nurs. 2011;28(1):27–33.	4
Howell CR, Krull KR, Partin RE, et al. Randomized web-based physical activity intervention in adolescent survivors of childhood cancer. Pediatr Blood Cancer 2018; 65: e27216.	4
Miotto EC, Savage CR, Evans JJ, et al. Semantic strategy train- ing increases memory performance and brain activity in pa- tients with prefrontal cortex lesions. Clin Neurol Neurosurg. 2013;115:309-316. doi:10.1016/j.clineuro.2012.05.024	4
Oechsle K , Aslan Z , Suesse Y, et al. Multimodal exercise training during myeloablative chemotherapy: A prospective randomized pilot trial. Support Care Cancer. 2014;22(1):63–9. https://doi.org/ 10.1007/s00520-013-1927-z. Medline:2398949	4
Patel SK, Katz ER, Richardson R, et al. Cognitive and problem solving training in children with cancer: a pilot project. J Pediatr Hematol Oncol. 2009;31(9):670–677.	4
Reich RR, Lengacher CA, Alinat CB, et al. Mindfulness-based stress reduction in post-treatment breast Cancer patients: immediate and sustained effects across multiple symptom clusters. J Pain Symptom Manag. 2017;53(1):85–95.	4
Richard NM, Bernstein LJ, Mason WP, et al. Cognitive rehabili- tation for executive dysfunction in brain tumor patients: a pilot randomized controlled trial. J Neuro-Oncol. 2019;142:565-575. doi:10.1007/s11060-019-03130-1	4
Saarto T, Penttinen HM, Sievänen H, et al. Effectiveness of a 12-month exercise program on physical performance and quality of life in breast cancer survivors Anticancer Research. 2012;32:3875–3884.	4
Salerno EA, Rowland K, Hillman CH, Trinh L, Kramer AF, McAuley E. Dose-response effects of acute aerobic exercise duration on cognitive function in patients with breast cancer: a randomized crossover trial. Front Psychol. 2020;11:1500.	4
Salerno EA, Rowland K, Kramer AF, McAuley E. Acute aerobic exercise effects on cognitive function in breast cancer survivors: a randomized crossover trial. BMC Cancer. 2019;19(1):371.	4
Sherer M, Meyers CA, Bergloff P. Efficacy of postacute brain injury rehabilitation for patients with primary malignant brain tumors. Cancer 1997;80:250-7.	4
Yang S, Chun MH, Son YR. Effect of Virtual Reality on Cognitive Dysfunction in Patients With Brain Tumor. Ann Rehabil Med. 2014;38(6):726. doi:1 0.5535/arm.2014.38.6.726	4
Baumann FT, Drosselmeyer N, Leskaroski A, et al. 12-week resistance training with breast cancer patients during chemotherapy: effects on cognitive abilities. Breast Care. 2011;6(2):142–3. https://doi. org/10.1159/000327505.	5
Becker H, Henneghan AM, Volker DL, et al. A pilot study of a cognitive-behavioral intervention for breast cancer survivors. Oncol Nurs Forum. 2017;44(2):255-264.	5
Ding K, Zhang X, Zhao J, Zuo H, Bi Z, Cheng H. Managing Cancer and Living Meaningfully (CALM) intervention on chemotherapy-related cognitive impairment in breast cancer survivors. Integr Cancer Ther. 2020; 19:1534735420938450.	5

Galiano-Castillo N, Arroyo-Morales M, Lozano-Lozano M, et al. Effect of an internet-based telehealth system on functional capa- city and cognition in breast cancer survivors: a secondary analysis of a randomized controlled trial. Support Care Cancer. 2017;25 (11):3551–3559.	5
Galvao DA, Taafe DR, Spry N, Joseph D, Newton RU. Combined resistance and aerobic exercise program reverses muscle loss in men undergoing androgen suppression therapy for prostate cancer without bone metastases: A randomized controlled trial. Journal of Clincal Oncology. 2010;28:340–347.	5
Hardy KK, Willard VW, Allen TM, Bonner MJ. Working memory training in survivors of pediatric cancer: A randomized pilot study. Psychooncology 2013;22:1856-65.	5
Hassler MR, Elandt K, Preusser M, et al. Neurocognitive train- ing in patients with high-grade glioma: a pilot study. J Neuro- Oncol. 2010;97:109-115. doi:10.1007/s11060-009-0006-2	5
Knols RH, de Bruin ED, Uebelhart D, Aufdemkampe G, Schanz U, StennerLiewen F, et al. Effects of an outpatient physical exercise program on hematopoietic stem-cell transplantation recipients: a randomized clinical trial. Bone Marrow Transplant 2011;46(9):1245–55.	5
Maschio, M., Dinapoli, L., Fabi, A., Giannarelli, D., & Cantelmi, T. (2015). Cognitive rehabilitation training in patients with brain tumor-related epilepsy and cognitive deficits: A pilot study. Journal of Neuro-Oncology, 125(2), 419–426. https://doi.org/10.1007/s11060-015-1933-8	5
Riggs L, Piscione J, Laughlin S, Cunningham T, Timmons BW, Courneya KS, Bartels U, Skocic J, de Medeiros C, Liu F, Persa- die N, Scheinemann K, Scantlebury N, Szulc KU, Bouffet E, Mabbott DJ. Exercise training for neural recovery in a restricted sample of pediatric brain tumor survivors: a controlled clinical trial with crossover of training versus no training. J Neurooncol. 2017;19:440–50. https://doi.org/10.1093/neuonc/now177	5
Schmidt T, Weisser B, Dürkop J, et al. Comparing endurance and resistance training with standard care during chemotherapy for patients with primary breast cancer. Anticancer Res. 2015;35(10):5623–9.	5
Schuurs A, Green HJ (2013) A feasibility study of group cognitive rehabilitation for cancer survivors: enhancing cognitive function and quality of life. Psycho-Oncology 22(5):1043–1049. https:// doi.org/10.1002/pon.3102	5
Wiskemann J, Dreger P, Schwerdtfeger R, Bondong A, Huber G, Kleindienst N, et al. Effects of a partly self-administered exercise program before, during, and after allogeneic stem cell transplanta- tion. Blood. 2011;117:2604–13.	5
Bellens A, Roelant E, Sabbe B, et al. A video-game based cognitive training for breast cancer survivors with cognitive impairment: a prospective randomized pilot trial. Breast 2020;53:23–32.	6
Butler RW, Copeland DR, Fairclough DL, et al. A multicenter, randomized clinical trial of a cognitive remediation program for childhood survivors of a pediatric malignancy. J Consult Clin Psychol 2008;76:367-78.	6
Butler RW, Copeland DR. Attentional processes and their remediation in children treated for cancer: a literature review and the development of a therapeutic approach. J Int Neuropsychol Soc 2002;8:115-24.	6
Culos-Reed SN, Carlson LE, Daroux LM, Hately-Aldous S. A pilot study of yoga for breast cancer survivors: physical and psy- chological benefits. Psycho-Oncol. 2006;15:891–7. https://doi. org/10.1002/pon.1021	6
Dos Santos M, Hardy-Léger I, Rigal O, et al. Cognitive rehabilitation program to improve cognition of cancer patients treated with chemotherapy: {A} 3-arm randomized trial. Cancer. 2020;126 (24):5328–5336.	6
Gehring, K., Sitskoorn, M.M., Gundy, C.M., Sikkes, S.A., Klein, M., Postma, T.J., . . . Aaronson, N.K. (2009). Cognitive rehabilitation in patients with gliomas: A randomized, controlled trial. Journal of Clinical Oncology, 27, 3712–3722. doi:10.1200/ jco.2008.20.5765	6
Larkey LK, Roe DJ, Smith L, et al. Exploratory outcome assessment of Qigong/Tai Chi Easy on breast cancer survivors. Complement Ther Med. 2016;29:196–203.	6
McDougall GJ, Becker H, Acee TW, Vaughan PW, Delville CL (2011) Symptom management of affective and cognitive disturbance with a group of cancer survivors. Arch Psychiatr Nurs 25(1):24–35. https://doi.org/10.1016/j.apnu.2010.05.004	6
Northey JM, Pumpa KL, Quinlan C, et al. Cognition in breast cancer survivors: a pilot study of interval and continuous exercise. J Sci Med Sport. 2019;22:580–585. https:// doi.org/10.1016/j.jsams.2018.11.026.	6
Oh B, Butow PN, Mullan BA, et al. Effect of medical Qigong on cognitive function, quality of life, and a biomarker of inflammation in cancer patients: a randomized controlled trial. Support Care Cancer. 2012;20:1235–1242.	6
Peterson BM, Johnson C, Case KR, et al. Feasibility of a combined aerobic and cognitive training intervention on cognitive function in cancer survivors: a pilot investigation. Pilot Feasibility Stud. 2018;4:50.	6
Reid-Arndt AA, Matsuda S, Cox CR. Tai chi effects on neuropsychological, emotional, and physical functioning following cancer treatment: A pilot study. Complementary Therapies in Clinical Practice. 2012;18:26–30	6
Sabel M, Sjolund A, Broeren J, Arvidsson D, Saury JM, Gil- lenstrand J, Emanuelson I, Blomgren K, Lannering B. Effects of physically active video gaming on cognition and activities of daily living in childhood brain tumor survivors: a random- ized pilot study. Neuro-Oncol Pract. 2017;4:98–110. https://doi. org/10.1093/nop/npw020	6
Steindorf K, Schmidt ME, Klassen O, et al. Randomized, controlled trial of resistance training in breast cancer patients receiving adjuvant radiotherapy: Results on cancer-related fatigue and quality of life. Ann Oncol. 2014;25:2237–2243	6
Tong T, et al. Efficacy of acupuncture therapy for chemotherapy-related cognitive impairment in breast cancer patients. Med Sci Monit 2018;24:2919–27.	6
Galantino ML, Greene L, Daniels L, Dooley B, Muscatello L, O'Donnell L. Longitudinal impact of yoga on chemotherapy- related cognitive impairment and quality of life in women with early stage breast cancer: a case series. Explore. 2012;8:127–35. https://doi.org/10.1016/j.explore.2011.12.001	7
Janelsins MC, Peppone LJ, Heckler CE, et al. YOCAS(c)(R) Yoga Reduces Self-reported Memory Difficulty in Cancer Survivors in a Nationwide Randomized Clinical Trial: Investigating Relationships Between Memory and Sleep. Integr Cancer Ther. 2016;15:263–271.	7
Johns SA, Von Ah D, Brown LF, Beck-Coon K, Talib TL, Alyea JM, et al. Randomized controlled pilot trial of mindfulness-based stress reduction for breast and colorectal cancer survivors: effects on cancer-related cognitive impairment. J Cancer Surviv 2016;10(3):437e48.	7
King S, Green HJ (2015) Psychological intervention for improving cognitive function in cancer survivors: a literature review and ran- domized controlled trial. Front Oncol 5:72. https://doi.org/10.3389/ fonc.2015.00072	7
Mijwel S, Backman M, Bolam KA, et al. Adding high-intensity inter- val training to conventional training modalities: optimizing health- related outcomes during chemotherapy for breast cancer: the OptiTrain randomized controlled trial. Breast Cancer Res Treat. 2018;168(1):79–93.	7
Oh B, Butow P, Mullan B, Clarke S, Beale P, Pavlakis N, Kothe E, Lam L, Rosenthal D. Impact of medical qigong on quality of life, fatigue, mood and inflammation in cancer patients: a randomized controlled trial. Ann Oncol. 2010;21:608–14. https:// doi.org/10.1093/annonc/mdp479	7
Park JH, et al. Effects of compensatory cognitive training intervention for breast can- cer patients undergoing chemotherapy: a pilot study. Support Care Cancer 2017;25 (6):1887–96.	7
Bray VJ, Dhillon HM, Bell ML, et al. Evaluation of a web-based cognitive rehabilita- tion program in cancer survivors reporting cognitive symptoms after chemother- apy. J Clin Oncol. 2017;35:217–225.	8
Cherrier MM, Anderson K, David D, et al. A randomized trial of cognitive rehabilita- tion in cancer survivors. Life Sci. 2013;93:617–622.	8

Hartman SJ, Nelson SH, Myers E, et al. Randomized controlled trial of increasing physical activity on objectively measured and self- reported cognitive functioning among breast cancer survivors: the memory & motion study. Cancer. 2018;124(1):192–202.	8
Locke DE, Cerhan JH, Wu W, et al. Cognitive rehabilitation and problem-solving to improve quality of life of patients with primary brain tumors: a pilot study. J Sup- port Oncol. 2008;6:383–391.	8
Myers JS, Mitchell M, Krigel S, et al. Qigong intervention for breast cancer survivors with complaints of decreased cognitive function. Support Care Cancer. 2018;27:1–9.	8
Poppelreuter M, Weis J, Bartsch HH (2009) Effects of specific neuropsychological training programs for breast cancer patients after adjuvant chemotherapy. J Psychosoc Oncol 27(2):274–296. https://doi.org/10.1080/07347330902776044	8
Vadiraja HS, Rao MR, Nagarathna R, et al. Effects of yoga program on quality of life and affect in early breast cancer patients undergoing adjuvant radiotherapy: a randomized controlled trial. Complement Ther Med. 2009;17: 274–280.	8
Wu LM, Amidi A, Tanenbaum ML, et al. Computerized cognitive training in prostate cancer patients on androgen deprivation ther- apy: a pilot study. Support Care Cancer. 2018;26(6):1917–1926.	8
Campbell KL, et al. Effect of aerobic exercise on cancer-associated cognitive impairment: a proof-of-concept RCT. Psychooncology 2018;27(1):53–60.	9
Goedendorp MM, Knoop H, Gielissen MF, Verhagen CA, Bleijenberg G. The effects of cognitive behavioral therapy for postcancer fatigue on perceived cognitive dis- abilities and neuropsychological test performance. J Pain Symptom Manage. 2014;47:35–44.	9
Damholdt MF, Mehlsen M, O'Toole MS, Andreasen RK, Pedersen AD, Zachariae R (2016) Web-based cognitive training for breast cancer survivors with cognitive complaints—a randomized con- trolled trial. Psycho-Oncology 25(11):1293–1300. https://doi.org/ 10.1002/pon.4058	10
Miki E, Kataoka T, Okamura H. Feasibility and efficacy of speed- feedback therapy with a bicycle ergometer on cognitive function in elderly cancer patients in japan. Psycho-Oncol. 2014;23:906– 13. https://doi.org/10.1002/pon.3501	10
Schmidt ME, Wiskemann J, Armbrust P, et al. Effects of resistance exercise on fatigue and quality of life in breast cancer patients undergoing adjuvant chemotherapy: A randomized controlled trial: Effects of resistance exercise on fatigue. Int J Cancer. 2015;137(2):471–80. https://doi.org/10.1002/ijc.29383. Medline:25484317	10
Gokal K, et al. Does walking protect against decline in cognitive functioning among breast cancer patients undergoing chemotherapy? Results from a small randomised controlled trial. PLoS One 2018;13(11):e0206874.	12
Derry HM, Jaremka LM, Bennett JM, et al. Yoga and self-reported cognitive problems in breast cancer survivors: a randomized con- trolled trial. Psychooncology. 2015;24(8):958–966.	13
Ferguson RJ, Ahles TA, Saykin AJ, et al. Cognitive-behavioral man- agement of chemotherapy-related cognitive change. Psychooncology. 2007;16(8):772-777.	14
Ercoli LM, Castellon SA, Hunter AM, et al. Assessment of the feasibility of a rehabilitation intervention program for breast cancer survivors with cognitive complaints. Brain Imaging Behav 2013;7:543-53.	15
Mihuta M, Green H, Shum D (2018) Efficacy of a web-based cog- nitive rehabilitation intervention for adult cancer survivors: a pilot study. Eur J Cancer Care 27(2):e12805	15
Milbury K, Chaoul A, Biegler K, et al. Tibetan sound meditation for cognitive dysfunction: results of a randomized controlled pilot trial. Psycho Oncol. 2013; 22(10):2354–2363. https://doi.org/10.1002/pon.3296.	15
Ferguson RJ, et al. Development of CBT for chemotherapy-related cognitive change: results of a waitlist control trial. Psychooncology 2012;21(2):176–86.	19
Von AD, Carpenter JS, Saykin A, Monahan P, Wu J, Yu M, et al. Advanced cognitive training for breast cancer survivors: a randomized controlled trial. Breast Cancer Res Treat. 2012;135(3):799– 809. https://doi.org/10.1007/s10549-012-2210-6.	19
Kesler S, et al. Cognitive training for improving executive function in chemotherapy- treated breast cancer survivors. Clin Breast Cancer 2013;13(4):299–306.	20
O'Neill, E. Guinan, S.L. Doyle, A.E. Bennett, C. Murphy, J.A. Elliott, J. O'Sullivan, J.V. Reynolds, J. Hussey, The RESTORE randomized controlled trial: impact of a multidisciplinary rehabilitative program on cardiorespiratory fitness in esophagogastric cancer survivorship, Ann. Surg. 268 (5) (2018) 747–755.	1
Naylor A.S.,C.Bull,M.K.L.Nilssonetal.,"Voluntary running rescues adult hippocampal neurogenesis after irradiation of the young mouse brain," Proceedings of the National Academy of Sciences of the United States of America, vol. 105, no. 38, pp. 14632–14637, 2008.	1
Akechi T, Momino K, Katsuki F, et al. Brief collaborative care intervention to reduce perceived unmet needs in highly distressed breast cancer patients: randomised controlled trial. Jpn J Clin Oncol. 2020;51(2):244-251. 10.1093/jjco/hyaa166	1
Ancoli-Israel S, Rissling M, Neikrug A, Trofimenko V, Natarajan L, Parker BA, Lawton S, Desan P, Liu L (2012) Light treatment prevents fatigue in women undergoing chemotherapy for breast cancer. Support Care Cancer 20(6):1211–1219	1
Andersen AH, Vinther A, Poulsen LL, Mellemgaard A. Do patients with lung cancer benefit from physical exercise? Acta Oncol (Madr). 2011;50(2):307-313. doi:10.3109/0284186X.2010.529461.	1
Arneil M, Anderson D, Alexander K, McCarthy A. Physical activ- ity and cognitive changes in younger women after breast cancer treatment. BMJ Support Palliat Care. 2020;10:122–5. https://doi. org/10.1136/bmjspcare-2019-001876	1
Arrieta H, Astrugue C, Regueme S, Durrieu J, Maillard A, Rieger A, et al. Effects of a physical activity programme to prevent physical performance decline in onco-geriatric patients: a randomized multicentre trial. J Cachexia Sarcopenia Muscle. 2019;10(2):287–297. doi: 10.1002/jcsm.12382.	1
Atkinson M, Murnane A, Goddard T, et al. A randomized controlled trial of a structured exercise intervention after the completion of acute cancer treatment in adolescents and young adults. Pediatr Blood Cancer 2021; 68: e28751.	1
Bai, L., Wang, X., Zhou, R., & Zeng, Q. (2020). Virtual cognitive rehabilitation training in transhepatic arterial infusion chemoembolization for liver cancer application in patients with mild cognitive impairment. Chinese Journal of Modern Nursing, 26(11), 1449–1454	1
Barrera M, Schulte F. A group social skills intervention program for survivors of childhood brain tumors. J Pediatr Psychol. 2009;34(10):1108–1118.	1
Bartolo M, Zucchella C, Pace A, et al. Early rehabilitation after surgery improves functional outcome in inpatients with brain tumours. J NeuroOncol 2012;107:537–44.	1
Baumann FT, Kraut L, Schule K, Bloch W, Fauser AA. A con- trolled randomized study examining the effects of exercise therapy on patients undergoing haematopoietic stem cell trans- plantation. Bone Marrow Transplant. 2010;45:355–62. https:// doi.org/10.1038/bmt.2009.163	1
Beaver K, Williamson S, Sutton C, et al. Comparing hospital and telephone follow-up for women treated for endometrial cancer (ENDCAT trial). BJOG 2017;124:150–60.	1

Berger AM, Kuhn BR, Farr LA, Lynch JC, Agrawal S, Chamberlain J, Von Essen SG (2009) Behavioral therapy intervention trial to improve sleep quality and cancer-related fatigue. Psycho-oncology 18(6):634–646	1
Bernstein LJ, McCreath GA, Nyhof-Young J, et al. A brief psychoe- ducational intervention improves memory contentment in breast cancer survivors with cognitive concerns: results of a single-arm prospective study. Support Care Cancer. 2018;26(8):2851–2859.	1
Björneklett HG, Rosenblad A, Lindemalm C, Ojutkangas ML, Letocha H, Strang P, et al. Long-term follow-up of a randomized study of support group intervention in women with primary breast cancer. J Psychosom Res2013;74:346–53.	1
Bland KA, Kirkham AA, Bovard J, et al. Effect of exercise on taxane chemotherapy-induced peripheral neuropathy in women with breast cancer: A randomized controlled trial. Clin Breast Cancer. 2019;19(6):411–22. https://doi.org/10.1016/j.clbc.2019.05.013. Medline:31601479	1
Bolam KA, Mijwel S, Rundqvist H, et al. Two-year follow-up of the OptiTrain randomised controlled exercise trial. Breast Cancer Res Treat. 2019;175(3):637–48. https://doi.org/10.1007/s10549-019- 05204-0. Medline:30915663	1
Bragard I, Etienne A, Faymonville M, Coucke P, Lifrange E, Schroeder H, et al. A nonrandomized comparison study of self- hypnosis, yoga, and cognitive-behavioral therapy to reduce emotional distress in breast cancer patients. Int J Clin Exp Hypn. 2017;65(2):189–209	1
Braun SE, Aslanzadeh FJ, Lanoye A, Fountain-Zaragoza S, Malkin MG, Loughan AR. Working memory training for adult glioma patients: a proof-of-concept study. J Neuro-Oncol. 2021;155:25-34. doi:10.1007/s11060-021-03839-y	1
Brown JC, Damjanov N, Courneya KS, et al. A randomized dose- response trial of aerobic exercise and health-related quality of life in colon cancer survivors. Psychooncology 2018;27:1221e1228.	1
Cantarero-Villanueva I, Fernández-Lao C, Díaz-Rodriguez L, Fernández-de-las-Peñas C, del Moral-Avila R, Arroyo-Morales M (2011) A multimodal exercise program and multimedia support reduce cancer-related fatigue in breast cancer survivors: a randomised controlled clinical trial. Eur J Integr Med 3(3):e189–e200	1
Cimprich B, So H, Ronis DL, Trask C. Pre-treatment factors related to cognitive functioning in women newly diagnosed with breast cancer. Psychooncology 70–8.	1
Colledge, F., Brand, S., Puhse, U., Holsboer-Trachsler, E., Zimmerer, S., Schleith, R., & Gerber, M. (2018). A twelve-week moderate exercise programme improved symptoms of depression, insomnia, and verbal learning in post-aneurysmal subarachnoid haemorrhage patients: A comparison with meningioma patients and healthy controls. Neuropsychobiology, 76(2), 59–71. https://doi.org/10.1159/00048 6903	1
Conklin HM, Ashford JM, Clark KN, Martin-Elbahesh K, Hardy KK, Merchant TE, et al. Long-term efficacy of computerized cognitive training among survivors of childhood cancer: A single-blind randomized controlled trial. J Pediatr Psychol 2017;42:220-31.	1
Cormie P, Galvão DA, Spry N, Joseph D, Chee R, Taaffe DR, et al. Can supervised exercise prevent treatment toxicity in patients with prostate cancer initiating androgen-deprivation therapy: a randomised controlled trial. BJU Int. 2015;115:256–66.	1
Cui Z, Dong H, Yu Z, Na L, Qing Z. Clinical study on qi-boosting and spirit regulating acupuncture on chemotherapy-induced mild cognitive impairment in breast cancer patients. Int J Clin Acupunct. 2018;27(4):222-227.	1
Devine KA, Viola A, Levonyan-Radloff K, et al. Feasibility of FitSurvivor: a technology-enhanced group-based fitness intervention for adolescent and young adult survivors of childhood cancer. Pediatr Blood Cancer 2020; 67: e28530	1
Dobos G, Overhamm T, Bussing A, et al. Integrating mindfulness in supportive cancer care: a cohort study on a mindfulness-based day care clinic for cancer survivors. Support Care Cancer. 2015;23(10):2945–55.	1
Doorenbos, A., Given, B., Given, C., Verbitsky, N., Cimprich, B., & McCorkle, R. (2005). Reducing symptom limitations: A cognitive behavioral intervention randomized trial. Psycho-Oncology, 14(7), 574–584.	1
Dubnov-Raz G, Azar M, Reuveny R, Katz U, Weintraub M, Constantini NW. Changes in fitness are associated with changes in body composition and bone health in children after cancer. Acta Paediatr 2015; 104: 1055–61.	1
Durà Mata MJ, Molleda Marzo M, Teixidor P, et al. Randomized controlled trial on the impact of cognitive telerehabilitation on cognition and quality of life in glioma patients. Ann Phys Rehabil Med. 2018;61:e271.	1
Egset, K. S., Weider, S., Stubberud, J., Hjemdal, O., Ruud, E., Hjort, M. A., Eilertsen, M.-E. B., Sund, A. M., Røkke, M. E., & Reinfjell, T. (2021). Cognitive rehabilitation for neurocognitive late effects in adult survivors of childhood acute lymphoblastic leukemia: A feasibility and case-series study. Frontiers in Psychology, 12, 1–12. https://doi.org/10.3389/fpsyg.2021. 724960.	1
Elnaggar RK, Mohamed RR. Aqua-plyometric exercises: potential implications for bone mineral density, functional capacity, and quality of life in survivors of childhood acute lymphoblastic leukemia. Semin Oncol Nurs 2021; 37: 151225.	1
F.T. Baumann, E.M. Zopf, E. Nykamp, L. Kraut, K. Schüle, T. Elter, A.A. Fauser, W. Bloch, Physical activity for patients undergoing an allogeneic hematopoietic stem cell transplantation: benefits of a moderate exercise intervention, Eur. J. Haematol. 87 (2) (2011) 148–156.	1
Fazzino TL, Klemp J, Befort C. Late breast cancer treatment- related symptoms and functioning: Associations with physical activity adoption and maintenance during a lifestyle intervention for rural survivors. Breast Cancer Res Treat. 2018;168:755–61. https://doi.org/10.1007/s10549-017-4603-z	1
Feng L, Yang D. Observation on the effect of high-quality nursing intervention plus health education in chemotherapy for non-small cell lung cancer and its influence on the physical and mental health of patients. Evid Based Complement Altern Med. 2022;2022:1-8. 10.1155/2022/2459013	1
Fontana A, Matthey S, Mayor C, Dufour C, Destaillats A, Balla- beni P, Maeder S, Newman CJ, Beck Popovic M, Renella R, Diezi M. Pastec - a prospective, single-center, randomized, cross-over trial of pure physical versus physical plus attentional training in children with cancer. J Pediatr Hematol Oncol. 2022;39:329–42. https://doi.org/10.1080/08880018.2021.1994677	1
Fukushima T, Nakano J, Ishii S, et al. Low-intensity exercise therapy with high frequency improves physical function and mental and physical symptoms in patients with haematological malignancies undergoing chemotherapy. Eur J Cancer Care (Engl). 2018;27(6): e12922 . https://doi.org/10.1111/ecc.12922 . Medline:30311313	1
G. Winocur, J. M. Wojtowicz, J. Huang, and I. F. Tannock, “Physical exercise prevents suppression of hippocampal neu- rogenesis and reduces cognitive impairment in chemotherapy- treated rats,” Psychopharmacology, vol. 231, no. 11, pp. 2311–2320, 2014.	1
Galantino ML, Cannon N, Hoelker T, Quinn L, Greene L. Effects of iyengar yoga on measures of cognition, fatigue, quality of life, flexibility, and balance in breast cancer survivors: a case series. Rehabil Oncol. 2008;26:18–27.	1
Gehring, K., Taphoorn, M. J., Sitskoorn, M. M., & Aaronson, N. K. (2015). Predictors of subjective versus objective cognitive functioning in pa- tients with stable grades II and III glioma. Neuro-Oncology Practice, 2(1), 20–31. https://doi.org/10.1093/nop/npu035	1
Gielissen, M. F., Verhagen, S., Witjes, F., & Bleijenberg, G. (2006). Effects of cognitive behavior therapy in severely fatigued disease-free cancer patients compared with patients waiting for cognitive behavior therapy: a randomized controlled trial. Journal of Clinical Oncology, 24(30), 4882-4887.	1
Gielissen, M. F., Wiborg, J. F., Verhagen, C. A., Knoop, H., & Bleijenberg, G. (2012). Examining the role of physical activity in reducing postcancer fatigue. Supportive Care in Cancer, 20(7), 1441-1447.	1

Given, C. W., Sikorskii, A., Tamkus, D., Given, B., You, M., Mc- Corkle, R., . . . Decker, D. (2008). Managing symptoms among patients with breast cancer during chemotherapy: Results of a two-arm behavioral trial. Journal of Clinical Oncology, 26(36), 5855–5862.	1
Given, C., Given, B., Rahbar, M., Jeon, S., McCorkle, R., Cimprich, B., . . . Bowie, E. (2004). Effect of a cognitive behavioral inter- vention on reducing symptom severity during chemotherapy. Journal of Clinical Oncology, 22(3), 507–516.	1
Gjerset GM, Skaali T, Seland M, Thorsen L. Health-related quality of life, fatigue, level of physical activity and physical capacity before and after an outpatient rehabilitation program for women within working age treated for breast cancer. J Cancer Educ. 2022. 10.1007/s13187-022-02211-6	1
Goedendorp, M. M., Gielissen, M. F., Peters, M. E., Verhagen, C. A., & Bleijenberg, G. (2012). Moderators and long-term effectiveness of cognitive behaviour therapy for fatigue during cancer treatment. Psycho-Oncology, 21(8), 877-885.	1
Götte M, Kesting SV, Gerss J, Rosenbaum D, Boos J. Feasibility and effects of a home-based intervention using activity trackers on achievement of individual goals, quality of life and motor performance in patients with paediatric cancer.<br>BMJ Open Sport Exerc Med 2018; 4: e000322.	1
Greenberg E, Treger I, Ring H. Rehabilitation outcomes in patients with brain tumors and acute stroke: comparative study of inpatient rehabilitation. Am J Phys Med Rehab 2006;85:568–73	1
Gregoire C, et al. Effects of an intervention combining self-care and self-hypnosis on fatigue and associated symptoms in post- treatment cancer patients: a randomized-controlled trial. Psy- chooncology. 2020;29(7):1165–73.	1
Grenawalt TA, Tansey TN, Phillips BN, Strauser DR, Rosenthal DA, Wagner S. Effectiveness of internet-based behavioral activation on quality of life among young adult survivors of childhood brain tumor: a randomized controlled trial. Disabil Rehabil (2022), 1–8. doi: 10.1080/09638288.2022.2094478	1
Hartman SJ, Nelson SH, Marinac CR, Natarajan L, Parker BA, Patterson<br>RE. The effects of weight loss and metformin on cognition among<br>breast cancer survivors: Evidence from the Reach for Health study. Psychooncology<br>2019;28:1640–6.	1
Hartman SJ, Weiner LS, Nelson SH, Natarajan L, Patterson RE, Palmer BW, Parker BA, Sears DD. Mediators of a physical activity intervention on cognition in breast cancer survivors: evidence from a randomized controlled trial. JMIR Cancer. 2019;5:e13150. https://doi.org/10.2196/13150	1
Hawkes AL, Chambers SK, Pakenham KI, et al. Effects of a telephone-delivered multiple health behavior change intervention (CanChange) on health and behavioral outcomes in survivors of colo- rectal cancer: A randomized controlled trial. J Clin Oncol 2013;31: 2313e2321.	1
Henneghan AM, Becker H, Phillips C, Kesler S. Sustained effects of mantra meditation compared to music listening on neurocognitive outcomes of breast cancer survivors: a brief report of a randomised controlled trial. J Psychosom Res. 2021;150:110628. 10.1016//j.jpsychores.2021.110628	1
Henneghan AM, Fico BG, Wright ML, et al. Effects of meditation compared to music listening on biomarkers in breast cancer survi- vors with cognitive complaints: secondary outcomes of a pilot randomized control trial. Explore. 2022;18(6):657–662.	1
Hocking MC, Schwartz LA, Hobbie WL, Derosa BW, Ittenbach RF, Mao JJ, Ginsberg JP, Kazak AE. Prospectively examining physical activity in young adult survivors of childhood cancer and healthy controls. Pediatr Blood Cancer. 2013;60:309–15. https://doi.org/10.1002/pbc.24144	1
Hoffman CJ, Ersser SJ, Hopkinson JB, Nicholls PG, Harrington JE, Thomas PW. Effectiveness of mindfulness-based stress reduction in mood, breast- and endocrine-related quality of life, and well-being in stage 0 to III breast cancer: A randomized, controlled trial. J Clin Oncol 1335–42.	1
Hojan K, Gerreth K. Can multidisciplinary inpatient and out- patient rehabilitation provide sufficient prevention of disability in patients with a brain tumor?-a Case-series report of two pro- grams and a prospective, observational clinical trial. Int J Environ Res Public Health. 2020;17:6488. doi:10.3390/ijerph17186488	1
Hsieh CC, Sprod L, Hydock DS, Carter SD, Hayward R, Schneider CM. Effects of a supervised exercise intervention on recovery from treatment regimens in breast cancer survivors. Oncol Nurs Forum. 2008;35:909–915.	1
Huang CC, Kuo HP, Lin YE, Chen SE. Effects of a web-based health education program on quality of life and symptom distress of initially diagnosed advanced non-small cell lung cancer patients: a randomised controlled trial. J Cancer Educ. 2019;34(1):41-49. 10.1007/s13187-017-1263-y	1
Hulshof MCCM, Stark NM, van der Kleij A, Sminia P, Smeding HMM, Gonzalez Gonzalez D. Hyperbaric oxygen therapy for cognitive disorders after irradiation of the brain. Strahlenther Onkol. 2002;178:192-198. doi:10.1007/s00066-002-0916-9	1
J. E. Fardell, J. Vardy, J. D. Shah, and I. N. Johnston, “Cognitive impairments caused by oxaliplatin and 5-fluorouracil chemotherapy are ameliorated by physical activity,” Psychophar- macology, vol. 220, no. 1, pp. 183–193, 2012.	1
J.A. Ligibel, A. Giobbie-Hurder, L. Shockro, N. Campbell, A.H. Partridge, S. M. Tolaney, N.U. Lin, E.P. Winer, Randomized trial of a physical activity intervention in women with metastatic breast cancer, Cancer 122 (8) (2016) 1169–1177.	1
J.-F. Ji, S.-J. Ji, R. Sun et al., “Forced running exercise attenuates hippocampal neurogenesis impairment and the neurocognitive deficits induced by whole-brain irradiation via the BDNF- mediated pathway,” Biochemical and Biophysical Research Com- munications, vol. 443, no. 2, pp. 646–651, 2014.	1
J.H. Do, W. Kim, Y.K. Cho, J. Lee, E.J. Song, Y.M. Chun, J.Y. Jeon, Effects OF resistance exercises and complex decongestive therapy ON arm function and muscular strength IN breast cancer related lymphedema, Lymphology 48 (4) (2015) 184–196.	1
Jarden M, Baadsgaard MT, Hovgaard DJ, Boesen E, Adamsen L. A randomized trial on the effect of a multimodal intervention on physical capacity, functional performance and quality of life in adult patients undergoing allogeneic SCT. Bone Marrow Transplant 2009;43(9):725–37.	1
Jarden M, Nelausen K, Hovgaard D, Boesen E, Adamsen L. The effect of a multimodal intervention on treatment-related symptoms in patients undergoing hematopoietic stem cell transplantation: a randomized controlled trial. J Pain Symptom Manage. 2009;38:174–190.	1
Jefford M, Gough K, Drosdowsky A, et al. A randomized controlled trial of a nurse-led supportive care package (SurvivorCare) for survivors of colorectal cancer. Oncologist 2016;21:1014–23	1
Johnston MF, Hays RD, Subramanian SK, Elashoff RM, Axe EK, Li JJ, et al. Patient education integrated with acupuncture for relief of cancer-related fatigue randomized controlled feasibility study. BMC Complement Altern Med 49.	1
Jung M, Wallek S, Senn-Malashonak A, et al. Effects of a structured exercise therapy on cancer-related fatigue during and after paediatric stem cell transplantation: a randomized controlled trial. Physiotherapy Quarterly 2021; 29: 76–85.	1
K. M. Mustian, M. C. Janelsins, L. J. Peppone et al., “EXCAP exercise effects on cognitive impairment and inflammation: a URCC NCORP RCT in 479 cancer patients,” Journal of Clinical Oncology, vol. 2015, no. 15, supplement 9504, 2015.	1
Kampshoff CS, Chinapaw MJM, Brug J, Twisk JWR, Schep G, Nijziel MR, et al. Randomized controlled trial of the effects of high intensity and low-to-moderate intensity exercise on physical fitness and fatigue in cancer survivors: results of the Resistance and Endurance exercise After ChemoTherapy (REACT) study. BMC Med. 2015;13:275.	1
Khodashenas E, Badiee Z, Sohrabi M, Ghassemi A, Hosseinzade V. The effect of an aerobic exercise program on the quality of life in children with cancer. Turk J Pediatr 2017; 59: 678–83.	1

Kiecolt-Glaser JK, Bennett JM, Andridge R, Peng J, Shapiro CL, Malarkey WB, Emery CF, Layman R, Mrozek EE, Glaser R (2014) Yoga's impact on inflammation, mood, and fatigue in breast cancer survivors: a randomized controlled trial. J Clin Oncol 32(10):1040– 1049	1
Kim YH, Choi KS, Han K, et al. A psychological intervention program for patients with breast cancer under chemotherapy and at a high risk of depression: a Randomized Clinical Trial. JCN 2018;27:572–81.	1
Kimman ML, Dirksen CD, Voogd AC, et al. Nurse-led telephone follow- up and an educational group programme after breast cancer treatment: results of a 2 x 2 randomised controlled trial. Eur J Cancer 2011;47:1027–36.	1
Knoerl R, Giobbie-Hurder A, Sannes TS, Chagpar AB, Dil- lon D, Dominici LS, Frank ES, Golshan M, McTiernan A, Rhei E, Tolaney SM, Winer EP, Yung RL, Irwin ML, Ligibel JA. Exploring the impact of exercise and mind-body prehabilita- tion interventions on physical and psychological outcomes in women undergoing breast cancer surgery. Support Care Cancer. 2022;30:2027–36. https://doi.org/10.1007/s00520-021-06617-8	1
Korstjens I, Mesters I, May AM, van Weert E, van den Hout JH, Ros W, Hoekstra-Weebers JE, van der Schans CP, van den Borne B. Effects of cancer rehabilitation on problem-solving, anxiety and depression: a rct comparing physical and cognitive-behavioural training versus physical training. Psychol Health. 2011;26(Suppl 1):63–82. https://doi.org/10.1080/08870441003611569	1
L.M. Ercoli, L. Petersen, A.M. Hunter, S.A. Castellon, L. Kwan, B.A. Kahn-Mills, et al. Cognitive rehabilitation group intervention for breast cancer survivors: results of a randomized clinical trial Psychooncology, 24 (2015), pp. 1360-1367	1
Lambert M, Wurz A, Smith AM, Fang Z, Brunet J. Preliminary evidence of improvement in adolescent and young adult can- cer survivors' brain health following physical activity: a proof- of-concept sub-study. Brain Plast. 2021;7:97–109. https://doi. org/10.3233/BPL-210124	1
Leclerc AF, Slomian J, Jerusalem G, et al. Exercise and education program after breast cancer: benefits on quality of life and symp- toms at 3, 6, 12, and 24 months' follow-up. Clin Breast Cancer. 2018;18(5):e1189–e1204.	1
Lee, H., Lim, Y., Yoo, M. S., & Kim, Y. (2011). Effects of a nurse-led cognitive-behavior therapy on fatigue and quality of life of patients with breast cancer undergoing radiotherapy: an exploratory study. Cancer nursing, 34(6), E22-E30.	1
Lerman R, Jarski R, Rea H, Gellish R, Vicini F. Improving symptoms and quality of life of female cancer survivors: a randomized controlled study. Ann Surg Oncol. 2012;19(2):373–8.	1
Lesiuk T. The effect of mindfulness-based music therapy on attention and mood in women receiving adjuvant chemotherapy for breast cancer: A pilot study. Oncol rs Forum 276–82.	1
Li HCW, Chung OKJ, Ho KY, Chiu SY, Lopez V. Effectiveness of an integrated adventure-based training and health education program in promoting regular physical activity among childhood cancer survivors. Psychooncology 2013; 22: 2601–10.	1
Li WHC, Ho KY, Lam KKW, et al. Adventure-based training to promote physical activity and reduce fatigue among childhood cancer survivors: a randomized controlled trial. Int J Nurs Stud 2018; 83: 65–74.	1
Li Z, Hao X, Lei P, et al. Patients with breast cancer receiving chemotherapy: effects of multisensory stimulation training on cognitive impairment. Clin J Oncol Nurs. 2022;26(1):71-78. 10.1188/22.CJON.71-7	1
Lin H, Zhou S, Zhang D, et al. Evaluation of a nurse-led management program to complement the treatment of adolescent acute lymphoblastic leukemia patients. Appl Nurs Res 2016;32:e1–5.	1
Lin KY, Shun SC, Lai YH, et al. Comparison of the ef- fects of a supervised exercise program and usual care in Patients with colorectal cancer undergoing chemo- therapy. Cancer Nurs 2014;37:E21eE29.	1
Lin ZG, Li RD, Ai FL, Li S, Zhang XA. Effects of cognitive behavioural therapy combined with Baduanjin in patients with colorectal cancer. World J Gastrointest Oncol. 2022;13(5):2647-2653. 10.4251/wjgo.v14.il.319	1
Lina Y, Yaping Y. Influence of nursing intervention on quality of life of patients with lung cancer. J North Pharm 2012;9:116-7.	1
Liou KT, et al. Effects of acupuncture versus cognitive behavioral therapy on cognitive function in cancer survivors with insom- nia: a secondary analysis of a randomized clinical trial. Cancer. 2020;126(13):3042–52.	1
Liu H, Zhao Y, Zhan H, et al. Influence of implementation of continuous health education on recent quality of life and physiological indexes of gastric cancer patients after total gastrectomy. Chinese Nursing Research 2013;27:1262-4.	1
Liu Z. Effect of health education on postoperative psychological status and quality of life of patients with gastric cancer. International Journal of Nursing ;33:618-20.	1
Livingston PM, Craike MJ, Salmon J, Courneya KS, Gaskin CJ, Fraser SF, Mohebbi M, Broadbent S, Botti M, Kent B, Group EU-OC. Effects of a clinician referral and exercise program for men who have completed active treatment for prostate cancer: a multicenter cluster randomized controlled trial (engage). Cancer. 2015;121:2646–54. https://doi.org/10.1002/cncr.29385	1
Lotzke D, Wiedemann F, Rodrigues Recchia D, Ostermann T, Sattler D, Ettl J, et al. Iyengar-yoga compared to exercise as a therapeutic intervention during (neo)adjuvant therapy in women with stage I-III breast cancer: health-related quality of life, mind- fulness, spirituality, life satisfaction, and cancer-related fatigue. Evid Based Complement Alternat Med. 2016;2016:5931816.	1
Loubani K, Kizony R, Milman U, et al. Hybrid tele and in-clinic occupation based intervention to improve women's daily participation after breast cancer: a pilot randomized controlled trial. Int J Environ Res Public Health. 2021;18:5966.	1
Lv M. Influence of health education and psychological nursing on the quality of life of patients underwent laparoscopic radical gastrostomy. Clinical Research and Practice 2016;1:152-3.	1
Mackenzie MJ, Carlson LE, Paskevich DM, Ekkekakis P, Wurz AJ, Wytsma K, et al. Associations between attention, affect and cardiac activity in a single yoga session for female cancer survivors: an enactive neurophenomenology-based approach. Conscious Cogn. 2014;27:129–46.	1
Malmström M, Ivarsson B, Klefsgård R, et al. The effect of a nurse led telephone supportive care programme on patients' quality of life, received information and health care contacts after oesophageal cancer surgery—a six month RCT-follow-up study. Int J Nurs Stud 2016;64:86–95.	1
Marciniak CM, Sliwa JA, Heinemann AW, et al. Functional outcomes of persons with brain tumors after inpatient rehabilitation. Arch Phys Med Rehab 2001;82:457–63	1
Marinac CR, Godbole S, Kerr J, Natarajan L, Patterson RF, Hartman SJ. Objectively measured physical activity activity and cognitive functioning in breast cancer survivors. Journal of Cancer Survivorship. 2015;9:230–238.	1
Mehnert A, Veers S, Howaldt D, Braumann K-M, Koch U, Schulz K-H. Effects of a physical exercise rehabilitation group program on anxiety, depression, body image, and health-related quality of life among breast cancer patients. Oncol Res Treat. 2011;34:248–53.	1
Mendoza JA, Baker KS, Moreno MA, et al. A Fitbit and Facebook mHealth intervention for promoting physical activity among adolescent and young adult childhood cancer survivors: a pilot study. Pediatr Blood Cancer 2017; 64: e26660.	1
Mihuta ME, Green HJ, Shum DHK. Web-based cognitive rehabilitation for survivors of adult cancer: a randomised controlled trial. Psychooncology. (2018) 27:1172–9. doi: 10.1002/pon.4615	1

Mijwel S, Jervaeus A, Bolam KA, et al. High-intensity exercise during chemotherapy induces beneficial effects 12 months into breast cancer survivorship. J Cancer Surviv. 2019;13(2):244–56. https://doi. org/10.1007/s11764-019-00747-z. Medline:30912010	1
Min L, Aifang X, Yuanling M. Effect of psychological intervention on quality of life of patients with lung cancer chemotherapy. Today Nurse 2009;16:5-6.	1
Morielli AR, Boule NG, Usmani N, Tankel K, Joseph K, Severin D, Fairchild A, Nijjar T, Courneya KS. Effects of exercise during and after neoadjuvant chemoradiation on symptom burden and quality of life in rectal cancer patients: a phase II randomized controlled trial. J Cancer Surviv. 2021;17:1171–83. https://doi. org/10.1007/s11764-021-01149-w	1
Nusca SM, Parisi A, Mercantini P, Gasparrini M, Pitasi FA, Lacopo A, Colonna V, Stella G, Cerulli C, Grazioli E, Tranchita E, Santoboni F, Latini E, Trischitta D, Vetrano M, Visco V, Pavan A, Vulpiani MC. Evaluation of a post-operative rehabili- tation program in patients undergoing laparoscopic colorectal cancer surgery: a pilot study. Int J Environ Res Public Health. 2021;18:5632. https://doi.org/10.3390/ijerph18115632	1
Oechsle K, Jensen W, Schmidt T, et al. Physical activity, quality of life, and the interest in physical exercise programs in patients undergoing palliative chemotherapy. Support Care Cancer. 2011; 19(5):613–19. https://doi.org/10.1007/s00520-010-0862-5. Medline:20352266	1
Park JH, Park KD, Kim JH, Kim YS, Kim EY, Ahn HK, Park I, Sym SJ. Resistance and aerobic exercise intervention during chemotherapy in patients with metastatic cancer: a pilot study in south korea. Ann Palliat Med. 2021;10:10236–43. https://doi. org/10.21037/apm-21-1432	1
Persoon S, ChinAPaw MJM, Buffart LM, Liu RDK, Wijermans P, Koene HR, et al. Randomized controlled trial on the effects of a supervised high intensity exercise program in patients with a hematologic malignancy treated with autologous stem cell transplantation: Results from the EXIST study. PloS One. 2017;12:e0181313.	1
Peterson BM, Shackelford DYK, Brown JM, Brennecke AP, Hayward R. Effects of aerobic and flexibility training on physio- logical and psychosocial function in a patient with anaplastic oli- godendroglioma: a case report. J Rehabil Med. 2020;3:1000032. https://doi.org/10.2340/20030711-1000032	1
Phillips SM, Lloyd GR, Awick EA, McAuley E. Relationship between self-reported and objectively measured physical activity and subjective memory impairment in breast cancer survivors: role of self-efficacy, fatigue and distress. Psycho-Oncol. 2017;26:1390–9. https://doi.org/10.1002/pon.4156	1
Poggi G, Liscio M, Pastore V, Adduci A, Galbiati S, Spreafico F, et al. Psychological intervention in young brain tumor survivors: the efficacy of the cognitive behavioural approach. Disabil Rehabil (2009) 31(13):1066–73. doi: 10.1080/ 09638280802509546	1
Poier D, Bussing A, Rodrigues Recchia D, Beerenbrock Y, Reif M, Nikolaou A, Zerm R, Gutenbrunner C, Kroz M. Influ- ence of a multimodal and multimodal-aerobic therapy con- cept on health-related quality of life in breast cancer survivors. Integr Cancer Ther. 2019;18:1534735418820447. https://doi. org/10.1177/1534735418820447	1
Pyszora A, Budzynski J, Wojcik A, et al. Physiotherapy programme reduces fatigue in patients with advanced cancer receiving palliative care: randomized controlled trial. Support Care Cancer 2017;25: 2899e2908.	1
Rao SM, Bieliauskas LA. Cognitive rehabilitation two and one-half years post right temporal lobectomy. J Clin Neuropsychol 1983;5: 313-20.	1
Reilly Julia M, Gundersen Alexandra I, Silver Julie K, et al. A comparison of functional outcomes between patients admitted to inpatient rehabilitation after initial diagnosis versus recurrence of glioblastoma multiforme. PM R 2020;10:975–83.	1
Roberts Pamela S, Nuño M, Sherman D, et al. The impact of inpatient rehabilitation on function and survival of newly diagnosed patients with glioblastoma. Pm&R 2014;6:514–21	1
Rogers LQ, Hopkins-Price P, Vicari S, Markwell S, Pamenter R, Courneya KS, Hoelzer K, Naritoku C, Edson B, Jones L, Dun- nington G, Verhulst S. Physical activity and health outcomes three months after completing a physical activity behavior change intervention: persistent and delayed effects. Cancer Epidemiol Biomarkers Prev. 2009;18:1410–8. https://doi.org/10.1158/1055- 9965.EPI-08-1045	1
Rongxiang X. Influence of nursing intervention on quality of life of patients with lung cancer. J Qilu Nurs 2011;12:33-4.	1
Rosero ID, Ramirez-Velez R, Martinez-Velilla N, Cedeno-Veloz BA, Morilla I, Izquierdo M. Effects of a multicomponent exercise program in older adults with non-small-cell lung cancer during adjuvant/palliative treatment: an intervention study. J Clin Med. 2020;9:862. https://doi.org/10.3390/jcm9030862	1
Rueegg CS, Kriemler S, Zuercher SJ, et al. A partially supervised physical activity program for adult and adolescent survivors of childhood cancer (SURfit): study design of a randomized controlled trial [NCT02730767]. BMC Cancer 2017; 17: 822.	1
S. J. E. Wong-Goodrich, M. L. Pfau, C. T. Flores, J. A. Fraser, C. L. Williams, and L. W. Jones, "Voluntary running prevents progressive memory decline and increases adult hippocampal neurogenesis and growth factor expression after whole-brain irradiation," Cancer Research, vol. 70, no. 22, pp. 9329–9338, 2010.	1
Sacks-Zimmerman A, Duggal D, Liberta T. Cognitive remedia- tion therapy for brain tumor survivors with cognitive deficits. Cureus. 2015;7:e350. doi:10.7759/cureus.350	1
Şahin S, Akel BS, Huri M, Akyüz C. Investigation of the effect of task-orientated rehabilitation program on motor skills of children with childhood cancer: a randomized-controlled trial. Int J Rehabil Res 2020; 43: 167–74.	1
Salchow J, Koch B, Mann J, et al. Effects of a structured counselling- based intervention to improve physical activity behaviour of adolescents and young adult cancer survivors—the randomised phase II Motivate AYA—MAYA trial. Clin Rehabil 2021; 35: 1164–74.	1
Sarvghadi P, Ghaffari A, Rostami HR. The effects of neurofeedback training on short-term memory and quality of life in women with breast cancer. Int J Ther Rehabil. 2019;26:1–8.	1
Schellart NAM, Reits D, van der Kleij AJ, Stalpers LJA. Hyperbaric oxygen treatment improved neurophysiologic performance in brain tumor patients after neurosurgery and ra- diotherapy: a preliminary report. Cancer. 2011;117:3434-3444. doi:10.1002/cncr.25874	1
Sheehan P, Denieffe S, Murphy NM, Harrison M. Exercise is more effective than health education in reducing fatigue in fatigued cancer survivors. Support Care Cancer 2020;28(10):4953–62.	1
Simeit R, Deck R, Conta-Marx B. Sleep management train- ing for cancer patients with insomnia. Support Care Cancer. 2004;12(3):176–83.	1
Speca M, Carlson LE, Goodey E, Angen M. A randomized, wait-list controlled clinical trial: The effect of a mindfulness meditation-based stress reduction program on mood and symptoms of stress in cancer outpatients. Psychosom Med 613–22.	1
Spreafico F, Barretta F, Murelli M, Chisari M, Gattuso G, Teren- ziani M, Ferrari A, Veneroni L, Meazza C, Massimino M. Posi- tive impact of organized physical exercise on quality of life and fatigue in children and adolescents with cancer. Front Pediatr. 2021;9:627876. https://doi.org/10.3389/fped.2021.627876	1
Stacey, F. G., James, E. L., Chapman, K., & Lubans, D. R. (2016). Social cognitive theory mediators of physical activity in a lifestyle program for cancer survivors and carers: findings from the ENRICH randomized controlled trial. International Journal of Behavioral Nutrition and Physical Activity, 13(1), 1-13.	1
T.R.S. Paulo, F.E. Rossi, J. Viezel, G.T. Tosello, S.C. Seidinger, R.R. Simõ es, R. de Freitas Jr., I.F. Freitas Jr., The impact of an exercise program on quality of life in older breast cancer survivors undergoing aromatase inhibitor therapy: a randomized controlled trial, Health Qual. Life Outcome 17 (1) (2019) 17.	1

Taaffe DR, Newton RU, Spry N, Joseph D, Chambers SK, Gardiner RA, et al. Effects of different exercise modalities on fatigue in prostate cancer patients undergoing androgen deprivation therapy: a year-long randomised controlled trial. Eur Urol. 2017;72:293–9.	1
Tack L, Lefebvre T, Lycke M, et al. A randomised waitlist controlled trial to evaluate Emotional Freedom Techniques for self-report cancer-related cognitive impairment in cancer survivors (EMOTICON). E Clin Med. 2021;39:101081. 10.1016/j.eclinm.2021.101081	1
Tan TT. Effect of multi-sensory stimulation training on cognitive impairment and depression in patients with breast cancer chemotherapy. North China University of Science and Technology, 2019.	1
Tan, T., Zhou, L., Chen, C., Fu, L., & Cui, Z. (2020). Intervention effect of multisensory training on memory impairment of breast cancer patients during chemotherapy. Chinese Journal of Physi- cal Medicine and Rehabilitation, 42(9), 836–838. [in Chinese].	1
Tanir MK, Kuguoglu S. Impact of exercise on lower activity levels in children with acute lymphoblastic leukemia: a randomized controlled trial from Turkey. Rehabil Nurs 2013; 38: 48–59.	1
Taylor J, Weyer-Jamora C, Brie M, et al. NCOG-21. Interim re- sults of three cognitive rehabilitation strategies in patients with lower grade gliomas. Neuro-Oncology. 2020;22:ii133-ii134.	1
Thorsen L, Skovlund E, Strømme SB, Hornslien K, Dahl AA, Fosså SD. Effectiveness of physical activity on cardiorespiratory fitness and health-related quality of life in young and middle- aged cancer patients shortly after chemotherapy. J Clin Oncol. 2005;23:2378–88.	1
Travier N, Velthuis MJ, Steins Bisschop CN, van den Buijs B, Monninkhof EM, Backx F, et al. Effects of an 18-week exercise programme started early during breast cancer treatment: a randomised controlled trial. BMC Med. BMC Med. 2015;13:121.	1
Trinh L, Kramer AF, Rowland K, Strom DA, Wong JN, McAuley E. A pilot feasibility randomized controlled trial adding behav- ioral counseling to supervised physical activity in prostate can- cer survivors: Behavior change in prostate cancer survivors trial (boost). J Behav Med. 2021;44:172–86. https://doi.org/10.1007/ s10865-020-00185-8	1
van den Berg M, Visser A, Schoolmeesters A, Edelman P, van den Borne B. Evaluation of haptotherapy for patients with cancer treated with chemotherapy at a day clinic. Patient Educ Couns 336–43.	1
Van Der Lee, M. L., & Garssen, B. (2012). Mindfulness-based cognitive therapy reduces chronic cancer-related fatigue: a treatment study. Psycho-Oncology, 21(3), 264-272.	1
Vaquero L, Rodriguez-Fornells A, Pera-Jambrina MA, Bruna J, Simo M. Plasticity in bilateral hippocampi after a 3-month physical activity programme in lung cancer patients. Eur J Neurol. 2021;28:1324–33. https://doi.org/10.1111/ene.14670	1
Varni JW, Katz ER, Colegrove R Jr, et al. The impact of social skills train- ing on the adjustment of children with newly diagnosed cancer. J Pediatr Psychol. 1993;18(6):751–767.	1
Verschuur EML, Steyerberg EW, Tilanus HW, et al. Nurse-led follow-up of patients after oesophageal or gastric cardia cancer surgery: a randomised trial. Br J Cancer 2009;100:70–6.	1
Von Ah D, McDonald BC, Crouch AD, et al. Randomized double-masked controlled trial of cognitive training in breast cancer survivors: a preliminary study. Support Care Cancer 2022;30:7457–67	1
Weissflog G, Brahler E, Leuteritz K, Barthel Y, Kuhnt S, Wiltink J, Zwerenz R, Beutel ME (2015) Does psychodynamic short-term psychotherapy for depressed breast cancer patients also improve fatigue? Results from a randomized controlled trial. Breast Cancer Res Treat 152(3):581–588	1
Wen L. Effect of physical and mental rehabilitation exercise on cancer-related fatigue in patients with colon cancer after chemo- therapy. Bipedal and Health Care 2018;2:108e110.	1
Winters-Stone KM, Dobek JC, Bennett JA, Dieckmann NF, Maddalozzo GF, Ryan CW, et al. Resistance training reduces disability in prostate cancer survivors on androgen deprivation therapy: evidence from a randomized controlled trial. Arch Phys Med Rehabil. 2015;96:7–14.	1
Wolf TJ, Doherty M, Kallogjeri D, et al. The feasibility of using metacognitive strategy training to improve cognitive performance and neural connectivity in women with chemotherapy-induced cognitive impairment. Oncology. 2016;91(3):143–152.	1
Wu H. Influence of Health Education Intervention on Cancer-related Fatigue and Quality of Life in Gastric Cancer Patients. Nursing Journal of Chinese People Liberation Army 2008;25:9-11	1
Wurz A, Ayson G, Smith AM, Brunet J. A proof-of-concept sub- study exploring feasibility and preliminary evidence for the role of physical activity on neural activity during executive function- ing tasks among young adults after cancer treatment. BMC Neu- rol. 2021;21:300. https://doi.org/10.1186/s12883-021-02280-y	1
Xie J, Zhu T, Lu Q, Xu X, Cai Y, Xu Z. The effects of add-on self-care education on quality of life and fatigue in gastrointestinal cancer patients undergoing chemotherapy. J Altern Complement Med. 2020;20(15):1-8. 10.1186/s12906-019-2800-5	1
Xu G, Liu W, Wang C, et al. Hong Effect of aerobic exercise on can- cer related fatigue and quality of life of patients with post- operative adjuvant chemotherapy for colorectal cancer. Modern Oncol 2016; 24:2259e2261.	1
Xu L, Xiao J, Xu P. Effects of humanistic nursing mode combined with situational experiential health education on negative psychology, treatment compliance and quality of life of patients undergoing radical gastrectomy. World Chinese Journal of Digestion 2018;26:729-34.	1
Yeh CH, Man Wai JP, Lin US, Chiang YC. A pilot study to examine the feasibility and effects of a home-based aerobic program on reducing fatigue in children with acute lymphoblastic leukemia. Cancer Nurs 2011; 34: 3–12.	1
Yoon J, Chun MH, Lee SJ, Kim BR. Effect of Virtual Reality–Based Rehabilitation on Upper- Extremity Function in Patients with Brain Tumor: Controlled Trial. American Journal of Physical Medicine & Rehabilitation. 2015;94(6):449-459. doi:1 0.1097/phm.0000000000000192	1
Yu J, Jung Y, Park J, et al. Intensive rehabilitation therapy fol- lowing brain tumor surgery: a pilot study of effectiveness and long-term satisfaction. Ann Rehabil Med. 2019;43:129-141. doi:10.5535/arm.2019.43.2.129	1
Zhang B, Dong JN, Sun P, Feng C, Liu YC (2017) Effect of therapeutic care for treating fatigue in patients with breast cancer receiving chemotherapy. Medicine 96(33):e7750	1
Zhang ZJ, Man SC, Yam LL, et al. Electroacupuncture trigeminal nerve stimulation plus body acupuncture for chemotherapy-induced cognitive impairment in breast cancer patients: an assessor-participant blinded, randomised controlled trial. Brain Behav Immun. 2020;88:88-96. 10.1016/j.bbi.2020.04.035	1
Zhang, X., Yang, L., Hou, L., Liu, J., Zhu, H., & Zhang, J. (2020). Effect of a psychological nursing intervention on quality of life and cognitive function in patients with gastric carcinoma: A randomised controlled trial. European Journal of Cancer Care, 29(6), e13292. https://doi.org/10.1111/ecc.13292	1
ZhangQ,LiF,ZhangH,etal.Effectsofnurse-ledhome-basedexercise & cognitive behavioral therapy on reducing cancer-related fatigue in patients with ovarian cancer during and after chemotherapy: A randomized controlled trial. Int J Nurs Stud. 2018;78:52–60. https:// doi.org/10.1016/j.ijnurstu.2017.08.010. Medline:28939343	1

Zhao X, Gao Q, Wang Z. Effect of walking exercise on cancer- related fatigue and sleep quality in patients with colorectal cancer af- ter operation. Guangxi Med J 2018;40:723e725.	1
Zhuang S, Zhai X. The effect of early home-based aerobic exercise on colon cancer patients with cancer-related fatigue receiving chemotherapy. Tianjin J Nurs 2012;20:65e67.	1
Zimmer P, Baumann FT, Oberste M, Schmitt J, Joisten N, Har- tig P, Schenk A, Kuhn R, Bloch W, Reuss-Borst M. Influence of personalized exercise recommendations during rehabilitation on the sustainability of objectively measured physical activity levels, fatigue, and fatigue-related biomarkers in patients with breast cancer. Integr Cancer Ther. 2018;17:306–11. https://doi. org/10.1177/1534735417713301	1

Part 3. Additional Details of Included SRs

Table 3. SRs characteristics of Interventions and Outcomes

**Citation**	**Year**	**Countries**	**Intervention Descriptions**	**Cognitive Measures**
			Cognitive Training/Rehabilitation	
Alwi, S. M. S et al. [38]	2021	USA (7), Denmark (1), South Korea (1), Germany (1)	**Type**: Cognitive training (4), compensatory strategies (5), cognitive and compensatory strategies (1)<br>**Length**: Ranged between 4 and 12 weeks<br>**Dose**: Ranged from daily sessions to 4 sessions per week.	**Objective:** Paced Auditory Serial Addition Test, Rey Auditory Verbal Learning Test, WAIS-IV (Digit span, symbol search), Letter FluSency Test, Delis-Kaplan Executive Function System, Brief Visuospatial Memory Test, CNS Vital Signs Computarized Test, Trial Making Test, Wechsler Test of Adult Reading, California Verbal Learning Test, Wisconsin Card Sorting Test, Hopkins Verbal Learning Test-Revised, Seoul Verbal Learning Test, Controlled Oral Word Association, Rivermead Behavioral Paragraph Recall Test, Useful Field of View, TAP (Alertness, reaction change, divided attention, go/no go, working memory and sustained attention, Wescheler Memory Scale-Revised, Learning and Memory-Test, Symbol Digit Modalities Test, 30-item Everyday Problems Test-Revised, Judgment of Line Orientation Test, Stroop Color, Word Inference Test.
Bergo, E. et al. [40]	2016	Not provided.	**Types**: Neuropsychological training online (1), virtual reality (1), computer exercises guided by a neuropsychologist (1), holistic mnemonic training (1), cognitive retraining and compensation techniques (1), cognitive rehabilitation and problem-solving therapy (1), individualized rehabilitation therapy (1)<br>**Length**: Ten weeks (2), 4 weeks (2), 6 weeks (1), 2 weeks (1), not reported (1).<br>**Dose**: Frequency: Three times a week (1), four times a week (1), once a week (2), not reported (3); Duration: Not reported (2), 30 minutes (1), 60 minutes (1), 90 minutes (1), 120 minutes (1), five hours (1)	**Subjective:** Cognitive Functioning Scale from the MOS burden (study-specific measure), Cognitive Failure Questionnaire, Short-Form 36 from the MOS.<br><br>**Objective:** Minimental State Examination, Trail making test A+B, Frontal Assessment Battery, Raven Progressive Matrices 47, Rey-Osterrieth Complex figure copy and recall, computerized neuropsychological tests, Modified Barthel Index, Neuropsychological Exam for Aphasia, Rey Auditory-Verbal Learning Test, Hopkins Verbal Learning Test, Controlled Oral Word Association Test, Dutch Adult Reading Test, Drie-Minuten-Toets (Three-Minute Test), Stroop Color-Word Test, Letter Digit Substitution Test, Memory Scanning Test, Visual Verbal Learning Test, direct and delayed recall, Concept Shifting Test, Category Fluency animals from the GIT, Digit Span, Test of Everyday Attention, Letter Fluency, CT professions from the GIT, Behavioural Assessment of the Dysexecutive Syndrome, Multidimensional Fatigue Inventory, Community Integration Questionnaire, Functional Assessment of Cancer Therapy brain version, the Mayo-Portland Adaptability Inventory-4, Repeatable Battery for the Assessment of Neuropsychological Status, Linear Analogue Self-Assessment scale of QOL, Caregiver QOL Index-Cancer, Profile of Mood States, Brief Fatigue Inventory.
Caponenetto, P. et al. [45]	2024	Not provided.	**Type**: Virtual reality (2), computerized cognitive training (3)<br>**Length**: The trials ranged from 3 to 52 weeks, with the majority using interventions of 12 weeks duration.<br>**Dose**: Frequency; 2 days per week (10), 3 days per week (6) or 5 days per week (5). Duration; aerobic exercise components being 20 (5) or 30 (6) minutes and mind-body exercise more commonly ranging from 45 to 60 (4) to 70 to 75 minutes (2).	**Subjective:** Cognitive Failures Questionnaire.<br><br>**Objective:** Auditory Continuous Performance Test, Box and Block Test, Backward Digit Span Test, Backward Visual Span Test, Forward Digit Span Test, Fugl-Meyer Scale, Forward Visual Span Test, Hopkins Verbal Learning Test-Revised, Korean-Modified Barthel Index, Korean version of the Mini-Mental Status Examination, Manual Function Test, Sustained Attention to Response Task, Stroop Colour and Word Test, Test of Everyday

				Attention, Trail Making Test, Visual Continuous Performance Test, Verbal Learning Test, Visual Learning Test, Wechsler Adult Intelligence Scale, Word of Colour Word in Word Color Test, Wechsler Memory Scale.
Castellino, S. M. et al. [46]	2014	Not provided.	**Type**: Cognitive remediation program (3), computerized cognitive program (3), group skills therapy (1), social skills (2), psychological intervention (1)<br>**Length**: 4-5 mo(1), 3 mo (2), 3-6 mo (1), not provided (6)<br>**Dose**: Duration: 50 min/week (1), 60 min/session (1), 2 hours/session (2); Frequency: 3 sessions (1), 15 sessions (1), 25 sessions (1), not provided (3).	**Subjective:** Child Development Inventory<br><br>**Objective:** Conners Continuous Performance Test, California Verbal Learning Test, California Verbal Learning Test Children's Version, Dellis-Kaplan Executive Function Test, Full-scale Intelligence Quotient, Intelligence Quotient, Wide Range Achievement Test, Wide Range Assessment of Memory and Learning 2nd Edition, Working Memory Index
Fernandes, H.A. et al. [54]	2019	Not provided.	**Type**: ST attention and memory (3), ST cognitive rehabilitation (43), ST cognitive behavioral therapy (1), CT cognitive rehabilitation (4), ST + CT for memory and executive function (1), ST memory + CT processing speed (1), ST + CT memory and attention (1).<br>**Length**: 4 weeks (7), 5 weeks (2), 6-8 weeks (7), 4 months (2), not provided (1)<br>**Dose**: Duration: One session (1), 10 session (1), 40 sessions (1), 60 sessions (1), Once a week (6), twice a week (3), 4 days/week (2), 5 days/week (3), once a month (1); Frequency:<br>20-30 min/session (1), 30-60 min/session (10), 75 min/session (1), 90 min/session (2), 120 min/session (4), not reported (1)	**Subjective:** Functional Assessment of Cancer Therapy-Cognitive, the Rivermead Everyday Behavioural Memory Test, Everyday Problems Test–Revised, Judgment of Line Total Correct, Cognitive Failures Questionnaire, Frontal Systems Behavior Scale, Memory Self-Efficacy Questionnaire, Multifactorial Memory Questionnaire, Metamemory in Adulthood-Anxiety scale, Multiple Ability Self-Report Questionnaire, Brief Assessment of Prospective Memory, European Organization for Research and Treatment of Cancer Core Quality of Life Questionnaire.<br><br>**Objective:** CNS Vital Signs Verbal Memory, CNS Vital Signs Visual Memory, Repeatable Battery for the Assessment of Neuropsychological Status, Mini Mental State Examination, Direct Assessment of Functional Status, Field of View Divided Attention and Selective Attention, CNS Vital Signs Continuous Performance Test, CNS Vital Signs Shifting Attention Test, CNS Vital Signs Stroop Test, CNS Vital Signs Symbol Digit Coding Test, CNS Vital Signs Finger Tapping Test, Rey Auditory Verbal Learning Test, Wechsler Adult Intelligence Scale-III Digit Span, Stroop Interference, Wechsler Adult Intelligence Scale-III Digit Symbol Coding, California Verbal Learning Test—Second Edition, Delis–Kaplan Executive Function System Trail Making Test, Wisconsin Card Sorting Test, Brief Assessment of Prospective Memory, Korean Controlled Oral Word Association Test, Cognitive Estimation Task, Letter Fluency, Trail Making Test, Hopkins Verbal Learning Test—Revised, Brief Visuospatial Memory Test-Revised, Cogstate Paired Associates, Cogstate One Card Learning, Cogstate Identification Speed, Cogstate One Back and Two Back Task Speed, Cogstate Groton Maze Learning, Cogstate Detection Task Speed, Wechsler Adult Intelligence Scale-IV Digit Span, Paced Auditory Serial Addition Test, D-KEFS 20 Questions Test, Wechsler Adult Intelligence Scale-IV Symbol Search, CNS Vital Signs Stroop Complex Reaction Time, Controlled Oral Word Association Test, The Symbol Digit Modalities Test, Wechsler Memory Scale-Revised Digit Span, Test Battery for Attentional Performance Working Memory and Sustained Attention, Test Battery for Attentional Performance Reaction Change, WebNeuro Verbal Memory, WebNeuro Attention/Concentration, WebNeuro Executive Functioning, WebNeuro Impulsivity, WebNeuro Working Memory Capacity, WebNeuro Information Processing Efficiency, and WebNeuro Response Speed.
He, F. et al. [57]	2022	Not provided.	**Type**: Cognitive training (6), cognitive rehabilitation (1), neurofeedback (1), mathematics intervention (1)<br>**Length**: 5-9 weeks (3), 4-5 months (2), 6 months (3), not provided (1)<br>**Dose**: Duration: Once a week (3), twice a week (1), 3 times/week (1), two hour weekly sessions (2), not provided (2); Frequency: 30-45 min/session (4), 1-2 hours/week (1), not provided (4),	**Subjective:** Child Behavior Checklist<br><br>**Objective:** Behavior Rating Inventory of Executive Function, Wechsler Abbreviated Scale of Intelligence (Digit Span, Letter-Number Sequencing, Spatial Span), Intelligence Quotient, Conners' Parent Rating Scale-3

Kim, Y. et al. [63]	2019	Not provided.	**Type**: Computarized cognitive training vs. waitlist (4), computarized cognitive training vs. unclear comparitor (2), computarized cognitive rehabilitation vs. unclear comparitor (1).<br>**Length**: 3-5 weeks (2), 6-8 weeks (3), 12 weeks (1), 15 weeks (1)<br>**Dose**: Duration: Once a week (1), 3-4 sessions/week (4), 5 days/week (1), 10 sessions/week (1); Frequency: 20-30 min/session (2), 30-60 min/session (5)	**Subjective:** Patient Reported Outcomes Measurement Information System-Cognition, Functional Assessment of Cancer Therapy-Cognition, Cognitive Failures Questionnaire, European Organization for Research and Treatment of Cancer Quality of Life Questionnaire, Squire Subjectie Memory Questionnaire, Behavioral Rating Inventory of Executive Function, Brief Assessment of Prospective Memory<br><br>**Objective:** Ability to reason and solve everyday problems, Verbal learning & remembering, Verbal fluency & word finding, Complex scanning & tracking, Cogstate, Paced Auditorial Serial Addition Test, Rey Auditory Verbal Learning Test, Digit span, Letter Fluency Test, Cognitive Estimation Task, Wisconsing Card Sorting Test, Delis-Kaplan Executive Function System, Symbol Search, Hopkins Verbal Learning Test-Revised, WebNeuro Cognitive Testing Battery, Test Battery for Attentional Performance, Rivermead Questionnaire of Experienced Attention Deficits, Wechsler Memory Scale-Revised, Useful Field of View
Langenbahn, D.M. et al. [66]	2013	Not provided.	**Type**: Cognitive rehabilitation (11); APT, strategy use, cognitive behavioural therapy, attention and memory training.<br>**Length**: 2 weeks (1), 3 weeks (1), 6 weeks (1), 4-5 months (2), 6 months (3), not provided (3)<br>**Dose**: Duration: Once a week (4), 4 sessions/week (1), 20 sessions (1), 50 sessions (1), not reported (4); Frequency: 60 min/week (1), 5 hours/session (1), not reported (9)	**Objective**: Boston Diagnostic Aphasia Evaluation, Grade Level in Arithmetic Skills, Continuous Performance Test, Wechsler Adult Intelligence Scale-Revised, Wechsler Memory Scale-Revised, Trail Making Test, Stroop, Wisconsin Card Sorting Test<br>*Measurement not reported for all studies, the majority describes cognitive domains assessed*
Pfeiffer, S.M. et al. [77]	2018	USA (10), Sweden (1)	**Type:** Cognitive rehabilitation program (4), maths (2), clinic based cognitive program (1), SMART/Amat-C program aimed at improving memory and attention (1), captain log computerised program aimed at improving executive function (1) cogmed RM computerised program aimed at period of improving attention and working memory (1), reading (1)<br>**Length:** 6 weeks (1), 2 mths (1), 3mths (1), 5 mths (2), 6 mths (4), 12 mths (2)<br>**Dose:**12-13.5 hours (3), 20-22.5 hours (2), 40-50 hours (4), 59.5 hours (1), 40-100 hours (1).	BBDT D Beery–Buktenica Developmental Test of Visual-Motor Integration; BRIEF D Behavior Rating Inventory of Executive Function; CBCL D Child Behavior Checklist; CPRS: LV-R D Conners' Parent Rating Sale: Long version revised; CTRS: LV-R D Conners' Teacher Rating Scale: Long Version – revised; Conners CPT D Conners' Continuous Performance Test: CVLT-C D The California Verbal Learning Test for Children; GDS D Gordon Diagnostic System; MVPT-3 D Motor Free Test of Visual Perception – 3rd version; NEPSY-II D NEPSY: A Developmental Neuropsychological Assessment: 2nd version; PIAT-R D Peabody Individual Achievement Test – revised; RAVLT D Rey Auditory Verbal Learning Test; ROCF D Rey-Osterrieth Com- plex Figure Test; SBIS-4 D Stanford-Binet Intelligence Scale – 4th Version; SSRS D Social Skills Rating System; WASI D Wechsler Abbreviated Scale of Intelligence; WISC-III D Wechsler Intelligence Scale for Children: 3rd version; WISC-IV D Wechsler Intelligence Scale for Children: 4th version; WJ-R – Woodcock–Johnson Tests of Achievement – revised; WRAML-2 D Wide Range Assessment of Memory and Learning: 2nd version; WRAT-R D Wide Range Achievement Test – Revised; WRAT-3 D Wide Range Achievement Test, 3rd Version
Sciancalepore, F. et al. [79]	2022	Not provided.	**Type:** Computer-based cognitive training (9)<br>**Length:** 5-9 weeks (6), 8-12 weeks (3)<br>**Dose:** N/A	WISC: Weschler Intelligence Scale for Children; CPT: Conners Continuous Performance Test; NTCB: National Institute of Health Toolbox Cognition Battery; CPRS: Conners' Parent Rating Scale; WRAML2: Wide Range Assessment of Memory and Learning 2; WMI: Working Memory Index.
Von Ah, D. et al. [87]	2020	Not provided.	**Type:** Cognitive rehabilitation (11), cognitive training (14), combined cognitive training and compensatory training (1), combined cognitive training and cognitive rehabilitation (1)<br>**Length:** Not provided (6), 2 wks (1), 4 wks (2), 5-6 wks (5), 8 wks (2), 3-4 months (5), 7-8 sessions (2), 48 sessions (1), 4-6 sessions (3), 5-16 sessions<br>**Dose:** Duration: Not provided (3), 1.25 hrs (1), 20-30 mins (3), 30-50 mins (6), 60 mins (7), 2 hrs (6), 45-90 mins (1); Frequency: Not provided (8), once to twice per wk (7), 4-5 times/wk (6), 6 times/wk (1), bi-weekly (1), monthly (3)	Not Provided.

Vuori, O. et al. [89]	2023	Netherlands (1), Australia (1).	**Type:** Psychoeducation and strategy training (2)<br>**Length:** 4-10 weeks (2)<br>**Dose:** 6 sessions (1), not provided (1)	**Objective:** cognitive performance (CNS VS, WAIS-III digit span, verbal fluency), cognitive functioning (CFQ), executive functions (BRIEF-A) (1)<br><br>**Subjective**: FACT-cog-3, BAPM, WebNeuro
Wolfe, K.R. et al. [91]	2012	Not provided.	**Type:** Cognitive training (2), computerised cognitive training (1)<br>**Length:** 12 wks (1), 4-5 mths (1), not provided (1)<br>**Dose:** 15-20 sessions (2), not provided (1)	Not Provided.
Yan, X. et al. [92]	2023	USA (5), China (1), France (1), Belgium (1), Denmark (1)	**Type:** Cognitive training (9)<br>**Length:** Range: 5 to 12 weeks<br>**Dose:** Duration: 20-60 min per session; Frequency: range: 3 to 5 times per week	Behavioral assessment of dysexecutive syndrome, Digit Symbol Coding Test, Digit Span Test-backwards, Digit Span Test- forward, Paced Auditory Serial Addition Task, Rey Auditory Verbal Learning Test, Trail Making Test-B, Trail Making Test-A, Useful Field of View, Wisconsin card sorting coding test, Cognitive Failure Questionnaire, Functional Assessment of Cancer Therapy-Cognitive Function, Patients Assessment of Own Functioning, Patient-Reported Outcomes Measurement Information System, Squire Subjective Memory Questionnaire
Zeng, Y. et al. [94]	2016	Not provided.	**Type:** EEG Neurofeedback (1), Cognitive rehabilitation (3) Psychoeducation (1), Cognitive training (2), Online home-based cognitive training (1), Memory intervention (2)<br>**Length:** 4-weeks (2), 5-weeks (1), 7-weeks (1), 8-weeks (2), 10-weeks (1), 12-weeks (1), 6-months (1), 2-years (1).<br>**Dose:** Not provided.	Not Provided.
Physical Activity/Exercise				
Akbari, P.S. et al. [37]	2023	Germany (6), Sweden (4), Denmark (2), Canada (2), USA (2), Japan (2), China (1), Iran (1) France (1), United Kingdom (1)	**Type:** Aerobic and resistance (13), aerobic (2), resistance (2), tracked physical activity (2), yoga (3)<br>**Length:** Ranged 3 weeks - 26 weeks<br>**Dose:** Not provided.	**Subjective:** EORTC-QLQ-30, perceived cognition questionnaire, cognitive failures questionnaire, PROMIS (unclear if it is PROMIS-Cog), Piper fatigue scale<br><br>**Objective:** d2 test of attention, memo test, Wilde intelligence test, test of attentional performance, MOCA, CogState, WAIS-III (Stroop and digit span), sustained attention response to task, trail making test
Baydoun, M. et al. [39]	2020	USA (3), Canada (2), Belgium (1), Belgium (1), Germany (1), Iran (1), India (1)	**Type:** Hatha yoga (4), individualized yoga sessions (1), Iyengar yoga (2), hatha and restorative yoga (1), type of yoga not specified (2)<br>**Length:** 4 weeks (2), 12 weeks (3), single session (1), 6 weeks (2), 8 weeks (1), 7 weeks (1)<br>**Dose:** Duration: Twice weekly (4), three sessions weekly (1), once weekly (2), single session (1), not provided (2); Frequency: 90 min (2), 80 min (1), not reported (1), 70 min (3), 60 min (3)	**Subjective:** Unclear name (Breast Cancer Prevention Trial? measures of cognitive complaints), Cognitive Failures Questionnaire, MD Anderson Symptom Inventory, EORTC-QLQ-30, Symptom of Stress Inventory.<br><br>**Objective:** Association/Dissociation Scale, Computarized Cognitive Testing Battery (tests not provided)
Bernal, J.D.K. et al. [41]	2023	Russia (1), Switzerland (2), Turkey (1), USA (3), Germany (3), Canada (1), Sweden (1), Netherlands (1),	**Type:** Coordinative exercise (1), mixed exercise (4), mixed exercise (8), mixed exercise (4), aerobic exercise (1), strenghtening exercise (1), aerobic exercise (3).<br>**Length:** Median intervention period was 12 (IQR 10–24) weeks.<br>**Dose:** Median session duration 45 mins (IQR 40–60); Physical activity intensity was reported in 18 (82%) studies. The median frequency of physical activity was 3 (IQR 2·5–5·0) days per week.	**Subjective:** EORTC-30-QOL, Pediatric Quality of Life Inventory, Checklist Individual Strength.<br><br>**Objective:** Auditory Consonant Trigrams, Beery-Buktenica Developmental Test of Visual-Motor Integration, Bruininks-Oseretsky Test of Motor Proficiency second edition, Bruininks-Oseretsky Test of Motor Proficiency-Short Form, Cambridge Neuropsychological Test Automated Battery, Checklist Individual Strength, Controlled Oral Word Association Test, Conners' Continuous Performance Test II, Delis-Kaplan Executive Function System,

		Spain (1), Saudi Arabia (1), Australia (1), China (2), Iran (1), Israel (1), Turkey (1), Taiwan (1)		Developmental Coordination Disorder Questionnaire 2007, European Organisation for Research and Treatment of Cancer Quality of Life Questionnaire Core 30, Kaufman Assessment Battery for Children second edition, Test for motor performance in oncology, Pediatric Quality of Life Inventory, Rey Auditory Verbal Learning Test, Rey Complex Figure Test, Test of Nonverbal Intelligence fourth edition, Wechsler Abbreviated Scale of Intelligence, Wechsler Intelligence Scale for Children fourth edition, Working Memory Test Battery for Children, and Wechsler Nonverbal Scale of Ability
Brunet, J. et al. [43]	2023	United States (24), Germany (11), Canada (10), Australia (6), Spain (5), Netherlands (3), Sweden (3), Switzerland (2), United Kingdom (2), Italy (2), Iran (1), Aus/New Zealand (1), South Korea (1), India (1), Finland (1), Japan (1), not reported (1)	**Type:** Aerobic endurance training (26), resistance/strength training (3), combined aerobic/endurance and resistance/strength training (24), yoga (7), Qigong/Tai Chi (5), exergaming (2), aquatic-based (1), unspecified PA (3)<br>**Length:** Ranged from a single session to 18 months<br>**Dose:** Frequency: 1-15 total sessions per week (when combining in-person and home-based sessions), Not reported (6); Duration: sessions ranged from five to 120 min (including warm-up and cool-down, where applicable), not specified (14).	**Subjective:** The Functional Assessment of Cancer Therapy—Cognitive Function, European Organization for Research and Treatment of Cancer Quality of Life Questionnaire–Core 30, Behavior Rating Inventory of Executive Function, Piper Fatigue Scale, Profile of Mood States, Symptoms of Stress Inventory, Breast Cancer Prevention Trial Symptom Checklist, Perceived Cognition Questionnaire, Medical Outcomes Study, Cognitive Failure Questionnaire, Patient-Reported Outcome Measurement Information System Applied Cognition, Wechsler Abbreviated Scale of Intelligence, MD Anderson Symptom Inventory, Social Problem-Solving Inventory, Perceived Cognitive Impairment, Multidimensional Fatigue Inventory, Multiple Abilities Self-Report Questionnaire, Mini-Mental State Exam, and Pediatric Quality of Life Inventory Multidimensional Fatigue Scale.<br><br>**Objective:** NIH Toolbox, Block Recall Test, D- KEFS (Color-Word Interference subtest), KABC-II NU (Test of Nonverbal Intelligence, Number Recall, Word Order, Rover, Atlantis & Atlantis recall subtests), WISC-IV (Cancellation, Coding, & Symbol Search subtests), HVLT, TMT, COWA, Animal Naming Test, Stroop test, Go/No Go, CPT, WISC-IV (DS, LNS, Coding, & Symbols subtests), WISC-III (Mazes subtest) Verbal & Figurative Fluency Tests, Stroop Test, TMT, CogState Battery, ACT, TMT, Stroop Test, Letter Digit Substitution Test, WAIS (DS subtest), Test of Everyday Attention, Visual Verbal Learning Test, WMS-III (Verbal Paired Associates subtest), Concept Shifting Test, GIT Letter and Category Fluency, Stroop Test WAIS-IV (DS, BD subtests), SART, D-KEFS, WAIS-III (DS & LNS subtests), FAB, RAVLT, TMT, F-A-S Test of Verbal Fluency, BCOG, WAIS- IV (LNS, Coding, & BD subtests), TMT, WMS- IV (LM-I & LM-II subtests), COWAT, RAVLT, TMT, Stroop Test, COWAT, WAIS-III (DS & Digit Symbol subtests), CANTAB, WISC-IV (DS, Similarities, BD, & Coding subtests), CPT, Map Mission Test, Visual Scanning, D-KEFS, ACT, RAVLT, WNV, Rey Complex Figure Test, COWAT, TMT, Stroop Test, Letter Comparison Task Spatial Memory Task, Flanker Task, Spatial WM Test, Task Switching Test, Letter Comparison Test, D2 Test of Attention,
Campbell, K.L. et al. [44]	2020	Not provided.	**Type:** Aerobic and resistance training (12), aerobic only (7), resistance only (3), mind-body exercise (7).<br>**Length:** Tirals ranged from 3 to 52 weeks, with the majority using interventions of 12 weeks duration.<br>**Dose:** Frequency: 2 to 7 days per week, with the majority of prescribed sessions being 2 days per week (10), 3 days per week (6) or 5 days per week (5); Duration: 15 to 90 minutes per session, with the majority of aerobic exercise components being 20 (5) or 30 (6) minutes and mind-body exercise more commonly ranging from 45 to 60 (4) to 70 to 75 (n = 2) minutes.	**Subjective:** European Organization for Research and Treatment of Cancer Quality of Life Questionnaire–Core 30, Fatigue Assessment Questionnaire, MD Anderson Symptom Inventory, Multidimensional Fatigue Inventory, Modified Fatigue Impact Scale, Piper Fatigue Scale, Profile of Mood States, Patient-Reported Outcome Measurement Information System Applied Cognition and General Cognitive Concerns<br><br>**Objective:** Controlled Oral Word Association, Functional Assessment of Cancer Therapy-Cognitive Function, Hopkins Verbal Learning Test, Trail Making Test A/B, Wechsler Adult Intelligence Scale-Third Edition (Digit Forwards/Backwards, Attention to Response, Block Design, Stroop, Letter/Number Sequencing & Coding), Wechsler Memory Scale-Fourth Edition (Brief Cognitive Status, Logical Memory), D2-Test of Attention, Auditory Consonant Trigram, Rey auditory verbal learning.

Dun, L. et al. [52]	2020	Not provided.	**Type:** Moderate intensity exercise (7), high intensity exercise (3).<br>**Length:** 2-4 weeks (4), 4-6 months (6)<br>*All interventions reported since it is unclear what studies measured cognition.*	Not Provided.
Fukushima, T. et al. [56]	2021	Germany (5), USA (2), Denmark (1), Brazil (1), South Korea (1), Australia (1), Finland (1), Ireland (1), Sweden (1), Latvia (1)	**Type:** Resistance exercise (5), aerobic exercise (3), resistance and aerobic exercise (7).<br>**Length:** 3 weeks (1), 6 weeks (3), 8 weeks (2), 12 weeks (5), 16 weeks (2), 9 months (1), 12 months (1).<br>**Dose:** Frequency: Daily (1), once a week (1), twice a week (4), three times/week (3), five times/week (2), 6 times/week (1), not provided (3); Duration: 150 min/week (1), 20-60 min/session (7), 70 min/session (1), not provided (6).	**Subjective:** European Organization for Research and Treatment of Cancer Quality of Life Questionnaire, Piper Fatigue Scale<br><br>**Objective:** D2 Test
Hiensch, A. E. et al. [59]	2023	Netherlands (7), Australia (3), Germany (4), USA (1), Norway (1)	**Type:** Aerobic exercise (n= 186), resistance exercise (n= 112), aerobic + resitance exercise (n= 740), resitance exercise + impact training (n= 77)<br>**Length:** Less or equal to 12 weeks (n= 471), 12-24 weeks (n= 348), more than 12 weeks (n= 256), not provided (n= 40)<br>**Dose:** Frequency: Two times/week (n= 918), five or more times/week (n=197); 30 or less min/week (n= 243), 30-60 min/week (n= 786), more than 60 min/week (n= 86); Duration: Moderate intensity (n= 311), moderate/vigorous to vigorous (n= 732), not provided (n= 72)	**Subjective:** European Organization for Research and Treatment Quality of Life Questionnaire 30, Multidimensional Fatigue Inventory, Checklist Individual Strength, Fatigue Assessment Questionnaire
Jesus, O.J. et al. [61]	2023	Not provided.	**Type:** Moderate/ high intensity exercise (6)<br>**Length:** 8 weeks (1), 12 weeks (2), 24 weeks (1), not provided (2)<br>**Dose:** 3 sessions/week (2), not provided (4); 60 min/session (1), 90 min/session (1), 150 min/week (2), not provided (2)	**Subjective:** NIH Toolbox, Patient Reported Outcomes Measurement Information System, Functional Assessment of Cancer Therapy-Cognition, European Organization for the Research and Treatment of Cancer Quality of Life Questionnaire<br><br>**Objective:** Hopkins Verbal Learning Test-Revised, Trial Making Tests, Stroop Test, CogState Battery (Delayed Recall, Groton Maze Learning Task, One-Back Test, International Shopping List), Letter Comparison, Spatial Working Memory, Flanker Task, Spatial Working Memory, Task Switching, Auditory Consonant Trigrams
Mikkelsen, M.K. et al. [69]	2020	France (1), Japan (1)	**Type:** Individualized phoned PA advice (on resistance and aerobic exercises) and booklet with exercises (1) Speed-feedback therapy on bicycle ergometers (1).<br>**Length:** 12 months (1) and 4 weeks (1)<br>**Dose:** Telephone advice; 2x a month for 6 months, and then 1x a month, Exercise: starting at 2x a week, hereafter adjusted to each participant; Duration; 12 months (1), 1X a week for 4 weeks; Time; 5 min. (1)	**Objective:** IQR, Frontal Assessment Battery
Myers, J.S. et al. [71]	2018	Not provided.	**Type:** Aerobic exercise (3), Resistance training (3), Combination of both aerobic exercise and resistance training (6), Aerobic exercise and psychoeducation (1), Mindfulness-based exercise - Yoga (5), tai chi (1), qigong (1), not reported (1)<br>**Length:** Not reported (6), 4–6 months (1), 12 weeks (7), 4 weeks (2), 7 weeks (1), 6 weeks (2), 10 weeks (2).<br>**Dose:** 3x/ week (1), 60 minutes, 2x/week (3), 60 mins (3), 2x/week (1), 5x/week (1), 60 mins PA, 30mins aqua aerobics and 2hrs psychoeducation for 7 sessions (1), 5 mins 1x/week (1), 75 mins (1), 3x/week yoga and 15 mins counselling 3-4x/week (1), 70 min 2x/week then 15 min 3x/week (1), 90 min 2x/week (1), 60 mins 2x/week (1), 90 mins 1-2x/week (1), not reported (3), multiple length options (1).	Memory loss and difficulty concentrating: 12-item URCC Symptom Inventory (1)<br>Forgetfulness and concentration: BCPT Cognitive Problems (2)<br>CNS Vital Signs software: psychomotor speed, reaction time,<br>FACT-COG, MEMO-test-memory WIT-working memory d2-focused attention and concentration, Fatigue Assessment Questionnaire-cognitive fatigue, TMT, Revised Piper Fatigue Scale cognitive and mood, fatigue subscale, EORTC-QLQ-C30-cognitive function, MFIS-cognitive fatigue, Neurotrax Comprehensive Testing Suite, Frontal Assessment Battery, POMS confusion, bewilderment dimension, SOSI cognitive disorganization subscale, PCQ-self report, CogState, Japanese version of CFQ, CFS cognitive fatigue subscale , MDASI-memory item , RAVLT, COWA, WAIS-III Digit Span and Digit Symbol, MASQ-verbal and visual subscale

Persoon, S. et al. [76]	2013	USA (1), Germany (2), Denmark (1), Switzerland (1)	**Type:** Aerobic + strength (2), aerobic + ADLs (1), strength training (1), aerobic training (1)<br>**Length:** 6-weeks (1), 12-weeks (1), not provided (3)<br>**Dose:** Frequency: 2-5 times/week (5); Duration: 10-40 mins/session (5)	**Subjective:** EORTC-QLQ-30 Cognitive domain
Ren, X. et al. [78]	2022	United States (2), England (2), Sweden (1), Germany (2), Australia (1), Ireland (1), Spain (1), Iran (1), India (1), India (1)	**Type:** Aerobic (6), Yoga (2), Qigong (1), aerobic + resistance (2), resistance (1)<br>**Length:** 6-weeks (1), 8-weeks (3), 10-weeks (1), 12-weeks (5),16-weeks (1), 24-weeks (1)<br>**Dose:** Frequency: 2 times/wk (3), 4 times/wk (1), 3 times/wk (5), 3-5 times/wk (1), 7 times/wk (12); Duration: 20-90 min per session	CFQ, Cognitive Failures Questionnaire; EORTC-QLQ, The European Organisation for Research and Treatment of Cancer quality of life<br>questionnaire; FACT-Cog, Functional Assessment of Cancer Therapy-Cognitive Function; FAQ, Fatigue Assessment Questionnaire; PFS, Piper fatigue scale; PROMIS, Patient-Reported Outcomes Measurement Information System scales; TMT, Trail Making A/B test.
Sharma, B. et al. [80]	2021	Not provided.	**Type:** Aerobic and muscle building exercises and games (1), Nintendo Wii Active Video Gaming aerobic and balance exercises (1)<br>**Length:** 10-12 weeks (2)<br>**Dose:** Frequency: 3-5 times/week (2); Duration: 30-90min/session (2)	Computerized cognitive testing suite Cambridge Neuropsychological Test Automated Battery [CANTAB], Conner Continuous Performance Test (a measure of sustained attention) and the Map-Mission test
Yang, H.Y. et al. [93]	2023	USA (3), United Kingdom (1), Sweeden (1)	**Type:** Walking (5)<br>**Length:** 10-weeks (1), 12-weeks (2), Two sessions (2)<br>**Dose:** 10000 steps per day (1), Individualised walking goals aiming for 10000 steps per day (1), Aiming for 30 minutes per day 5 days per week (1), 1 session of 30 minutes (1), 1 session of 10-30 mintes of walking (1)	**Objective:**<br>- Stroop task<br>- Sustained Attention to Response Task<br>- Flanker task<br>- Block Design from Wechsler Adult Intelligence Scale-III<br>- Forward and Backward Digit Span from Wechsler Adult Intelligence Scale-III<br>- 2-, 3-, and 4-item tasks<br>- 1–, 2–,and 3–black dot tasks<br>- Letter comparison task<br>- Switching paradigm task<br><br>**Subjective:**<br>- Cognitive Failures Questionnaire<br>- European Organization for Research and Treatment of Cancer–Core Quality of Life Questionnaire cognitive function subscale
Mind-body and Psychological/Behavioural				
Cifu, G. et al. [50]	2018	United States, Germany and Iran (N not reported)	**Type:** Mindfulness based stress reduction (3), mindfulness based stress reduction + other (yoga, metacognition treatment, self-care + Mediterranean diet (3).<br>**Length:** 6-8 weeks (2), 11 weeks (1), not provided (3)<br>**Dose: Frequency:** 2 sessions/week (1), not provided (4); Duration: 75 mins/session (1), not provided (4)	**Subjective:** Everyday Cognition, Cognitive and Affective Mindfulness Scale-Revised, European Organization for Research and Treatment of Cancer Quality of Life Questionnaire, Calgary Symptoms of Stress Inventory<br><br>**Objective:** Attentional Function Index, Stroop Color and Word Test
Myers, J.S. et al. [72]	2015	Not provided.	**Type:** Meditation (3), Mindfulness-based music therapy (1), Yoga (3), Tai Chi (1), Qigong (1), Haptotherapy (1), Neurofeedback (2), Acupuncture (1), Restorative environment (2).<br>**Length:** 6 weeks (2), 8 weeks (1), 4 weeks (1), 12 weeks (3), 10 weeks (3) Not reported (3), 15 weeks (1), 19 days (1)<br>**Dose:** 60 mins 2 /wk (2), 90 mins 7 /wk (1), 2.25 hours/week and one 6 hour session	Digit span, Digit symbol, COWA, RAVLT,<br>TMT -A, TMT-B, FACT-COG, POMS, SOSI, PCQ, Cogstate, BCPT cognitive problems scale, EORTC-QoL C30, MASQ IES-R, WAIS-III digit span and digit symbol, Qeeg ANT, Visual sequencing task, Wechsler digit span,

			in week 6 (1), 60 mins 1/wk (1), 2/wk (2), 60 min yoga sessions 7/wk (at least 3 per week in-person with instructor) (1), 90 mins 2/wk and 30 mins home practice 7/wk (1), 45 mins for 5 sessions (1), 33 mins, neurofeedback sessions (1), 33 mins 2/wk (1), 50 mins 4/wk education sessions and 50 mins 8 acupuncture sessions (1) , 20-30 min at least 3/wk (1), Exposure to a natural environment for 120 min/wk (1).	WISC-III/WAIS-III, Stop signal task, Tracking and pursuit task, SWAN, BRIEF parent and teacher version, AFI, Necker cube pattern control
Hines, S. et al. [60]	2014	USA(5), Germany (1)	**Type**: Cognitive behavioral therapy (5), neuropsychological training based on cognitive behavioral therapy (1).<br>**Type:** Meditation (2), Qigong (1), Yoga (1)<br>**Length:** 6-10 weeks (4)<br>**Dose:** Mainly 1 session per week	**Subjective:** Multiple Ability Self-Report Questionnaire<br><br>**Objective:** TAP Tonic Alertness test, RMBT Story Test, California Verbal Learning Test
Zhang, Y. et al. [96]	2017	Not provided.	**Type:** Meditation (2), Qigong (1), Yoga (1)<br>**Length:** 6-10 weeks (4)<br>**Dose:** Mainly 1 session per week	**Subjective:** FACT-Cog, EORTC-CF, and AFI.<br><br>**Objective:** RAVLT, Digit Symbol, and Stroop test.
Supportive Care				
Zhao, G. et al. [97]	2021	China (5)	**Type:** Health Education (5)	Not Provided.
Zhao, K. et al. [98]	2020	Not provided.	**Type:** Rehabilitation (5)	**Subjective:** Functional Independence Measure (FIM) - Cognitive
Wang, X. et al. [90]	2015	China (3)	**Type:** Comprehensive nursing care (3)	Not Provided.
Cheng, X. et al. [49]	2018	Korea (1), UK (1), Sweden (1), China (1), Australia (1), Netherlands (2)	**Type:** Education + behavioral training + stress management + cognitive therapy + emotional support (1), telephone supportive care (2), in-hospital care + follow up support + caregivers' training (1), telephone follow up + in person end-of-treatment session (1), not provided (2)<br>**Length:** 7 weeks (1), 6 months (2), 12 months (3), 18 months (1)	**Subjective:** European Organization for Research and Treatment of Cancer Quality of Life Questionnaire C30 version 3.0 core questionnaire
Mixed				
Binarelli, G. et al. [1]	2023	Not provided.	**Type:** Cognitive training (20), physical activity (11) , mind-body (11), and multi-modal (4).<br>**Length:** 6-8 weeks (3), 12 weeks (8), 6 weeks (6), 15 weeks (1), 8 weeks (9), 3 months (1), 6 months (3), 4 weeks (5), 5 weeks (1), not reported (5), 16 weeks (1), 12 months (1), 10 weeks (1)<br>**Dose:** 10 sessions (1), 48 sessions (1), 4 sessions/week (2), 5 days/week (4), 9 sessions (1), 3 sessions/week (5), 2 sessions/week (8), 1 session/week (12), 7 to 28 sessions (1), 12 sessions (1), not reported (4), 1 session (1), 8 sessions (1), daily (1), 4 sessions (1), 14 sessions (1); 60 min/session (15), 30 min/session (5), 40 min/session (1), 120 min/session (6), 45 min/sesion (4), 150 min/session (4), 90 min/session (5), 5 min/session (1), 12 min/session (1), 180 min/session (1), 75 min/session (1), not reported (1)	Not provided (Provides functions assessed, not the name of the instruments)
Binarelli, G. et al. [42]	2021	Not provided.	**Type:** Cognitive simulation (16), computerized physical activity (4)<br>**Length:** 6 months (1), 6 weeks (2), 5 - 9 weeks (2); 15 weeks (1), 3 months (1); 12 weeks (1); no reported (1); 4 weeks (4); 10 weeks (1); 8 weeks (2); 3 - 5 weeks (1);	**Objective:** Alloway Working Memory Assessment, Rey Auditory Verbal Learning Test, Brief Assessment of Prospective Memory, Category Fluency, Controlled Oral Word Association Test, Conners′ Continuous Performance Test, Concept Shifting Test, California Verbal Learning Test, Delis- Kaplan executive function

			24 weeks (1); 6 months (1)<br>**Dose:** 3-4 times/week (1); 3 times/week (2); unclear (7); 25 sessions (1); 30 sessions (1); 9 sessions (1); 6 times/week (1); 48 sessions (1); once a week (2); five times/week (1); 4 times/week (1); 60 min/session (5); 20-45 min/session (6); 45-60 min/session (2); 120 min/session (2); 30-60 min/session (1); 90 min/session (1); unclear (1); 5 min/session (1)	system, Drie-Minuten Test, Esame neuropsiclogico per l′afasia, Frontal assessment battery, Frontal Systems Behavior Scale, Hopkins Verbal Learning Test Revised, Letter Digit Substitution Test, Memory Scanning Test, Paced Auditory Serial Addition Test, Raven′s Coloured Progressive Matrices, Rey-Osterrieth Complex Figure, Stroop Color-World Test, Symbol Digit Modalities Test, Telephone-based Assessment of Neuropsychological Status, Useful Field of View, Wechsler Abbreviated Scale of Intelligence, 2nd Edition, Wisconsin Card Sorting Test, Wechsler Intelligence Scale for Children, Woodcock–Johnson Tests of Cognitive Abilities, Wide Range Assessment of Memory and Learning.<br><br>**Subjective:** Behavior Rating Inventory of Executive Function, Conner's Parent Rating Scale, Multifactorial Memory Questionnaire, Patient Assessment of Own Functioning Inventory, Beck Cognitive Insight Scale, Cognitive Failures Questionnaire, Cognitive Functioning Scale from the European Organisation for Research and Treatment of Cancer—Quality of Life Questionnaire, Functional Assessment of Cancer Therapy-Cognitive, Mental Fatigue, Perceived Cognitive Impairment, Perceived Cognitive Abilities<br>Patient-Reported Outcomes Measurement Information System (unclear if PROMIS Cog), Side Effects Rating Scale, Squire Subjective Memory Questionnaire.
Chan, R.J. et al. [47]	2015	USA (4), Canada (1), France (1), Japan (1)	**Type:** Cognitive behavioural therapy (4), mindfulness (1), physical activity (2)<br>**Length:** 4 weeks (1), 6 weeks (1), 7 weeks (1), 8 weeks (2), 10 weeks (1), 12 weeks (1)<br>**Dose:** 2 times/week (1), 4 times/week (1), 10 sessions (1), 2 hours/session (1), not provided (3)	**Subjective:** Behaviour Rating Inventory of Executive Function Global Executive, Multiple Abilities Self-Report Questionnaire, Functional Assessment of Cancer Therapy-Cognitive Function, European Organisation for Research and Treatment of Cancer-Cognitive functioning subscale, Symptoms of stress inventory (SOSI) cognition subscale, Profile of moods scale (POMS) concentration subscale<br><br>**Objective:** Wisconsin card sorting test Delis–kaplan (letter fluency), Digit span, Symbol search, Hopkins verbal learning test-revised,Trail Making Test-B Colour word trail, Colour word switching trail, Digit symbol-coding (subtest of wechsler adult intelligence scale), California verbal learning test, Rey auditory verbal learning test (AVLT), rey auditory verbal, Digit span learning test (AVLT), rivermead behavioural paragraph recall test, Frontal assessment battery
Cheng, A.S.K. et al. [48]	2022	Not provided.	**Type:** Virtual reality (1), cognitive training (9), cognitive rehabilitation (5), meditation (4), psychoeducation and cognitive exercises (1), cognitive behavioral therapy (3), yoga (1), psychoeducation (1), mixed (cognitive rehabilitation vs. aerobic exercise vs. cognitive training vs. cognitive rehabilitation + aerobic exercise; 1), psychological nursing intervention (1)<br>**Length:** 4 weeks (3), 5 weeks (1), 6-8 weeks (14), 12 weeks (5), 15 weeks (1), 6 months (3)<br>**Dose:** Duration: 60 sessions (1), 2 days/week (2), 5 days/week (2), not provided (22); Frequency: 40-60 min/session (3), 120 min/session (1), 120 min/week (1), 2.5 hours/session (1), not provided (21)	**Subjective:** Attentional Function Index, Cognitive Failure Questionnaire, Functional Assessment of Cancer Therapy-Cognitive Function, Multiple Ability Self-report Questionnaire, Memory Self-Efficacy Questionnaire, Patient's Assessment of Own Functioning InventoryI, Patient Reported Outcomes Measurement Information System-Applied Cognition Abilities and General Concerns<br><br>**Objective:** Cogstate battery, Mini Mental State Examination, Montral Cognitive Assessment, Useful Field of View Test, Brown Attention-Deficit Disorder Scale, igit Span, Digit Symbol, Auditory-Verbal Learning Test, Brief Visuospatial Memory Test-Revised, CNS Vital Signs Verbal Memory, California Verbal Learning Test, Hopkins Verbal Learning Test-Revised, Rey Auditory Verbal Learning Test; Repeatable Battery for Neuropsychological Status; the 2nd Edition of Rivermead Behavioral Memory Test, Web-Neuro Verbal Memory, Behavioral Rating Inventory of Executive Function, Digit Span Backward, Stroop Trial, Trial Making Tests A and B, Controlled Oral Word Association Test, Verbal Fluency Test
Drijver, A.J. et al. [51]	2022	Not provided.	**Type:** Self-care + self-hypnosis (1), yoga (1), acupuncture (1), cognitive behavioral therapy for insomnia (1), progressive music relaxation vs. autogenic training (1)<br>**Length:** 4 weeks (1), 8 weeks (1), not provided (3)<br>**Dose:** 2 sessions/week, 75 mins/session (1), not provided (4)	**Subjective:** Functional Assessment of Cancer Therapy-Cognitive Function, MD Anderson Symptom Inventory, Brown Attention-Deficit Disorder Scale, Attentional Function Index, European Organisation for Research and Treatment for Cancer Quality of Life Questionnaire.

				**Objective:** Buschke Selective Reminding Test.
Egset, K.S. et al. [53]	2024	Not provided.	**Type:** Cognitive rehabilitation (4), cognitive training (8), neurofeedback (1), physical activity (2), active video-gaming (1), problem-solving therapy (1)	**Subjective:** Behavior Rating Inventory of Executive Function, Child Behavior Check List, Conners' Continuous Performance Test, Conners' Parent Rating Scale, Conners' Teacher Rating Scale, Inattention scale, Strengths and Weaknesses of ADHD-Symptoms and Normal Behavior,<br><br>**Objective:** Auditory Consonant Trigrams, Automated Working Memory Assessment, Behavior Regulation Index, Brain Tumour, Cambridge Neuropsychological Test Automated Battery, California Verbal Learning Test, Central Nervous System, Color Word Interference Test, Controlled Oral Word Association Test, Developmental Neuropsychological Assessment 2nd version, Delis-Kaplan Executive Functioning System, Executive Functions, Rey Auditory Verbal Learning Test, Trail Making Test, Trail Making Test B, Wechsler Adult Intelligence Scale, Wechsler Intelligence Scale for Children, Wide Range Assessment of Memory and Learning, Wisconsin Card Sorting Test 4th edition, Woodcock-Johnson Tests of Achievement 3rd edition, and Working Memory Test Battery for Children.
Floyd, R. et al. [55]	2021	USA (6), China (1), Canada (1), UK (1), South Korea (1)	**Type:** Cognitive training (6), exercise (2), acupuncture (1), Tibetan sound meditation (1)<br>**Length:** 2 weeks (1), 8 weeks (1), 12 weeks (1), 24 weeks (1), not provided (6)<br>**Dose:** Duration: 30-60 min/session (4), not provided (6); Frequency: Once a week (1), four times/week (1), five times/week (1), not provided (7).	**Subjective:** Functional Assessment of Cancer-Therapy Cognitive Function, Squire Subjective Memory Questionnaire, Cognitive Failures Questionnaire, Behavioural Rating Inventory of Executive Function, Mood and Anxiety Symptom Questionnaire.<br><br>**Objective:** Auditory-Verbal Learning Tests (1-3), California Verbal Learning Test, California Verbal Learning Test-2, Clock-Drawing Test, Color-Word-Interference, Controlled Oral Word Association Test, Delis Kaplan Executive Function (Color word condition), Delis-Kaplan Executive Function System, Delis-Kaplan-Colour-Word Interference Test (Color-Word and Switching Trials), Digit span test (Forwards and backwards), Digit Symbol-Coding subtest, Digit symbol test, Hopkin's Verbal Learning Test-Revised, Korean - Controlled Oral Word Association Test, Letter-Number sequencing test, Rey Auditory Verbal Learning Test, Rivermead Behavioral Paragraph Recall Test, Seoul Verbal Learning Test, Stroop test, Sustained Attention to Response Task, Symbol digit modality test, Symbol Digit Modalities Test, Telephone-Based Assessment of Neuropsychological Status, Trail Making Number-Letter Trial, Trial Making Test (Part A and B), Useful Field of View, Verbal Fluency Test, Wechsler Adult Intelligence Scale-III, Wechsler Adult Intelligence Scale 4th edition (letter number sequencing test and symbol search and coding test), Wisconsin Card Sorting Test.
He, K. et al. [58]	2023	Israel (1), Iran (1), Latvia (1), Netherlands (1)	**Type:** In-person sessions + tele-rehabilitation (1), neurofeedback (1), physical activity (2)<br>**Length:** Less or equal to 12 weeks (n= 471), 12-24 weeks (n= 348), more than 12 weeks (n= 256), not provided (n= 40)<br>**Dose:** Two times/week (n= 918), five or more times/week (n=197), 30 or less min/week (n= 243), 30-60 min/week (n= 786), more than 60 min/week (n= 86); Moderate intensity (n= 311), moderate/vigorous to vigorous (n= 732), not provided (n= 72)	**Subjective:** European Organization for Research and Treatment Quality of Life Quesitonnaire 30, European Organization for Research and Treatment Quality of Life Quesitonnaire Breast Cancer,<br><br>**Objective:** Behavior Rating Inventory of Executive Function, Montreal Cognitive Assessment, Wechsler short memory scale
Kasteler, R. et al. [62]	2023	Not provided.	**Type:** Physical activity (3), online cognitive training (5), behavioral interventions (4)<br>**Length:** 2 weeks (1), 5 weeks (1), 8-16 weeks (8), 4-8 months (1), not provided (1)	**Objective:** Automated Working Memory Assessment, Cambridge Neuropsychological Test Automated Battery, Children's Auditory Verbal Learning Test-2, List Sorting Working Memory, Rey Auditory Verbal Learning Test, Wechsler Intelligence Scale for Children, Wide Range Assessment of Memory and Learning, mean reaction time

			**Dose:** Frequency: Twice a week (1), 3 sessions/week (3), 5 days/week (2), not provided (6); Duration: 30-45 min/session (3), 90 min/session (3), not provided (6)	on correct answers on Attention Network Tasks, NIH toolbox, Conners Continuous Performance Test, Map Mission (14), visual scanning from Dellis-Kaplan Executive Function Test, Behavior Rating Inventory of Executive Function.
Kirkman, M.A. et al. [64]	2022	Canada (1), Germany (1), not provided (1)	**Type:** Cognitive rehabilitation (1), diet (1), goal management training (1)<br>**Length:** 2 weeks (1), not provided (2)<br>**Dose:** 3 sessions/week (1), 8 sessions (1); 50 min/session (1), not provided (1); 3 days of ketogenic diet , followed by three days of fasting and then another three days of ketogenic diet. From day 10 onwards, no dietary restrictions were implemented	**Subjective:** European Organization for Research and Treatment of Cancer Quality of Life Questionnaire, Behavior Rating Inventory of Executive Function for Adults, General Self-Efficacy Scale, Frontal Systems Behavior Scale, Positive and Negative Affect Schedule<br><br>**Objective:** Repeatable Battery for the Assessment of Neuropsychological Status, Trial Making Tests (Parts A and B), Test of Everyday Attention, Sustained Attention to Response Task, Behavioral Assessment of the Dysexecutive Syndrome Zoo Map Test, Hotel Test, Hopkins Verbal Learning Test-Revised, Mini Mental State Examination, d2 test of attention
Kirkman, M.A. et al. [65]	2023	Spain (1), Netherlands (5), USA (3), Canada (1), Multi-Country (2), Germany (1), Korea (3), Italy (2), Austria (1), Poland (1), Brazil (1)	**Type:** Cognitive rehabilitation (11), cognitive training (6), physical activity (1), hyperbaric oxygen therapy (2), ketogenic diet and intermitent fasting (1)<br>**Length:** 2 weeks (1), 4-6 weeks (5), 6-8 weeks (1), 10 weeks (2), 12 weeks (2), 6 months (1), not provided (7)<br>**Dose:** Frequency: Once a week (4), 3-4 times/week (2), 5-6 days/week (5), 25 sessions (1), not provided (9); Duration: 30-60 min/session (9), 90 min/session (1), 120-125 min/session (2), not provided (9)	**Objective:** Mini Mental State Examination, Addenbrooke's Cognitive Examination III, Repeatable Battery for the Assessment of Neuropsychological Status<br>*Information reported for some but not all studies*
Liu, Y. et al. [67]	2023	USA (8), UK (1), China (3)	**Type:** Hatha yoga (1), walking intervention (1), Kirtan Kriya (1), cognitive training (1), Tibetan sound meditation (1), 6 healing sounds and gentle exercise (1), acupuncture therapy (1), Memory and Attention Adaptation Training (2), electroacupuncture (EA/TNS+BA- Electroacupuncture Trigeminal Nerve Stimulation and Body Acupuncture) (1), CALM, Managing Cancer and Living Meaningfully (1), QG/TCE, qigong and tai chi easy (1)	**Subjective:**<br>BCPT - Breast Cancer Preven-tion Trial Cognitive Problems Scale, FACT-Cog - Functional assessment of Cancer Therapy–Cognitive Function (5), MMSE - Mini-Mental State Examination;, MASQ - Multiple Ability Self-report Questionnaire, CFQ - Cognitive Failures Questionnaire; BRIEF - Behavioral Rating Inventory of Executive Function; FACT-Cog PCI - Functional assessment of Cancer Therapy–Cognitive Function Perceived Cognitive Impairment (2), MoCA - Montreal Cognitive assessment.<br><br>**Objective:**<br>memory (via retrospective memory and prospective memory), verbal memory (via CVLT-II -California Verbal Learning (2)), processing speed (via digit symbol test); processing<br>speed (via Trail Making Number-Letter Trial, Color Word Trial, DKEFS - The Delis-Kaplan Executive Function System; Digit Symbol Coding and WAIS-III), executive function (via a computerized version of the Stroop task), working memory (via digit span test (4)); attention (via SART); perceptual organization (via WAIS- III), Immediate and delayed verbal memory (via HVLT-R); verbal fluency (via COWA - controlled oral word association test); processing speed and executive attention (via Trail Making Test A and B); cognitive flexibility (via WCST - Wisconsin Card Sorting Test), verbal memory (via HVLT-R- Hopkins Verbal Learning Test–Revised), processing speed (via digit symbol test (2)); verbal fluency (via COWA); verbal memory (via RALVT - Rey Auditory Verbal Learning Test; RM, retrospective memory); memory (via RAVLT), processing speed, executive function (via Trail Making Test A and B); memory (via<br>AVLT - Auditory-Verbal Learning Test), attention, cognitive processing speed, and visual working memory (via

				SDMT - Symbol Digit Modality Test); visual screening (via CDT -, clock-drawing test); executive functions (via TMT-B - trail-making test part B); attentional function and working memory (via forward and reverse digit span)
Mackenzie, L. et al. [68]	2022	USA (21), Australia (5), Belgium (1), France (2), South Korea (1), The Netherlands (2), Canada (1), UK (1), Japan (1) , Iran (2), Germany (2), India (1), Sweden (1), China (2), Denmark (2)	**Type:** cognitive training (15), cognitive behavioural therapy (4), physical activity (15), Supportive therapies (11)<br>**Length & Dose:** Cognitive training interventions (1) each: 3 months, 3 x week for 60 min each session, 15-week, 7-week group, 30 training sessions over 6 weeks, 9 session over 3mths, 5-week (2 hrs per week), 48 sessions over 12 weeks, 4 weekly 2 hr sessions, 8 sessions, 10 hours within 6-8 weeks, 4 week web-based, 12 weeks face to face and 6 telephone health coaching sessions, Three sessions per week for 12 weeks, 10 x 1-hr sessions over 6–8 weeks, 1 h/day, 5 days/week for 8 weeks<br>CBT (1) each**:** 4 x biweekly individual sessions, 8 x weekly sessions, 5 – 26 individual sessions, 12 weeks x 2 hr sessions.<br>Physical activity intervention (1) each: 150 min/wk aerobic exercise with two 45 mins supervised sessions per week. x 2 30-min unsupervised home sessions, 3 weekly moderate intensity mixed aerobic and resistance exercise + 9 dietetic consultation s for 26 weeks, 30 mins x 5 times a week for 12 weeks, Per week for 12 weeks, 12 weekly sessions, 1 x week for 4 weeks, 8 w 3 x week for 12 weeks, weekly 60- min Sessions + 15 min at home twice a day, 10- week program of 2 x 90 mins per week.<br>8 weeks x 2 per week group setting and x 1 week home practice, Three sessions per week for 12 weeks, 6 weekly discussion groups, 12 individual supervised exercise sessions + home based exercise over 12 weeks, 2 x week over 12 weeks (3), 6 weeks daily sessions of 60 min three supervised.<br>Supportive therapy interventions (1) each: One week + 4 days of follow-up after 2 months, 3 to 6 sessions each lasting 30 minutes, 5 x week for 4-hrs or telemedicine delivery, 12 phone calls over 6- months, 8 x weekly 2-hr classes, 4 x week education + 8 x 50- minute acupuncture weekly, 2 x week for 6 weeks x 8 sessions, 6 weeks and 2 hrs per week, 6 days, Two 4-week courses of acupuncture with a 3-day rest. Every week, patients were treated once a day for 5 days.	**Subjective:**<br>Beck Cognitive Insight Scale (BCIS),<br>FACT-Cog<br>Cognitive Failures Questionnaire (CFQ)<br>Patients Assessment of Own Functioning (PAOFI)<br>FACT Cog Brief Assessment of Prospective Memory EORTC-QLQ-C30 (Cognitive function)<br>Metamemory in Adulthood Questionnaire (MIA). Memory Self-Efficacy Questionnaire (MSEQ)<br>FACT-Cog Brief Assessment of Prospective Memory<br>FACT-Cog, Squire Subjective Memory Questionnaire (SSMQ)<br>Patient Assessment of Own Functioning Inventory (PAOFI)<br>Multiple Ability Self-Report Questionnaire (MASQ)<br>Alertness Behaviour subscale<br>EORTC QLQ-C30 (Cognitive function)<br>EORTC QLQ C30 (Cognitive function)<br>Breast Cancer Prevention Trial (BCPT) Symptom Checklist<br>Cognitive Failures Questionnaire (CFQ)<br>Patient Reported Outcomes Measurement Information System (PROMIS)<br>EORTC QoL C30 (Cognitive function), FACT-Cog<br>EORTC QoL C30 (Cognitive function)<br>FACT-Cog Self-rated mental acuity, concentration, memory, verbal fluency.<br>Fatigue Assessment Questionnaire (cognitive fatigue)<br>Everyday Cognition (ECog) scale, FACT-Cog<br><br>**Objective:**<br>Cognitive Failure Questionnaire (CFQ) My CQ: Trail making tests, Simple, Choice, Go No Go reaction time, Visual and Verbal Memory Recognition, N-back Coding<br>Cogstate online test battery<br>Rey Auditory Verbal Learning Test (RAVLT), stroop test, digit symbol and span tests, Wechsler Adult Intelligence Scale (WAIS-III)<br>Paced Auditory Serial Addition Test (PASAT), RAVLT.<br>Grober and Buschke test for anterograde episodic memory, D2 test for attention and concentration , verbal fluency test, Trail Making tests, WAIS IV.<br>Brief Visual Memory Test Revised, RAVLT, PASAT, Trail Making Tests<br>Wisconsin card sorting test (WCST), Delis-Kaplan Executive Function System (D KEFS), Hopkins Verbal Learning Test Revised (HVLT-R), WAIS, Behavioral Rating Inventory of Executive Function (BRIEF)<br>Repeatable Battery for the Assessment of Neuropsycho logical Status (RBANS) Trail making test

				HVLT-R, Brief Visuospatial Memory Test— Revised, Rivermead Everyday Behavioural Memory Test<br>Useful field of view (UFOV®) test. NIH Toolbox Cognition Battery (NIHTB CB)<br>Online test battery WebNeuro: Digit and Spatial Span (Wechsler Memory Scale WMS), California Verbal Learning Test (CVLT), Trail Making, Stroop, Finger Tapping, and Rey Complex Figure Test.<br>Digit span, K-COWAT Korean Controlled Oral Word Association Test; K WAIS, SVLT Seoul Verbal Learning Test, Trail Making Tests<br>WMS (IV), General Cognitive Screener (BCOG), Trail Making Tests, WAIS IV, Logical Memory I and II (LMI, LMII), COWAT, Block Design (BD)<br>RAVLT, Rivermead Everyday Behavioural Memory Test, Useful Field of View (UFOV)<br>Online CNS Vital Signs test battery<br>CVLT-II, DKEFS<br>CVLT SDMT<br>SDMT, reaction time task<br>HVLT-R, Trail making Tests, COWAT, Stroop test (with fMRI)<br>Test of Attentional Performance<br>Stroop test, forwards/back digit span and WAIS Block Design, Sustained Attention to Response Task (SART).<br>WAIS-III (digit span and letter number sequencing)<br>NIH Cognitive Toolbox<br>Frontal Assessment Battery (FAB)<br>RAVLT 1-5 total, F-A-S test of verbal fluency, Trail making test<br>Cogstate online test battery<br>WMS (IV), General Cognitive Screener (BCOG), Trail Making Tests, WAIS-IV, Logical Memory I and II (LMI, LMII), COWAT, Block Design (BD)<br>Trail making tests (2)<br>Prospective and Retrospective Memory Questionnaire (PRMQ).<br>Online NeuroTrax test battery<br>Digit span test, SDMT, COWAT, RAVLT.<br>AVLT, Verbal fluency test, symbol digit modality test, clock drawing test, TMT-B.<br>Attentional Function Stroop test Index (AFI)
Morean, D.F. et al. [70]	2015	Not provided.	**Type:** Classic resistance (device-based) strength-training (1), Restorative experiences (1), Cognitive rehabilitation and computerised cognitive training program (2), MAAT (Memory and Attention Adaptation Training) (2), Neuropsychological Therapy (1), Techniques of stress management (1)<br>**Length:** 12 weeks (2), 4 weeks (1), 6 weeks (2), 3-5 weeks (1), 8 weeks (1), variable (1)<br>**Dose:** 60 mins, 2x per week; 20-30 mins, 3x per week; 2hrs in class and 20min at home, 1-4x per week, 30-50 mins, monthly with phone contact in between; 20-30 mins, 4x per week; 60 mins, 2x per week; 60 mins, 4x per week.	d2 Test of Attention, Stress Test, Memo-Test, Wild-Intelligenz-Test, TAS, WAIS, TMT-A, TMT-B,<br>CNS, Symbol Digit Test, HVLT-R,<br>Brief Visuospatial Memory Test,<br>Paced Auditory Serial Addition Test,<br>Benton Judgment of Line Orientation, CVLT-II, WMS-III, Logical Memory I,<br>WMS-III, Logical Memory II, WAIS-III, Digit Symbol Coding, Stroop Color-Word Test, D-KEFS
Oh, P.J. et al. [73]	2016	USA (8), Netherlands (2), Australia (1), Italy (1), Germany (2).	**Type:** Tibetan sound meditation (1), Qigong (1), Neuropsychological and computerised training (2), ACTIVE (Advanced Cognitive Training for Independent and Vital Elderly trial)(1), Cognitive rehabilitation (4), Executive function training program (1), CBT (1), Memory and attention adaptation training (1), Attention-	Digit span, Trail Making Test, R-BANS<br>Necker Cube Pattern Control Stroop Color and Word Test, Test of Everyday Attention Letter Digit Substitution Test,<br>Rey Auditory Verbal Learning Test,

			restoring/based training (2).<br>**Length:** 7 weeks (1), 3 months (1), Not reported (1), 8 weeks (1), 6 weeks (2), 6 months (1), 12 weeks (1), 4 weeks (3), 10 weeks (1), 3-5 weeks (1), 6-8 weeks (1).<br>**Dose**: Duration: 60 mins 1/wk (2), 20 to 30 mins 3/wk (1), 30 mins/session, total not reported (1), 30 to 50 mins /session (1), 120 mins 1/wk (1) , 60 mins; Frquency: 5–26 sessions plus two booster sessions/6 months (1), 100 mins 3wk (1), 60 mins 2/wk (1), 90 mins 1/wk (1), 60 mins 4/wk (2) 60 mins; 10 sessions for 6–8 weeks (1)	Visual Verbal Learning Test, Digit symbol,Memory Scanning Test, Corsi Test, Category fluency, BRIEF, BADS, HVLT-R, WAIS-III, R-BANS<br>MMSE
Oldacres, L. et al. [74]	2023	USA (12), Belgium (2), Canada (2), Denmark (1), China (3), UK (1), Australia (1), Malaysia (1)	**Type:** Psychoeducational/psychosocial (7), Cognitive behavioural therapy/compensatory strategies (6), Accupunture (3), Music listening and meditation (1), Brain-training (5), Exercise (4)<br>**Length:** 3 months (3), 15 weeks (1), 8 weeks (6), 3-6 months (1), 6 months (2), Not clearly included (4), 12 weeks (5), 2 years (1), 24 weeks (1), 4 weeks (1), 6-8 weeks (1)<br>**Dose:** Frequency: 4 sessions (1), 40 mins 4 x/wk (1), 30 mins 2 x/wk(1), Between 3 and 6 individualised sessions (1), 45-60 minutes 9 sessions (1), 9 sessions, 8 group sessions + a six-hour retreat day (1), Not clearly included (12); Duration: 30-45 mins 1x/wk (1), 12 mins of listening 7x/wk (1), 20-30 mins 1x/wk (1), 60 mins 1x/month (1), 5x /wk (1), 60 mins 10 sessions (1), 30 mins 5x/wk (1), 45-60 mins 1x /wk (1), 2 x/wk (1)	EORTC-QLQ-C30, FACT-Cog, CogState, MoCA, MMSE, PRMQ, Grober and Buschke test, D2 test, TMT-A&B, WAIS-IV, CNS Vital Signs Battery, CVLT2, D-KEFS, DSC, MASQ, TBANS, SDT, Stroop Test, WAIS-III, SART, WAIS, CFQ, COWA, HVLT, CPR, GWT, ACS, WCST, FACIT, RBMT, AVLT, VFT, SDMT, CDT, Rey Auditory Verbal Learning Test (RAVLT, UFOV,, SSMQ, DS-T
Park J.H. et al. [75]	2023	USA (2), Japan (1), Denmark (1)	**Type:** Cognitive rehabilitation (8): Web-based cognitive training (1), web-based brain training (1), cognitive rehab program (1), CBT Memory and Attention Adaptation Training (2), Computerized Executive Function training (1), Speed of processing-Double Decision program (1), Cognitive training focused on memory or processing speed (1).; CBT (6): psychotherapy (2), mindfulness-based stress reduction (1), imagery-based behavioral intervention (1), Tibetan Sound Meditation program (1), mindfulness- based stress reduction and mindfulness-based cognitive therapy (1); Exercise (7): walking (1), mod-vigorous (1), Qigong (2), gentle (1), mod or high interval (2)<br>Other: acupuncture (1)<br>**Length:** Less than 8 weels (7), 8–12 weeks (13), more than 12 weeks (3)	AVLT, Audi·tory-Verbal Learning Test; BRIEF, behavioral rating inventory of executive function; CFQ, Cognitive Failures Questionnaire; COWA, Controlled Oral Word Association; CVLT-II, California Verbal Learning Test-II; D-KEFS, Delis-Kaplan Executive Function System; FACT-Cog, Functional Assessment of Cancer Therapy–Cognitive; HVLT-R, Hopkins Verbal Learning Test-Revised; MASQ, Multiple Ability Self-Report Questionnaire; RAVLT, Rey's Auditory Verbal Learning Test; TMT, trail making test; UFOV, Useful Field of View test; WCST, Wisconsin card sorting test.
Simone, A. et al. [81]	2023	Not provided.	**Type:** Aerobic exercise (2), computer training program Brain HQ (1), group memory training (1)<br>**Length:** 4 weeks (1), 7-8 weeks (2), 12-months (1)<br>**Dose:** Frequency: once/wk (1), 4 times/wk (1), 5 times/wk (2); Duration: 20-30mins/wk (1), 60 mins/session (2), 90 mins/session (1)	CNSVS, EORTC QLQ-C30, FAB
Singh, N. et al. [82]	2022	USA (4), Canada (1)	**Type:** Exercise (6): Yoga (1), aerobic + resistance training (1), walking (1), moderate intensity physical activity (1), mod or high intensity cycling (1), internet-based tailored exercise program (1); Exercise combined with other (2): social cognitive theory + physical activity (1), exercise + nutrition (1); Cognitive training (2): Nintendo Wii game cognitive training (1), mindfulness-based stress reduction training (1).<br>**Length:** 8 weeks (3), 10-12 weeks (1),12-weeks (4), Not provided (2)<br>**Dose:** Frequency: once/wk (1), twice/wk (2), 3 times/wk (2), 5 times/wk (1), not provided (4); Duration: 30mins per session (1), 90mins per session (2), 120mins per session (2), not provided (5)	Breast Cancer Prevention Trial (BCPT) Cognitive Problems scale, AFI, Stroop test, AMPS, Improvement Oral Symbol Digit

Treanor, C.J. et al. [83]	2016	USA (2), Netherlands (1), Australia (1), Brazil (2), Canada (1), Korea (2), Italy (2), Sweden (1)	**Type:** Aerobic exercise (1), Tibetan sound meditation (1), Memory Attention Adaption Training (1), Computerised cognitive training (1), "Insight" cognitive intervention (1), "ACTIVE" cognitive intervention (1)<br>**Length:** 6-8wks (3), 12 wks (1), 24 wks (1)<br>**Dose:** Frequency: once-twice/wk (3), 4 times/wk (2); Duration: 20-30mins per session (1), 30-50min/session (2), 1-hour per session (2)	FACT-Cog, Squire Subjective Memory Questionnaire, Behavioral Rating Inventory of Executive Functioning, Multiple Abilities Subjective Questionnaire, Delis-Kaplan Executive Function System (D-KEFS), Delis-Kaplan letter fluency test
Van Lonkhuizen, P. J. C. et al. [84]	2019	Not provided.	**Type:** Exercise (2): Combined physical, aerobic, and occupational therapy (1), Home based aerobic exercise (1); Cognitive strategy training (5): Cognitive strategy + problem-solving intervention (1), Holistic mnumonic training (1), Semantic organisation training (2), Goal Management Training (combining mindfulness and strategy training) (1); Cognitive re-training (2): Cognitive training (RehabTr) (1), VR training and computer-assisted retraining (1); Combined cognitive strategy and re-training (3): Strategy training and computerised re-training (1), iPad-based strategy training (ReMIND) (1), Therapist-guided cognitive retraining and strategy training (1)<br>**Length:** 1 wk (2), 2 wks (1), 4 wks (3), 6 wks (1), 8 wks (1), 10 wks (3), 12 wks(1)<br>**Dose:** Frequency: once/wk (4), 3 times/wk (1), 4 times/wk (1), not provided (4)	Not reported.
Vance, D.E. et al. [85]	2017	USA (3), Germany (2), Spain (2), Netherlands (1), Brazil (1), Sweden (1), China (1)	**Type:** Cognitive training (2):Memory training or speed of processing training (1), Cognitive training (1); Compensatory Strategies With Cognitive Training Interventions (5): Psychoeducation group intervention (1), Cognitive Rehabilitation Group intervention (1), Rehabilitation Intervention Program (1), Cognitive-Behavioral Management (1), CBT (1); Exercise (6): Yoga (3), Tai Chi (1), Qigong (1), Aerobic exercise with speed feedback (1); Mindfulness-based (1):Tibetan sound meditation (1); Other (1): Electroencephalographic (EEG) biofeedback (1)<br>**Length:** 4 wks (3), 5 wks (2), 6 wks (1), 6-8 wks (1), 7 wks (1), 8 wks (1), 10 wks (3), 12 wks (3)<br>**Dose:** Frequency: once/wk (7), twice/wk (4), not provided (4); Duration: 20-30mins/session (1), 60 mins/session (3), 90 mins/session (2), 75mins/session (1), 2 hrs/session (1), not provided (7)	Functional Assessment of Cancer Therapy-Cognitive Function; Frontal Assessment Battery; Profile of Mood States.
Vannorsdall, T.D. et al. [86]	2021	USA (12), Belgium (2), Canada (2), Denmark (1), China (3), UK (1), Australia (1), Malaysia (1)	**Type:** Exercise (n=7): Aerobic + resistance (2), aquatic exercise (1), yoga (2), resistance training (1), aerobic training only (1); Psychotherapy (2); Alternative therapies (2): Acupressure (1), Bright light (1)	Piper Fatigue Scale - Cognitive domain, Fatigue Assessment Questionnaire - Cognitive domain, Multidimensional Fatigue Inventory - Cognitive domain, Multidimensional Fatigue Inventory Short Form - Cognitive domain
Von Ah, D. et al. [88]	2014	Not provided.	**Type:** Cognitive training (16), Qigong (1), Tibetan Sound Meditation (1), Biofeedback (1), Tai Chi (1), resistance training (1), aerobic + resistance training (1), mindfulness based stress reduction (1), cognitive rehabilitation (1), natural resorative environment (2).	Not provided.
Zeng, Y. et al. [95]	2020	Not provided.	**Type:** Computerised cognitive training (3), Cognitive rehabilitation (3), Psychoeducation (1), Home based cognitive training (1), Speed of processing training (1), Cognitive health program (1), Group based memory training (1), BrainHQ (1), Memory and adaption attention training (2), Aerobic exercise (5), Cognitive behavioural training (1), Tai Chi/Qigong (3), Meditation /Mindfulness based stressed reduction (2)<br>Yoga (2), Speed feedback therapy on bicycle (1), Accupuncture (1), Supportive	Not provided.

			therapy (1)<br>**Length:**6-weeks (3), 8-weeks (6), 6-month (1), 10-weeks (10), 15-weeks (1), 7-weeks (1), 5-weeks (1), 4-weeks (3), 12-weeks (6), 6-8 weeks (1), 24-weeks (1), 2 sessions (1), Not reported (1)<br>**Dose:** 2 session per week of 90 minutes (2), 2 session per week of 60 minutes (1), 1 session per week for 120 minutes (1), Not reported (12), 1 session per week (2), 2 sessions of 30 minutes (1), 5 session a week of 30 minutes (1), 5 session per week (2), 45-60 minutes per session 2 times per week (1), 2 hours per week (1), 5 sessions per week (1), 40 minutes per session for 60 session (1).	
Zimmer, P. et al. [99]	2016	Not provided.	**Type:** Animals: running wheel (5); Humans: walking and resistance bands (1), Yoga (2), breathing, yoga, and meditation (1), bicycle ergometer (1), Qigong (1), physical activity program (1), strength training (1), aerboic tredmill training (1), Tai chi (1).<br>**Length:** Animals: 3-4 weeks (2), 6 weeks (1), 11 weeks (1), 16 weeks (1);<br>Humans: 4 weeks (2), 6 weeks (1), 10 weeks (2), 12 weeks (4), 6 months (1)<br>**Dose:** Animals: access to running wheel overnight (1), 30min twice daily (1), 8/12 hours per day (1), and timeframe not provided (2); Humans: Frequency: 1 session per week (1), 2 sessions per week (6), 3 sessions per week (1), not provided (2); Duration: 5 mins (1), 10-45 mins (1), 60-75 mins (4), 90 mins (2), not provided (2)	**Animals:** NMTS: Non-Matching to Sample Task, DNMTS: Delayed Non-Matching to Sample task, NOR: Novel Object Recognition; MWM: Morris Water Maze, Open-field test, Hippocampal neurogenesis, Barnes Maze.<br><br>**Humans:** EORTC QLQ-C30: European Organization for Research and Treatment of Cancer, Quality of Life Questionnaire; FACT-Cog: Functional Assessment of Cancer Therapy-Cognitive Function; CRP: C-Reactive Protein; WIT: Wilde Intelligence Subtest, Modified MD Anderson Symptom Inventory, Frontal assessment battery, attention d2 test.
